# The somatic autosomal mutation matrix in cancer genomes

**DOI:** 10.1007/s00439-015-1566-1

**Published:** 2015-05-23

**Authors:** Nuri A. Temiz, Duncan E. Donohue, Albino Bacolla, Karen M. Vasquez, David N. Cooper, Uma Mudunuri, Joseph Ivanic, Regina Z. Cer, Ming Yi, Robert M. Stephens, Jack R. Collins, Brian T. Luke

**Affiliations:** In Silico Research Centers of Excellence, Advanced Biomedical Computing Center, Information Systems Program, Frederick National Laboratory for Cancer Research, Leidos Biomedical Research Inc., P.O. Box B, Frederick, MD 21702 USA; Division of Pharmacology and Toxicology, The University of Texas at Austin, Austin, TX 78723 USA; Institute of Medical Genetics, School of Medicine, Cardiff University, Cardiff, CF14 4XN UK; Masonic Cancer Center, University of Minnesota, 2-120 CCRB, 2231 6th St SE, Minneapolis, MN 55455 USA; US Army Medical Research and Material Command, 568 Doughten Dr., Fort Detrick, Frederick, MD 21702 USA; Naval Medical Research Center-Frederick, 8400 Research Plaza, Fort Detrick, Frederick, MD 21702 USA

## Abstract

DNA damage in somatic cells originates from both environmental and endogenous sources, giving rise to mutations through multiple mechanisms. When these mutations affect the function of critical genes, cancer may ensue. Although identifying genomic subsets of mutated genes may inform therapeutic options, a systematic survey of tumor mutational spectra is required to improve our understanding of the underlying mechanisms of mutagenesis involved in cancer etiology. Recent studies have presented genome-wide sets of somatic mutations as a 96-element vector, a procedure that only captures the immediate neighbors of the mutated nucleotide. Herein, we present a 32 × 12 mutation matrix that captures the nucleotide pattern two nucleotides upstream and downstream of the mutation. A somatic autosomal mutation matrix (SAMM) was constructed from tumor-specific mutations derived from each of 909 individual cancer genomes harboring a total of 10,681,843 single-base substitutions. In addition, mechanistic template mutation matrices (MTMMs) representing oxidative DNA damage, ultraviolet-induced DNA damage, ^5m^CpG deamination, and APOBEC-mediated cytosine mutation, are presented. MTMMs were mapped to the individual tumor SAMMs to determine the maximum contribution of each mutational mechanism to the overall mutation pattern. A Manhattan distance across all SAMM elements between any two tumor genomes was used to determine their relative distance. Employing this metric, 89.5 % of all tumor genomes were found to have a nearest neighbor from the same tissue of origin. When a distance-dependent 6-nearest neighbor classifier was used, 86.9 % of all SAMMs were assigned to the correct tissue of origin. Thus, although tumors from different tissues may have similar mutation patterns, their SAMMs often display signatures that are characteristic of specific tissues.

**Electronic supplementary material:**

The online version of this article (doi:10.1007/s00439-015-1566-1) contains supplementary material, which is available to authorized users.

## Introduction

Cancer is promoted by a diverse set of genetic and epigenetic alterations in the soma, including single-base substitutions (SBSs), insertions and deletions, chromosome and DNA segment copy number variations (CNV), as well as chromosomal translocations and rearrangements. Next-generation sequencing has become a powerful tool for identifying these alterations (Meyerson et al. 2010), providing an unprecedented opportunity to further our understanding of tumorigenesis. The mutations in each tumor genome reflect the net contribution from each of the individual mutational mechanisms that played a role in the onset of disease and its subsequent development (Stratton 2011), modified by the influence of cellular processes such as DNA replication (Lawrence et al. 2013), transcription, and the DNA repair pathways (Vogelstein et al. 2013). Whereas “driver” mutations enable positive selection, “passenger” mutations are, by definition, simply tolerated and provide no proliferative advantage or disadvantage to tumor cells (Stratton et al. 2009; Vogelstein et al. 2013); the molecular mechanisms leading to the generation of driver and passenger mutations are however expected to be similar. Hence, because passenger mutations vastly outnumber driver mutations, in the absence of selection the overall SBS mutation pattern is believed to capture the composite history of the mutational processes that acted upon the tumor cells. Mutational patterns are in turn determined by chemical reactions, not only with respect to initial base modification by chemical or enzymatic activity (e.g., cytosine deamination) but also through subsequent interactions with DNA repair mechanisms, as well as long-range interactions at both intermolecular and atomic levels, such that these patterns may be heavily influenced by the local nucleotide sequence context (Holmquist and Gao 1997; Pfeifer et al. 2005). Indeed, sequence-specific mutational biases in germline mutational spectra (Cooper et al. 2011), and more specifically in genes implicated in tumorigenesis (Ivanov et al. 2011), have been shown to be consequent to the basic properties of a range of different mutational mechanisms (Bacolla et al. 2014; Helleday et al. 2014).

Although many mutational processes generally manifest simultaneously within a tumor, UV-induced DNA damage has been specifically implicated in melanoma and other skin cancers (Armstrong and Kricker 2001; Hodis et al. 2012; Wikonkal and Brash 1999). It comprises a set of signature mutations that result from the formation of photoproducts, such as cyclobutane pyrimidine dimers (CPDs) and pyrimidine 6-4 pyrimidone photoproducts at two adjacent pyrimidines (Banerjee et al. 1988; Beauchamp and Lacroix 2012). The majority of UV-induced damage is CPD mediated (Pfeifer and Besaratinia 2012). It is well established that nucleotide excision repair (NER) represents the main pathway for correcting CPDs (Batty and Wood 2000). However, NER proteins display strong sequence-dependent biases in the repair rates of CPDs (Holmquist and Gao 1997; Suter et al. 2000; Tornaletti and Pfeifer 1994), which serve to influence the final (i.e., observable) mutational spectrum.

Oxidative DNA damage originates endogenously from reactive oxygen species and exogenously from ionizing radiation and certain chemicals (Bacolla et al. 2014; Dizdaroglu 2012; Maynard et al. 2009). One of the most common oxidative base modifications, 8-oxo-7,8-dihydroguanine, is highly mutagenic (Dizdaroglu 2012; Grollman and Moriya 1993; Maynard et al. 2009) and is excised by the base excision repair (BER) pathway (Maynard et al. 2009). Signatures of oxidative damage have been attributed to G → T, G → C and A → T transversions, as well as G → A transitions, in various tumor types, including lung cancer (Lee et al. 2010; Pleasance et al. 2010b) and melanoma (Agrawal et al. 2011; Pleasance et al. 2010a).

5-Methyl-CpG (^5m^CpG) dinucleotides have been firmly established as hotspots of gene mutation in human pathology, both in the germline (Cooper et al. 2010) and the soma (Pfeifer 2006), and specifically including tumor suppressor genes (Mort et al. 2008). Mutation at this doublet is characterized by C → T transitions (i.e., TpG on one strand and CpA on the complementary strand) following spontaneous ^5m^C deamination or oxidation involving thymine glycol intermediates (Bacolla et al. 2014; Lee and Pfeifer 2003; Rubin and Green 2009; Yoon et al. 2001).

A number of cancer genome sequencing projects have reported the absolute numbers of single-nucleotide transitions (C → T, T → C) and transversions (G → C, G → T, A → C, A → T) in various tumors (Agrawal et al. 2011; Bueno et al. 2010; Chapman et al. 2011; Lee et al. 2010; Pleasance et al. 2010a, b; Turajlic et al. 2012). However, attempts to dissect cancer genomic mutational spectra to reveal the underlying mutational processes (Nik-Zainal et al. 2012; Pfeifer 2010; Pfeifer and Hainaut 2011; Stephens et al. 2012) have proven to be extremely challenging.

Various studies have proposed the use of ‘mutation landscapes’ as a means to infer the nature of the mutational processes underlying tumorigenesis (Alexandrov et al. 2013a, b; Burns et al. 2013; Lawrence et al. 2013; Pfeifer and Besaratinia 2009; Roberts et al. 2013). In these studies, sites of single-base substitutions (SBSs) were analyzed either in a sequence-independent context (Lawrence et al. 2013; Roberts et al. 2013), in the center of trinucleotide motifs (Burns et al. 2013; Nik-Zainal et al. 2012), or at the second position of tetranucleotide sequences (Bacolla et al. 2013). The trinucleotide motif pattern can be represented as a 96-element vector with 32 unique trinucleotides and three possible mutations of the central element. Following this representation, more than 20 distinct mutational signature patterns were recognized using nonnegative matrix factorization (Alexandrov et al. 2013a, b); some of these signatures contained components that could be associated with specific mutational processes (Helleday et al. 2014).

An early investigation examined the effect of neighboring nucleotides on mutation frequencies in human germline exonic mutations (Krawczak et al. 1998), and determined that nucleotides two positions upstream of the mutation site were capable of exerting a significant effect on both mutation type and frequency. Activation-induced deaminase (AID) is known to cause cytosine mutations at WRC motifs (W is a weak acid, A or T, and R is a purine), suggesting that the nucleotide two bases upstream of the mutation site is important (Carpenter et al. 2010). An examination of inherited mutations from reconstructed ancestral states identified 3.5- and 3.3-fold excesses of T → C transitions at the second position of ATTG and ATAG motifs, respectively, and a 3.4-fold excess of A → C transversions at the first position of ACAA motifs (Panchin et al. 2011). Likewise, we have previously reported that in melanoma guanines at GRA motifs undergo ~twofold more frequent substitutions than guanines at GR(C|T|G) motifs (Bacolla et al. 2013). Finally, SBSs along mononucleotide repeats are often heavily influenced by long-range interactions caused by charge transfer mechanisms along the DNA (Bacolla et al. 2015).

Thus, nucleotides two or three base-pairs away from of the mutation site appear to exert an influence on the mutation, implying that modeling single-base substitutions at the central position of trinucleotide motifs may be inadequate to the task of fully describing the effects of distal nucleotides on the mutational processes.

Herein, we use a pentanucleotide (which includes two nucleotides upstream and downstream of the mutation) as the basic motif and a 32 × 12 somatic autosomal mutation matrix (SAMM) to capture the overall mutation pattern within various types of cancer. We present the SAMMs obtained from autosomal somatic mutations in 909 different cancer genome samples from 21 publically available whole-genome sequencing datasets, comprising a total of 10,681,843 single-base substitutions (Table 1). We also derive mechanistic template mutation matrices (MTMMs) representing estimated mutation patterns putatively emanating from the four mutational mechanisms studied here: oxidative damage, UV-induced damage involving CPD formation, deamination of ^5m^CpG, and the action of members of the APOBEC family of cytosine deamination enzymes. By comparing the MTMMs against the overall SAMM from each cancer sample, we were able to infer the likely maximum contribution of each mutational mechanism in each case.

**Table 1:** Details of the 21 whole-genome sequencing datasets examined in this study

Dataset	Label	#Samples	Total #SBSs	Source^a^
Acute lymphoblastic leukemia	ALL	1	7442	Sanger
Acute myeloid leukemia (South Korea)	LAML-KR	4	377,876	ICGC
Breast triple negative/lobular cancer	BRCA-UK	18	165,808	ICGC
Breast cancer	Breast	77	534,046	Sanger
Esophageal adenocarcinoma	ESAD-UK	16	290,325	ICGC
Liver cancer NCC	LINC-JP	31	329,052	ICGC
Liver cancer RIKEN	LIRI-JP	188	1,922,567	ICGC
Liver cancer	Liver	84	790,487	Sanger
Lung adenocarcinoma	Lung_Adeno	23	1,386,149	Sanger
Malignant lymphoma DKFZ	MALY-DE	37	267,052	ICGC
Melanoma	Melanoma	25	1,841,735	(Berger et al. 2012)
Ovarian cancer QCMG	OV-AU	89	833,427	ICGC
Pancreatic cancer OICR	PACA-CA	45	333,342	ICGC
Pancreatic cancer QCMG	PACA-AU	137	954,081	ICGC
Pancreatic cancer endocrine neoplasms QCMG	PAEN-AU	12	430,28	ICGC
Pancreatic cancer	Pancreas	14	103,032	Sanger
Medulloblastoma	Medulloblastoma	11	35,125	Sanger
Pediatric brain tumors BMBF	PBCA-DE	16	46,147	ICGC
Prostate adenocarcinoma	PRAD-UK	3	18,763	ICGC
Prostate cancer	Prostate	7	21,603	Sanger
Renal clear cell carcinoma	RECA-EU	71	379,756	ICGC
Total		909	10,681,843	

Sanger: ftp://sanger.ac.uk/pub/cancer/AlexandrovEtAl/somatic_mutation_data/

^a^ICGC: ftp://data.dcc.icgc.org/current/

## Methods

### SAMM generation

To illustrate the procedure used to generate the somatic autosomal mutation matrix (SAMM), the following example of a single-base substitution (SBS) is provided: CTGAT → CTAAT. In previous studies that looked at mutations in the central position of the trinucleotide (Burns et al. 2013; Nik-Zainal et al. 2012), the SBS TGA → TAA would have been used. To allow for redundancy (TGA → TAA is the same as the complementary TCA → TTA), the 32 unique trinucleotides require a purine (A or G) in the central position being mutated. This is presented as a 96-element array (32 unique trinucleotides with three possible mutations at the central position). The SAMM uses the same set of 32 trinucleotides, but mutations are allowed at all three positions. For CTGAT → CTAAT, the first trinucleotide represents the first three nucleotides of the pentanucleotide and the mutation is CTG → CTA. Since the central nucleotide is a pyrimidine, the reverse complement is used and the mutation is stored as CAG → TAG and is denoted by CAG.1t (1t denotes that the first nucleotide has been mutated to a thymine). The second trinucleotide represents the central three nucleotides of the pentanucleotide and the SBS is TGA → TAA (denoted TGA.2a), and the third trinucleotide represents the SBS GAT → AAT (denoted GAT.1a). The overall SAMM is given by a 32 × 4 dimensional matrix where each column represents a unique trinucleotide and each row displays each of the four possible mutations at each of the three positions. Since a specific nucleotide cannot be mutated to itself, any given SAMM has three zero elements in each row, yielding a total of 288 (32 × 9) non-zero elements. Whereas the SAMM contains three times as many non-zero elements as the 96-element vector, each mutation contributes three elements within the SAMM, thereby expanding the information on the local nucleotide environment surrounding the SBS without requiring any increase in the overall number of mutations.

The reference human genome assembly hg19 was used to capture the pentanucleotide centered around each selected SBS. For each of the three trinucleotides representing a mutation, a count matrix was increased by one. Once all SBSs were processed, the counts were divided by the number of times each trinucleotide appeared in hg19 to obtain mutation frequencies. The final SAMM was obtained by scaling the frequency matrix, so that the sum of all elements equaled unity (Table 2).

**Table 2:** SAMM for the acute lymphoblastic leukemia sample PD4020a

Motif	1.a	2.a	3.a	1.t	2.t	3.t	1.c	2.c	3.c	1.g	2.g	3.g
AAA	0	0	0	0.000461	0.000546	0.000649	0.000188	0.000154	0.000256	0.000154	0.000154	0.000324
AAT	0	0	0.000264	0.000448	0.000264	0	0.000105	0.000158	0.000501	0.000158	0.000501	0.00029
AAC	0	0	0.00108	0.000405	0.000405	0.002611	0.000135	0.000135	0	0.000135	0.000585	0.000675
AAG	0	0	0.007979	0.000099	0.000493	0.001543	0.000033	0.000296	0.018453	0.00023	0.000328	0
AGA	0	0.023608	0	0.000267	0.00234	0.000326	0.000089	0.044194	0.000178	0.000385	0	0.000326
AGT	0	0.000488	0.000204	0.000326	0.000936	0	0.000448	0.001303	0.000204	0.000488	0	0.000163
AGC	0	0.000979	0.000606	0.000233	0.000699	0.001305	0.000093	0.000699	0	0.000093	0	0.000373
AGG	0	0.001615	0.004405	0.000294	0.000661	0.001468	0.00022	0.000771	0.004552	0.00022	0	0
TAA	0.000504	0	0	0	0.00041	0.000473	0.000252	0.000126	0.000189	0.000189	0.000032	0.000221
TAT	0.000319	0	0.000351	0	0.000256	0	0.000192	0.000288	0.000319	0.000256	0.000319	0.000192
TAC	0.000232	0	0.00081	0	0.000405	0.001389	0.000289	0.000058	0	0.000116	0.000347	0.000984
TAG	0.000255	0	0.007126	0	0.000356	0.001476	0.000204	0.000153	0.018986	0.000051	0.000305	0
TGA	0.000167	0.054179	0	0	0.004883	0.000201	0.000368	0.046353	0.000067	0.000201	0	0.000468
TGT	0.000293	0.001073	0.000358	0	0.000976	0	0.00013	0.000943	0.000586	0.000293	0	0.000163
TGC	0	0.001179	0.000816	0	0.001179	0.001587	0.000181	0.000408	0	0.000091	0	0.000499
TGG	0.000106	0.002586	0.003189	0	0.000957	0.001169	0.000106	0.001453	0.005137	0.000142	0	0
CAA	0.001803	0	0	0.012343	0.000139	0.000312	0	0.000139	0.000104	0.024062	0.000139	0.000277
CAT	0.002253	0	0.000179	0.017593	0.00025	0	0	0.000358	0.00025	0.009476	0.00025	0.000179
CAC	0.001607	0	0.000956	0.012946	0.000043	0.001868	0	0.000087	0	0.005951	0.000217	0.001129
CAG	0.002477	0	0.010743	0.019492	0.000161	0.000965	0	0.000161	0.012223	0.011869	0.000193	0
CGA	0.000295	0.035959	0	0.020927	0.002063	0.000295	0	0.007958	0.000295	0.003832	0	0.000589
CGT	0.000776	0.024316	0	0.016814	0.000259	0	0	0	0	0.001811	0	0
CGC	0.001084	0.011925	0.000271	0.019784	0	0.001897	0	0.000271	0	0.001626	0	0.000813
CGG	0.000468	0.009822	0.003976	0.021749	0.000468	0.000234	0	0.000468	0.002339	0.000935	0	0
GAA	0.037661	0	0	0.004192	0.000233	0.000133	0.032704	0.000067	0.0001	0	0.000366	0.000566
GAT	0.024472	0	0.000246	0.002948	0.000295	0	0.032236	0.000098	0.000393	0	0.000393	0.000246
GAC	0.015498	0	0.000554	0.00173	0.000208	0.001661	0.018681	0.000277	0	0	0.000415	0.000761
GAG	0.037753	0	0.006079	0.003291	0.000116	0.001123	0.05026	0.000194	0.009487	0	0.000426	0
GGA	0.002454	0.012271	0	0.00055	0.002708	0.000042	0.000677	0.012779	0.000169	0	0	0.000381
GGT	0.001518	0.000562	0.000225	0.000956	0.000787	0	0.000899	0.00045	0.000394	0	0	0.000056
GGC	0.001037	0.000819	0.000873	0.000437	0.000382	0.001965	0.000437	0.000164	0	0	0	0.000327
GGG	0.003413	0.000693	0.005046	0.000742	0.000297	0.000841	0.001435	0.000247	0.001929	0	0	0

Fractional mutation frequencies (7442 SBSs)

Only autosomal somatic mutations were considered, since the occurrence of each trimer in hg19 would have to be determined individually for males and females, and in many cases the gender of the individual providing the original tumor sample was unknown. In addition, pentamers were allowed to contain only one mutation; hence, the number of SBSs examined for each dataset was fewer than the number in the original dataset. Preliminary bootstrap sampling from a large sample dataset showed that 2000 SBSs were needed to achieve a 97.5 % confidence level that the SAMM was within a Manhattan distance of 0.156 to the SAMM representing the mutational processes of the sample (data not shown). Thus, the minimum number of SBSs used to generate a SAMM was 2000.

### MTMM generation

Analysis of SBSs at the trinucleotide level of granularity allows for the differentiation of underlying mutational mechanisms (Nik-Zainal et al. 2012). We used a similar 32 × 12 matrix to develop models in the form of mechanistic template mutation matrices (MTMMs) representing four canonical mechanisms known to cause somatic mutations: (i) oxidative DNA damage, which accounts for mutations resulting from oxidation reactions and direct or indirect ionizing radiation; (ii) UV-induced DNA damage, which includes mutations arising from CPDs; (iii) ^5m^CpG deamination, which models mutations mediated by the deamination of ^5m^C; and (iv) the action of the APOBEC family of cytosine deamination enzymes.

The most common mutations caused by oxidative damage are G → T (C → A) transversions, although G → C (C → G) transversions and G → A (C → T) transitions are also observed (Dizdaroglu 2012). It has been previously shown that one electron oxidation correlates with the vertical ionization potential (VIP) of DNA bases (Senthilkumar et al. 2003), and in this investigation, we assume that mutations caused by oxidative damage follow first-order kinetic with an activation energy (*E*_a_) equal to the VIP. Therefore, the mutation frequency is proportional to *e*^(−VIP/*kT*)^, where *k* is Boltzmann’s constant and *T* is the temperature in degree Kelvin.

The susceptibilities of the 32 unique trinucleotides to oxidation were estimated by calculating vertical ionization potentials (VIPs) for DNA trimer fragments, which included DNA backbone and sodium counter-ions. Three-dimensional structures of the 32 possible DNA double-stranded trinucleotides were built using w3DNA (Zheng et al. 2009). Hydrogen atoms, atomic charges, and charged sodium counter-ions were assigned according to the amber99 force field (Wang et al. 2000) using UCSF CHIMERA (Yang et al. 2011). Sodium counter-ions were positioned next to the four DNA backbone phosphates. The ground state structure of each trinucleotide was energy-minimized in vacuo using a 10,000-step steepest-descent algorithm and the amber99 force field in GROMACS 4.5.1 (Hess et al. 2008). Cutoffs of 10 and 14 Å were used for Coulombic and van der Waals interactions, respectively.

VIPs were computed using Kohn–Sham density functional theory (Kohn et al. 1996), whereby hydrogen bonding and stacking interactions between base-pairs (Ribeiro et al. 2011) were modeled by employing the Minnesota M06-2X density functional (Zhao and Truhlar 2008a, b), as implemented in the GAMESS electronic structure package (Schmidt et al. 1993). As a first step, we tested the reliability of the M06-2X functional for the prediction of gas-phase VIP of natural guanine and adenine against a variety of basis sets (Dunning 1989; Harihara and Pople 1973) ranging from small (6-31G(d)) to large (cc-pVQZ) (Table 3). High accuracy was obtained with the biggest basis set, 8.22 eV (predicted) vs. 8.24 eV (experimental) for guanine, and 8.48 eV (predicted) vs. 8.44 eV (experimental) (Orlov et al. 1976) for adenine. The smaller 6-31G(d) basis also provided excellent estimates of 8.02 and 8.32 eV for guanine and adenine, respectively. Thus, the 6-31G(d) basis set was employed because it was practical and adequate for assessing the relative VIP differences between DNA fragments.

**Table 3:** Computed and experimental ionization potentials of guanine and adenine using different basis sets

Basis set	Guanine	Adenine
6-31G(d)	8.02	8.32
cc-pVDZ	8.01	8.32
cc-pVTZ	8.19	8.45
cc-pVQZ	8.22	8.48
Expt.	8.24	8.44

VIP values (Table 4) were used to determine mutation frequencies at guanines and adenines. For mutations at the guanine base, we assigned G → T, G → A, and G → C and their complements (C → A, C → T, and C → G) an experimentally observed ratio of 8:3:1, respectively (Kamiya et al. 1992). This ratio accounts for the bias of G → T (C → A) transversions and allows us to relate different types of mutations to the G → T transversions. Since guanine is the most easily oxidized base, a ratio of 1/20, estimated from the experimental standard ionization potentials of adenine and guanine, was used to add adenine mutations (Bushnell et al. 2011). This first-level approximation does not take into account A → T (T → A) transversions (although these mutations have been observed experimentally (Dizdaroglu 2012), due to the lack of an experimentally observed ratio relating A → T transversions to A → C transversions. These mutation frequencies were then scaled so that they sum to 1.0. The resulting oxidative damage (OxD) MTMM is given in Supplemental Table 1a, and a heatmap is shown in Supplemental Figure 1a.

**Table 4:** Computed vertical ionization potential (VIP) and vertical singlet excitation energy (VSEE) of the most likely pyrimidine *π*–π* transition (among the lowest three) for each of the DNA fragments

Guanine-centered			Adenine-centered		
Sequence (5′-NGN-3′)	VIP (eV)	VSEE (eV)^a^	Sequence (5′-NAN-3′)	VIP (eV)	VSEE (eV)^a^
GGG	5.39	6.40 (6)	GAG	5.74	6.34 (5)
GGA	5.50	6.37 (5)	GAC	5.88	6.34 (5)
GGT	5.54	6.37 (5)	GAA	5.89	6.29 (3)
AGG	5.57	6.39 (5)	CAG	5.91	6.35 (5)
TGG	5.59	6.39 (5)	GAT	5.91	6.34 (4)
CGG	5.60	6.36 (5)	AAG	5.92	6.22 (3)
GGC	5.63	6.37 (6)	CAC	5.92	–
TGA	5.64	6.26 (3)	TAG	5.93	6.35 (4)
AGA	5.66	6.32 (5)	CAT	6.04	–
CGA	5.79	6.31 (4)	TAC	6.05	–
AGT	5.81	6.29 (4)	CAA	6.05	6.23 (2)
CGT	5.86	–	AAC	6.11	6.32 (4)
CGC	5.88	–	AAA	6.37	6.27 (3)
TGT	5.90	–	TAA	6.51	6.24 (3)
AGC	5.95	6.32 (5)	AAT	6.52	6.23 (3)
TGC	5.97	–	TAT	6.55	–

^a^The number of the excited state (ground state = 0) is given in parentheses

To model the likelihood of photoexcitation-mediated pyrimidine dimerization for each of the 24 unique trinucleotide sequences containing pyrimidine–pyrimidine steps, we assumed that the rate-limiting step for this first-order process was a *π*–π* transition of a pyrimidine base. This was performed by calculating the vertical singlet excitation energy (VSEE) corresponding to the most likely *π*–π* electronic transition at one or more neighboring pyrimidines. The mutation frequency for a given trinucleotide sequence is therefore proportional to *e*^(−VSSE/*kT*)^.

Computations were performed for DNA trimer fragments with the DNA backbone without sodium counter-ions, since the counter-ions had low-energy unoccupied 3s/3p orbitals that gave rise to many low-lying excited states having (i) the wrong character and (ii) essentially zero intensity or likelihood of transition. VSEEs of the sodium-free DNA trimer fragments were computed by CIS (Foresman et al. 1992) using the 6-31G(d) basis set, as implemented in the GAMESS. Although the CIS method is known to consistently overestimate vertical excitation energies (Webb 2006), it is the only practical quantum mechanical approach to model the large systems employed here. Since our goal was to compare the relative excitation energies of the DNA fragments, the 6-31G(d) basis set was used. The most likely *π*–π*-type electronic transition occurring on one or more neighboring pyrimidines must be identified to model the relative probability of pyrimidine dimerization within a DNA trimer. As each DNA trimer contains exactly three pyrimidines, there will be three corresponding low-lying *π*–π*-type singlet excited states. Thus, for each DNA trimer, we identified the three lowest excited states that were of pyrimidine character by examining the nature of the molecular orbitals characterizing the excited state. We then determined which of these three pyrimidine-associated excited states had the highest oscillator strength representing the intensity, or likelihood, of transition.

Nucleotide excision repair (NER) is highly biased in terms of its sequence context dependence in the efficiency of CPD repair (Cai et al. 2009; Holmquist and Gao 1997; Kunkel 2011; Suter et al. 2000; Tornaletti and Pfeifer 1994). Therefore, we combined the VSEE values (Table 4) with experimentally derived sequence-dependent NER efficiencies to compute the UV-induced DNA damage model matrix elements. The resulting UV-induced DNA damage (CPD) MTMM is given in Supplemental Table 1b, and a heatmap is shown in Supplemental Figure 1b.

Mutations arising from ^5m^CpG deamination did not require molecular modeling. Hence, we assigned equal probabilities to C → T (and G → A on the opposite strand) transitions at CpG sites. While other motifs such as CHG (where H = A, C, or T) sites are also methylated in the human genome and can also give rise to deamination (Cooper et al. 2010; Lister et al. 2009), these events are comparatively rare and would not have contributed appreciably to our model. Hence, the deamination model is currently confined to ^5m^CpG dinucleotides and involves only four of the 32 possible trinucleotides (CGN). The resulting MTMM representing ^5m^CpG deamination (CpG) is given in Supplemental Table 1c, and a heatmap is shown in Supplemental Figure 1c.

The action of the APOBEC family of cytosine deaminases has been shown to generate clusters of mutations (Alderton 2012; Lada et al. 2012), in addition to isolated mutations (Roberts and Gordenin 2014a). To generate an APOBEC MTMM, each dataset was examined to find regions where more than five sequential mutations had an average spacing of 1 kb or less (Alexandrov et al. 2013a). Mutations within these regions were examined and a potential APOBEC pattern was determined if at least 50 % of all mutations within the region were TC(A|T) → T(T|G)(A|T) SBSs (Roberts and Gordenin 2014b) on the reference or complementary strand. Within each putative ABOBEC cluster, all NTC(A|T)N pentanucleotides were stored, as well as the observed variant nucleotide (T|G). A total of 316 cancer genomes contained at least one putative APOBEC cluster, comprising 8,504 possible APOBEC mutations. Each stored pentanucleotide was then used to update the APOBEC MTMM for each of the three composite trinucleotides (NTC, TC(A|T), and C(A|T)N). The resulting MTMM representing cytosine deamination by APOBEC is given in Supplemental Table 1d, and a heatmap is shown in Supplemental Fig. 1d.

### Maximum contribution of MTMMs

Given a SAMM from a tumor sample, the maximum contribution from each mutational mechanism was determined by identifying each trimer element in the MTMM that contributed at least 1 %. The ratio of the observed scaled frequency from the sample to the mechanistic scaled frequency yields a component factor. Therefore, the minimum factor across all elements of the MTMM represents the maximum possible contribution of that mechanistic template to the sample’s SAMM. In other words, if the MTMM is multiplied by this maximum contribution and subtracted from the sample’s SAMM, at least one of the elements associated with this mechanism will be reduced to zero.

### Comparison and display of SAMMs

The heatmap of a given SAMM was drawn using an in-house program that generates input for the imaging program *fly* (http://martin.gleeson.com/fly/). The scaled mutation frequencies were multiplied by 100 and used to determine the intensity of the red color. A value above 6.5 yields full red, 6.5–5.5 gives a slightly weaker red and so on, until values below 0.5 are white. With this procedure, the heatmaps for all SAMMs employ the same scale and hence can be directly compared.

A comparison of different SAMMs from the same cancer genome dataset was performed using unweighted average linkage hierarchical clustering. Clustering and the resulting dendrogram were produced by the program Multidendrogram (Fernandez and Gomez 2008). Comparing two SAMMs, one has the option of either measuring the similarity between SAMMs or their difference. A similarity metric could be (1 − *r*), where *r* is the Pearson correlation coefficient, or could be obtained by measuring the difference in the projection of each sample’s SAMM onto one or more template SAMMs, such as the MTMMs outlined above. If a given MTMM dominates in many different samples’ SAMMs, such as from ^5m^CpG deamination, then these SAMMs would appear to be very similar. In the analysis presented here, the difference between SAMMs is used. The distance between SAMMs is determined by the Manhattan distance between them, which represents the sum of the absolute difference in scaled frequencies over all matrix elements. If the contribution of a given mutational MTMM dominates the SAMMs of two different samples, these effects will tend to cancel each other out and other SBSs that populate different elements of the SAMMs will determine their relative distance.

An in-house program was written in Fortran to calculate the Manhattan distance between all pairs of mutation landscapes from a list of landscapes and to construct a distance matrix that could be further processed by the program. In cases where the number of samples was too large for the dendrogram to provide sufficient information about their relative difference, a Sammon map was produced instead. A Sammon projection (Sammon 1969) attempts to map the distribution of objects from high dimensional space into lower dimensional space by placing the objects in a distribution that minimizes

$$ \mathop \sum \limits_{i < j} \frac{{\left( {d_{i,j} - d_{i,j}^{*} } \right)^{2} }}{{d_{i,j}^{*} }} $$

In this equation, $d_{i,j}^{*}$ is the actual distance between a pair of objects in higher dimensional space and $d_{i,j}$ is the approximate distance after mapping to lower dimensional space. The relative error squared is then summed over all pairs of objects. The actual distances are their Manhattan distances and the approximate distances are their Euclidean separations after mapping to two-dimensional space.

A near-optimal placement of each object was determined using an in-house Evolutionary Programming algorithm. A putative solution represents a given position of each sample in two dimensions, and the cost of this solution is the Sammon score described above. Each putative solution (parent) generates a new putative solution (offspring), randomly moving a small number of samples in the two-dimensional plane. Initially, a sample could be moved by up to 10 % of the maximum inter-landscape distance in either direction, which was reduced by 2 % every 1000 generations. The cost of each offspring is its Sammon score. Once all parent solutions have generated an offspring solution, the 16,000 putative solutions are examined and the 8000 solutions with the lowest cost become the parents for the next generation. In other words, deterministic selection is employed. At the end of the final generation, the two-dimensional mapping of the samples with the lowest Sammon score represents the Sammon map of the relative orientation of the samples such that their distances are preserved to the greatest possible extent. The simulation we employed used a population size of 8000, which proceeded for 40,000 generations.

Since the Sammon score described above behaves very differently for distances above 1.0 compared to distances below 1.0, all values in the distance matrix were multiplied by 100 before the mapping began. The program displays the final positions of the objects by generating an input file to the graphics program *fly*.

### Determining the tissue of origin

The first examination simply identified the most similar SAMM to a given SAMM and determined if they corresponded to the same tissue. This was performed using the Manhattan distances between pairs of SAMMs. A more extensive test used a distance-dependent *k*-nearest neighbor classifier to predict the tissue of origin for each SAMM. If *S*_*i*_ is the *i*th nearest SAMM to the given one, with a distance of *D*(*S*_*i*_), the probability that it corresponds to the same tissue, *P*(*T*(*S*_*i*_)), is given by

$$ P\left( {T\left( {S_{i} } \right)} \right) = \frac{\beta }{{D\left( {S_{i} } \right)}} $$

In this equation, *β* is equal to *D*_0.5_/2, where *D*_0.5_ is the distance where the probability of corresponding to this tissue equals 0.5. Each of the *k*-nearest neighbors was used to increment the probabilities of corresponding to their respective tissues. To ensure that an outlier was not forced to belong to a tissue group, an extra probability was included. This probability, labeled *P* (und), is the probability that the predicted tissue for a given SAMM is Undetermined, and is given an overall value of *k* (0.1). This means that for the *i*th nearest neighbor SAMM, if the probability of corresponding to a given tissue is 0.1, there is an equal probability that the SAMM corresponds to an Undetermined tissue type.

Once the probabilities of corresponding to each tissue type were determined, they and *P*(und) were scaled so that the sum of the probabilities was 1.0. In the first prediction of the tissue of origin, the given SAMM was assigned to the *j*th tissue type, *T*_*j*_, if *P* (*T*_*j*_) was at least 0.5. If no value of *P* (*T*_*j*_) was at least 0.5, the predicted tissue type of this SAMM was Undetermined. The second prediction used a Maximum Likelihood assignment where the predicted tissue of origin was simply the one with the largest *P* (*T*_*j*_), or Undetermined if *P* (und) was larger than any *P* (*T*_*j*_).

## Results and discussion

In this study, we make use of quantum mechanical calculations to derive VIP and VSEE data for specific DNA sequences, and then use this information to obtain frequencies of SBSs in cancer genomes. The rationale for applying VIP values to address the issue of SBSs in cancer genomes is based on a large number of theoretical and experimental studies performed during the past 30 years on short DNA oligomers (Kanvah et al. 2010; Saito et al. 1998; Yoshioka et al. 2003). This composite work has led to the conclusion that chemical reactivity of DNA bases to attacking oxidants is influenced by the energy required to abstract an electron from the DNA, and that the values of this energy are strongly sequence context dependent, being influenced by the differential ability of electrons to fastly migrate from one base to another based on the types of the DNA bases. We have recently applied this knowledge to establish correlations between VIPs and mutation frequencies in cancer genomes (Bacolla et al. 2013), and now expand these relationships to infer quantitatively contributions to mutagenesis in cancer genomes. We apply the same rationale to VSEE, although in this case we are not aware that correlations with UV-induced mutations have already been reported.

The individual samples from each dataset were used to construct a SAMM and the Manhattan distance between each SAMM was determined (Supplemental Table 2a–t); no entry is given for the single cancer genome sample in the acute lymphoblastic leukemia (ALL) dataset. If more than two samples per dataset were present, then the samples were clustered. The ensuing dendrograms are shown in Supplemental Figure 2a–t, the heatmaps and top five mutation frequencies are reported in the Supplemental Figures, and the maximum contribution of each MTMM to the overall SAMM for each cancer genome is presented in Supplemental Table 3a–u. A detailed discussion of each dataset in Table 1 is provided in the Supplemental Results and Discussion. Here, we focus on the results for the full set of 909 cancer genome samples from the 21 datasets by discussing the maximum possible contributions from the four mutational mechanisms, and addressing the question as to whether the differences in SAMMs may be used to deduce the tissue of origin for each sample.

### Maximum contributions from the four mutational mechanisms

The extent to which the four mutational mechanisms accounted for the total SAMM of a sample varied greatly. For example, in the SAMM for DO49436 (PACA-CA), the four canonical mechanisms combined to account for 58.3 % of the scaled mutation frequencies, although a large proportion (43.9 %) was conferred by the ^5m^CpG deamination template. We realize that these estimates are only an approximation since the assumption that each mutational mechanism contributed to different and non-overlapping elements of the SAMM is unlikely to be the case, while other mutational mechanisms, which we have not considered here, may also be operational. At one extreme, 78 of the cancer genomes could have 50 % or more of the mutation frequencies in their SAMMs accounted for by the set of four canonical mutational mechanisms. Of these 78 cancer genomes, 65 were pancreatic cancers. At the other extreme, in 27 of the 909 SAMMs, fewer than 20 % of all mutation frequencies could be attributed to these mutational mechanisms. Contained within this latter set were 21 of the 25 melanoma cancer samples.

In 18 of the SAMMs examined, up to 25 % of all scaled frequencies matched the oxidative damage MTMM, and all but three (83.3 %) were associated with lung adenocarcinomas; the remaining three were renal cancers. The oxidative damage MTMM was constructed from first principles using vertical ionization potentials as the rate-limiting step; finding large contributions in the lung cancers examined was a confirmatory observation. The maximum contribution attributed to the UV-induced damage template was small across all SAMMs, the highest value being 13.2 % in DO45169, from the LIRI-JP_icgc dataset. UV-induced damage is expected to make a significant contribution to the SAMMs of skin cancers but, as mentioned above, these types of cancer display SAMMs that are not well described by any of our canonical mechanisms. Interestingly, 23 of the top 25 (92 %) were liver cancers. Thus, it is possible that (1) the MTMM for UV-induced damage via CPD formation is insufficient to describe this process; (2) the NER-dependent repair corrections we have introduced correlated poorly with cancer biology; or (3) specific hotspots, such as GRA (R = A|G) motifs (Bacolla et al. 2013), should be given additional weight.

The SAMMs from 94 different cancer genomes had a maximum contribution from ^5m^CpG deamination MTMM of at least 35 %. Of these, 82 (87.2 %) were pancreatic cancers as well as seven of the 16 pediatric brain tumor genomes. In 28 cancer genomes, the maximum contribution of this mechanism was less than 5 %.

There also appeared to be strong tissue specificity in relation to the putative maximum contribution of cytosine deamination by APOBEC enzymes. In the 28 SAMMs where 10 % or more of the total scaled frequencies were consistent with this mechanism, 21 were breast cancers. By contrast, there were 55 cancer genomes whose SAMMs contained a maximum contribution of less than 1 % from the APOBEC-signature MTMM.

### Inferring the cancer tissue of origin

Although some tissue specificity was evident for each of the four mutational mechanisms, the majority of all mutation frequencies were not accounted for by any of these mutational mechanisms. Thus, it is possible that at least some of these “orphan” mutation frequency patterns are tissue or cell-type specific. Mutation patterns, along with clinical, transcriptional and other data have been integrated to improve the classification of tumor subtypes (Hoadley et al. 2014; Kandoth et al. 2013). We have used our Manhattan distances to place all 908 SAMMs (excluding the single ALL cancer genome) in relation to one another to assess the extent to which the correct tissue of origin of a tumor can be inferred from its position relative to all tumor samples, based on the identity of its nearest neighbor.

For example, a breast tumor SAMM would be identified as having the correct tissue of origin if the nearest neighbor were to belong to either of the breast cancer datasets. The same would be true for the three liver cancer datasets as well as the four pancreatic cancer datasets. Samples from the medulloblastoma and pediatric brain cancer datasets were also assigned to the same tissue of origin. The results are reported in Supplemental Table 4, which lists each sample and its dataset, as well as the nearest neighbor sample, its dataset, and the distance between their SAMMs. We were gratified that our procedure was able to identify the correct tissue of origin 89.5 % of the time, a finding which supports the emerging view that cell-type-specific mutational processes are prevalent in cancer biology (Hoadley et al. 2014).

Extending our analysis further, a distance-dependent *k*-nearest neighbor classifier was used to predict the tissue of origin. Along with the single ALL cancer genome, the four samples in the LAML-KR dataset were excluded from this analysis due to an insufficient number of samples. The remaining 904 cancer genomes were placed into 11 Groups, or cancer types, as shown in Supplemental Table 5a. The initial analysis used 6-nearest neighbors (*k* = 6) and the unnormalized probability of belonging to a neighbor’s Group was 0.5 when the Manhattan distance to this neighbor was 0.5 (*D*_0.5_ = 0.5, *β* = 0.25). The value for *D*_0.5_ appeared reasonable since, overall, the average distance to the nearest neighbor SAMM was 0.143 with a standard deviation of 0.065.

Once the probabilities were scaled to a sum of 1.0, each SAMM was assigned to a particular Group if the probability of belonging to that Group was at least 0.5; otherwise the predicted tissue of origin was Undetermined. The results for each of the 11 Groups are shown in Supplemental Table 5b, and the distribution of probabilities across all 11 Groups and the Undetermined Group is shown for each sample in Supplemental Table 6. Overall, 86.9 % of all samples were assigned to the correct tissue of origin from a comparison of those correctly classified and those assigned to the wrong tissue of origin, and 8.8 % of all samples had an Undetermined tissue of origin.

For the 95 breast cancers, 94 were correctly classified; sample DO1001 from the BRCA-UK dataset had a probability of 0.976 of being Undetermined (Supplemental Table 6). 288 of the 303 liver tumors were assigned to the correct tissue of origin; six were assigned to the wrong tissue, and nine were Undetermined. Conversely, none of the 10 prostate samples were assigned the correct tissue of origin; six were assigned to the wrong tissue and four were Undetermined.

To reduce the number of SAMMs with an Undetermined classification, a maximum likelihood procedure was used where each SAMM was assigned to the cancer tissue type with the highest probability, independent of its value. When this was done, only the DO1001 sample from the BRCA-UK dataset had an Undetermined classification (Supplemental Table 5c). Overall, 83.5 % of all samples (754 of the remaining 903 SAMMs) were assigned to the correct tissue of origin. Here, all the prostate SAMMs were assigned to incorrect tissues of origin.

To determine the effect of the classification accuracy on the number of nearest neighbors (*k*), the classifications were performed with *k* varying from three to eight. If the final assignment was made by requiring the scaled probability to be at least 0.5, between 2.5 and 8.8 % of all samples were Undetermined, and between 85.7 and 89.2 % of the remaining SAMMs yielded the correct tissue of origin (Supplemental Table 5d). When the maximum likelihood criterion was used, between 82.4 and 86.0 % of the 903 SAMMs were correctly classified (Supplemental Table 5e), and only DO1001 was Undetermined.

To examine the effect of varying *D*_0.5_ on the classification accuracy, a distance-dependent 6-nearest neighbor classifier was used with *D*_0.5_ varying from 0.4 to 0.7. Requiring the scaled probability to be at least 0.5 (Supplemental Table 5f) caused between 8.3 and 9.2 % of the SAMMs to have an Undetermined tissue of origin and between 86.7 and 87.1 % of the remaining samples to be assigned to the correct tissue of origin. The maximum likelihood procedure yielded an 83.5 % correct classification with DO1001 being Undetermined for all values of *D*_0.5_ (Supplemental Table 5 g).

Overall, these results suggest that the classification accuracy for the tissue of origin is relatively insensitive to the number of nearest neighbors and the value of *D*_0.5_. While the maximum likelihood criterion reduced the number of Undetermined tissues of origin, forcing these outliers into one of the 11 tissue Groups led to a larger number of incorrect classifications, thereby reducing the percentage of SAMMs assigned to the correct tissue of origin.

To compare these results with a standard *k*-nearest neighbor classifier that does not use *D*_0.5_ and *P* (und), the classification of tissue of origin was repeated varying *k* from three to eight. Requiring a scaled probability of at least 0.5 for a classification (Supplemental Table 5h), between 0.1 and 4.2 % of the samples had an Undetermined tissue of origin. The highest level was for 7-nearest neighbors, where for 38 SAMMs their seven neighboring SAMMs were heterogeneous enough to allow no probability to exceed 0.5. For the remaining samples, between 83.3 and 86.0 % were assigned to the correct tissue of origin, with the accuracy generally decreasing as the number of neighbors increased, as expected. When a maximum likelihood criterion was used (Supplemental Table 5i), an Undetermined assignment was not allowed and between 82.5 and 86.1 % of all SAMMs were assigned to the correct tissue of origin. The breakdown by Groups is shown in Supplemental Table 5j and 5k, respectively. It is interesting to note that the DO1001 sample from the BRCA-UK dataset was allocated a correct tissue of origin even though all of the distance-dependent classifications found it to be an outlier.

We believe that integrating our approach with current diagnostic tools could serve to improve tumor classification scores. This would necessitate increasing the number of cancer genomes for each tissue type and including more tissue types in the analysis, but this analysis nevertheless constitutes a promising start. In addition, refinement of the SAMMs representing specific mutation mechanisms and increasing the number of MTMMs might also improve the tissue of origin classification, since maximizing the amount of contributory information should improve the prediction.

## Conclusions

There are several aspects to this investigation which, we believe, set our analysis of cancer genome mutational spectra apart from all other studies to date. First, by analyzing pentamer motifs, we have captured the influence of two nucleotides upstream and downstream of the mutation sites without loss of discriminatory power. This information is stored as a 32 × 12 somatic autosomal mutation matrix (SAMM). Second, we constructed canonical MTMMs representing four of the most common mutational mechanisms, viz., oxidative damage, UV-induced CPD formation, methylation-mediated deamination and APOBEC-induced deamination. For the oxidative damage MTMM, we applied quantum chemical calculations to derive vertical ionization potentials, which were then used to establish mutational patterns for all relevant trinucleotide motifs. Of the 15 sample SAMMs that contained the strongest signature from this mechanism, 13 represented lung cancers. This suggests that loss of an electron is the rate-limiting step in this mutational mechanism. We believe this is a relevant conclusion since it applies the findings accumulated over the past 30 years from the studies of electron transfer reactions on short DNA oligomers to the field of human cancer biology. Thus, the MTMM shown in Supplemental Figure 1a and Supplemental Table 1a may represent the true pattern for oxidative damage. Third, by calculating the Manhattan distance between SAMMs from different samples, we constructed a Sammon map that provides an ‘anatomical’ representation of how cancer tissues are related to each other, and shows sub-clusters containing specific cancer types. Note that this is very different from measuring projections of a sample’s SAMM along mechanistic or any other non-orthogonal signatures, since this procedure will not preserve distance. We believe that preserving the inter-sample distance is critical for achieving high-resolution clustering.

We show that cancer tissue preference exists for each MTMM (lung for oxidative damage, skin for photodamage, pancreatic cancer for ^5m^CpG deamination and breast cancer for APOBEC activity). Most importantly, Manhattan distances were able to achieve 89.5 % accuracy in placing tumor SAMMs of the same tissue type as nearest neighbors, implying that our in-depth analysis of single-base substitution patterns nears the diagnostic power currently attained by clinical and pathological analyses. Thus, our approach may eventually augment current diagnostic procedures by helping to improve tumor classification scores. Nevertheless, the existence of prominent mutational signatures over and above the four mutational processes considered here, and the finding that specific types of tumor were consistently misclassified, highlights the need to further address the mechanisms underlying the origin of mutations in a tissue- or cell-type-specific fashion.

## Electronic supplementary material

Supplementary material 1 (DOCX 4112 kb)

Supplementary material 2 (XLSX 16 kb)

Supplementary material 3 (XLSX 799 kb)

Supplementary material 4 (XLSX 67 kb)

Supplementary material 5 (XLSX 47 kb)
