# Supplementary material for: The somatic autosomal mutation matrix in cancer genomes
Source: Hum Genet. 2015 May 23;134(8):851–64. doi: 10.1007/s00439-015-1566-1 (PMC4495249; doi:10.1007/s00439-015-1566-1)
Supplement: Supplementary file 1 — Supplementary material 1 (DOCX 4112 kb) [file 439_2015_1566_MOESM1_ESM.docx]

**The Somatic Autosomal Mutation Matrix in Cancer Genomes**

Nuri A. Temiz, Duncan E. Donohue, Albino Bacolla, Karen M. Vasquez, David N. Cooper, Uma Mudunuri, Joseph Ivanic, Regina Z. Cer, Ming Yi, Robert M. Stephens, Jack R. Collins, Brian T. Luke

**SUPPLEMENTAL MATERIAL**

**SUPPLEMENTAL RESULTS AND DISCUSSION**

This section describes the results for each dataset individually. A comparison of all 909 samples is presented in the main paper.

*Acute Lymphoblastic Leukemia (ALL, Sanger)*

This dataset contains only one sample, PD4020a, with 7,442 single base substitutions (SBSs) contributing to its somatic autosomal mutation matrix (SAMM). The SAMM heatmap and the top five scaled mutation frequencies (i.e. the five largest elements of the SAMM) are shown in Supplemental Figure 3. As shown in Supplemental Table 3a, up to 28% of all scaled mutation frequencies in the SAMM may be accounted for by the action of an APOBEC enzyme. This cancer genome contains 23 mutation clusters, of which 16 contain a putative APOBEC signature.

*Acute Myeloid Leukemia (LAML-KR, ICGC)*

In this dataset, four cancer genomes contained a sufficient number of SBSs to generate SAMMs. One of these samples (DO49532) is different from the other three, exhibiting an average Manhattan distance (AMD) of 0.495. This is reflected in the dendrogram (Supplemental Figure 2a) and the distance matrix (Supplemental Table 2a). Of the three very similar SAMMs, DO49535 has the smallest AMD to the other two samples (0.062), and was therefore selected as the representative SAMM. The SAMM heatmaps and top five scaled mutation frequencies for DO49532 and DO49535 are shown in Supplemental Figure 4. While all of the five largest elements in both SAMMs are indicative of ^5m^CpG deamination, the scaled frequencies are larger in DO49535. This is also reflected in Supplemental Table 3b, where up to 29.7% of the SAMM elements can be attributed to ^5m^CpG deamination, whereas the maximum contribution from this mutation mechanism is only 10.5% in DO49532.

*Breast Triple Negative Lobular Cancer (BRCA-UK, ICGC)*

This dataset contains 18 cancer genomes with at least 2,000 SBSs. The average linkage clustering dendrogram for these samples is shown in Supplemental Figure 2b. At an AMD of 0.38, there are four clusters; two singleton clusters (DO1016 and DO1076), one that contains seven samples, and one containing the remaining nine samples. DO1010 has the smallest AMD (0.265) to the other samples in the cluster with seven samples, and DO1003 has the smallest AMD to the other samples (0.241) in the cluster with nine sample genomes. Therefore, they were used as representative genomes for these clusters. The heatmap and top five scaled frequencies for the SAMMs of DO1003, DO1010, DO1016, and DO1076 are shown in Supplemental Figure 5. All of the top five mutation frequencies are consistent with ^5m^CpG deamination, but the signal from this mutational mechanism is much larger in DO1010. The maximum contribution from this mechanism (Supplemental Table 3c) is 11.2% in DO1003, and 22.3% in DO1010. The largest maximum contribution in DO1003 is from oxidative damage (14.2%). By contrast, the SAMMs for DO1016 and DO1076 show a maximum contribution from the action of an APOBEC enzyme; 25.9% and 33.1%, respectively. The cancer genome from DO1016 harbors 12 mutation clusters that display the APOBEC signature, while DO1076 contains 117 such mutation clusters.

Breast Cancer (Breast, Sanger)

This dataset contains 77 GWS samples with at least 2,000 somatic autosomal mutations. The dendrogram for these samples is shown in Supplemental Figure 2c, and at an AMD of 0.34 there are 10 clusters. The Sammon map showing the relative positions of these 10 clusters is shown in Supplemental Figure 6. One cluster contains only a single sample (PD4266a), one contains two samples (PD6043a and PD6722a), four clusters contain three samples, one contains four samples, one contains 10 samples, one contains 15 samples, and the largest cluster contains the remaining 33 samples. For the cluster with two samples, the SAMM for PD6722a was examined since it had the larger AMD to the other 76 SAMMs. PD4005a, PD4120a, PD 7404a, and PD4224a were the SAMMs in the clusters with three members that had the smallest AMD within each cluster, and they could therefore be regarded as representative samples. The representative samples for the clusters with four, 10, 15 and 33 members are PD4980a, PD4315a, PD4965a, and PD4952a, respectively, since they had the smallest AMD to the other members of their cluster.

The SAMM heatmaps and top five SAMM elements (scaled mutation frequencies) for PD4266a, PD6722a, PD4005a, PD4120a, PD7404a, PD4224a, PD4980a, PD4315a, PD4965a and PD4952a are shown in Supplemental Figure 7. The SAMM for PD4226a shows that all of the top five mutation frequencies represent C→T transitions within a CpG step, indicative of ^5m^CpG deamination. This mutational mechanism may account for up to 19% of the total SAMM (Supplemental Table 3d). The second largest contribution is from the action of an APOBEC enzyme, accounting for up to 13.4% of the total SAMM. It is interesting to note that PD4266a contains no mutation clusters, although the absence of mutation clusters does not exclude APOBEC-mediated mutation ([Roberts and Gordenin 2014](#_ENREF_3)).

The SAMM for PD6722a shows a much weaker ^5m^CpG deamination signature, and this mechanism contributes at most 7.4% to the total SAMM. The largest maximum contribution is from the action of an APOBEC enzyme, contributing up to 21.5% of the total SAMM. This cancer genome contains two mutation clusters with an APOBEC signature. In PD4005a, only two of the top five mutation frequencies are consistent with ^5m^CpG deamination, and this mechanism contributes at most 12.4% to the total SAMM. This cancer genome has the largest maximum contribution from oxidative damage (OxD) of the 10 cancer genomes examined in Supplemental Figure 7, but the largest maximum contribution is from APOBEC-mediated mutations (up to 15.4% of the total SAMM). Although this cancer genome contains four mutation clusters, none have an APOBEC signature.

The SAMM for PD4120a is dominated by mutations from an APOBEC enzyme, and this mechanism may contribute up to 34.5% of the total SAMM. It contains 112 mutation clusters that exhibit an APOBEC signature. The other two members of this cluster, PD4072a and PD4607a, contain four and 10 mutation clusters with APOBEC signatures, respectively, and the maximum contributions from this mechanism are 32.3% and 33.7%, respectively.

^5m^CpG deamination has the largest maximum contribution to the PD7404a SAMM (up to 18.3%) and accounts for all of the top five mutation frequencies, while the maximum contribution from OxD is 12.8%. Although the maximum contribution from APOBEC-mediated mutations is only 7.2%, this cancer genome contains 17 mutation clusters with an APOBEC signature. None of the top five mutation frequencies in the SAMM of PD4224a is consistent with ^5m^CpG deamination, and this mechanism represents at most 7.2% of the total SAMM. Up to 28.7% of the SAMM can be associated with the action of an APOBEC enzyme and this cancer genome contains six mutation clusters with an APOBEC signature.

The SAMMs for PD4980a, PD4315a, and PD4965a are all dominated by ^5m^CpG deamination, which represents the top five mutation frequencies in each cancer genome. This mechanism contributes up to 19.6, 24.8, and 29.9% of the total SAMMs, respectively. The maximum contribution from OxD is also quite large, representing 12.2, 13.3, and 10.9% of their SAMMs, respectively. PD4315a has a relatively large contribution from the action of an APOBEC enzyme, accounting for up to 12.1% of the total SAMM. This cancer genome contains one mutation cluster with an APOBEC signature, while PD4980a and PD4965a do not. Whereas the SAMM for PD4952a has all five of its top mutation frequencies consistent with ^5m^CpG deamination, the largest maximum contribution comes from OxD. These mechanisms may account for 14.1% and 17.2% of the total SAMM, respectively. Although the maximum contribution from APOBEC-mediated mutations is relatively small (6.9%), the cancer genome for PD4952a contains three mutation clusters with an APOBEC signature.

*Esophageal Adenocarcinoma (ESAD-UK, ICGC)*

The ESAD-UK dataset contained 16 cancer genomes with enough SBSs to generate SAMMs. The average linkage dendrogram for these SAMMs is shown in Supplemental Figure 2d. At an AMD of 0.40, there are four clusters; two contain seven SAMMs and two singleton clusters (DO10843 and DO 10853). For the first seven-member cluster, DO10842 has the smallest AMD to the other sex members and will therefore be the representative SAMM for this cluster. By the same argument, DO10840 is the representative SAMM of the second seven-member cluster. The SAMM heatmaps and five largest elements for DO10842, DO10840, DO10843, and DO10853 are shown in Supplemental Figure 8.

For these four SAMMs, DO10842 has the smallest maximum contribution from ^5m^CpG deamination (15.4%), although it is larger than for any of the other three mechanisms examined (Supplemental Table 3e). This mechanism is consistent with the third and fifth largest elements in the SAMM. The other three largest mutation frequencies represent A→C (T→G) transversions. The SAMMs for DO10840, DO10843, and DO10853 all have the top five elements consistent with ^5m^CpG deamination and the maximum contributions from this mechanism account for up to 25.5, 27.5 and 22.4% of the SAMMs, respectively. DO10853 also has a large maximum contribution from OxD, representing up to 19.4% of the total SAMM. Whilst DO10843 has the largest maximum contribution from APOBEC-mediated mutations (6.5%), DO10840 is the only cancer genome with a mutation cluster containing an APOBEC signature. Four other members of the cluster containing DO10840 (DO10851, DO10852, DO10856, and DO10857) also display mutation clusters with an APOBEC signature.

*Liver Cancer NCC (LINC-JP, ICGC)*

A total of 31 cancer genomes from this dataset contain sufficient SBSs to construct SAMMs. The dendrogram for these SAMMs is shown in Supplemental Figure 2e, and at an AMD of 0.28 there are five clusters. One contains 19 SAMMs, one contains six SAMMs, one contains four SAMMs, and there are two singleton clusters (DO45041 and DO45047). The SAMM for DO45031 has the smallest AMD to the other 18 members of the largest cluster and it will be used as the representative SAMM. Using the same criteria, DO45069 and DO45061 are the representative SAMMs for the clusters with six and four members, respectively. The SAMM heatmaps and top five elements for DO45031, DO45069, DO45061, DO45041, and DO45047 are shown in Supplemental Figure 9.

The top five mutation frequencies in the SAMM for DO45031 and DO45069 represent the deamination of ^5m^CpG. The maximum contribution of this mechanism to the total SAMM is 8.5% for both cancer genomes (Supplemental Table 3f). In both cases, the maximum contribution of OxD is larger; 12.4% and 12.8%, respectively, and neither SAMM haa a mutation frequency that dominates. Conversely, the SAMMs for DO45061 and DO45041 show a much stronger signature from ^5m^CpG deamination. This mechanism contributes up to 15.4% and 19.5% to the total SAMMs, respectively, and again represents all of the top five mutation frequencies. For DO45047, the largest maximum contribution to its SAMM comes from OxD (14.1%), while the MTMM representing ^5m^CpG deamination has a maximum contribution of 11.6%. The two highest mutation frequencies in this SAMM are G→C (C→G) and A→C (T→G) transversions, and the next three are indicative of ^5m^CpG deamination.

The maximum contribution from APOBEC-mediated mutations is not large for any of the five SAMMs, although DO45061 contains a single mutation cluster with an APOBEC signature. In addition, two members of the largest cluster, DO45043 and DO45077, have one and two mutation clusters with an APOBEC signature, respectively, but the maximum contributions of the mutational mechanism are only 3.7% and 3.1%, respectively.

*Liver Cancer RIKEN (LIRI-JP, ICGC)*

This dataset contains 188 cancer genomes with at least 2,000 SBSs. The average linkage clustering dendrogram for the resulting SAMMs is shown in Supplemental Figure 2f. At an AMD of 0.33, there are seven clusters; two singleton clusters (DO45157 and DO45301), two clusters that contain two members, one cluster with three members, one cluster with 10 members, and a large cluster containing 169 members. To show the relative orientation of the 188 SAMMs, a Sammon map is shown in Supplemental Figure 10, color coded by each of the seven clusters. For one of the clusters with only two members, DO45293 has the largest AMD to the other SAMMs in non-singleton clusters and will therefore be examined. Using the same criterion, DO48682 will be used to represent the other cluster containing only two members. For clusters with three or more members, the representative SAMM will be the member with the smallest AMD to the other members of its cluster. DO45211, DO45305, and DO23521 therefore are representative SAMMs for the clusters containing three, 10 and 169 members, respectively.

The SAMM heatmaps and top five elements (mutation frequencies) for DO45157, DO45301, DO45293, DO48682, DO45211, DO45305, and DO23521 are shown in Supplemental Figure 11. The heatmap of the SAMM for DO45157 reveals no presominant mutation frequencies. Three of the five top frequencies are consistent with ^5m^CpG deamination, and the maximum contribution of this mechanism to the SAMM is only 6.3% (Supplemental Table 3g). The first and third largest frequencies are A→C (T→G) and G→T (C→A) transversions, both of which are contained in the OxD SAMM (Supplemental Table 1a, Supplemental Figure 1a). The maximum contribution of this mechanism to the SAMM of DO45157 is 9.7%. The SAMM for DO45301 shows a stronger signature from ^5m^CpG deamination, which exhibits a maximum contribution of 12.3%. All of the top five mutation frequencies are consistent with this mechanism. The maximum contribution from any of the four mechanisms considered is from OxD (15.3%).

The SAMM for DO45293 shows a weak signature from ^5m^CpG deamination, although all of the top five frequencies are consistent with this mechanism. The maximum contribution from OxD is slightly larger than for ^5m^CpG deamination; 10.9% and 9.8%, respectively. Although the maximum contribution from APOBEC-mediated mutation is only 3.3%, this cancer genome contains a mutation cluster with an APOBEC signature. The SAMM for DO48682 shows a larger maximum contribution from ^5m^CpG deamination (24.4%) and this mechanism accounts for all of the top five mutation frequencies. Although the maximum impact from the action of an APOBEC enzyme is quite small (3.6%), this cancer genome also contains a mutation cluster with an APOBEC signature.

DO45211, the representative SAMM from the cluster with three members, has a very strong ^5m^CpG deamination signature, and this mechanism has a maximum contribution of 35.8% of the total SAMM. All of the top five mutation frequencies are consistent with this mechanism. The SAMM for DO45305, the representative of the cluster with 10 members, also contains a strong ^5m^CpG deamination signature. This mechanism accounts for all of the top five mutation frequencies and has a maximum contribution of 23.1% to the total SAMM. It also has the second highest maximum contribution from OxD of the seven SAMMs examined, representing up to 13.9% of the total SAMM. Finally, the cluster with 169 members is represented by the SAMM from DO23521. This SAMM has the second smallest maximum contribution from ^5m^CpG deamination (9.2%) of the seven SAMMs examined in Supplemental Figure 11, but the mechanism is consistent with all of the top five mutation frequencies. The largest maximum contribution to this SAMM comes from OxD (15.7%).

*Liver Cancer (Liver, Sanger)*

This dataset contains 84 GWS cancer genomes with sufficient SBSs to generate SAMMs. The average linkage clustering dendrogram is shown in Supplemental Figure 2g. At an AMD of 0.35, there are six clusters; four are singleton clusters (HX13T, HX16T, RK126_C01, and RK138_C01), one cluster contains four SAMMs and the other contains the remaining 76. The orientation of these clusters is shown in the Sammon map in Supplemental Figure 12. The representative SAMMs of the non-singleton clusters are from HX23T and RK051_C01 since they have the smallest AMD to the other SAMMs in their cluster.

The SAMM heatmaps and five largest elements for HX13T, HX16T, RK126_C01, RK138_C01, HX23T, and RK051_C01 are shown in Supplemental Figure 13. For the four singleton clusters, the maximum contribution from ^5m^CpG deamination decreases in the order RK138_C01, HX13T, HX16T, and RK126_C01, and accounts for up to 35.5, 18.6, 12.6 and 6.5% of the total SAMM, respectively. In the SAMMs for RK138_C01, HX13T, and HX16T, all of the top five mutation frequencies are consistent with this mechanism. The highest frequency in RK126_C01 is an AAG→ACG (CTT→CGT) transversion, possibly suggesting OxD (Supplemental Table 1a). In the SAMMs for both HX16T and RK126_C01, the maximum contribution from OxD is larger than for the other three mutational mechanisms (20.2% and 14.9%, respectively). Even although the maximum contribution from APOBEC-mediated mutations is only 3.9% in the SAMM for RK126_C01, this cancer genome contains one mutation cluster with an APOBEC signature.

The SAMM for HX23T, which is representative of the cluster containing four members, also shows a significant ^5m^CpG deamination pattern. The maximum contribution from this mutational mechanism is up to 15.2% of the total SAMM, and all of the top five mutation frequencies are consistent with this mechanism. Up to 11.3% of the total SAMM can be attributed to OxD. Although APOBEC-mediated mutations only contribute a maximum of 2.5% of the total SAMM, this cancer genome contains a mutation cluster with an APOBEC signature. The 76-member cluster is represented by the SAMM from RK051_C01 and its heatmap shows a diffuse pattern with no real hotspots. Although ^5m^CpG deamination may account for all of the top five mutation frequencies, the values of these frequencies are quite small. The maximum contribution from this mechanism is only 9.4%. The largest maximum contribution comes from OxD (16.4%). This cancer genome contains one cluster with an APOBEC signature, even though this mechanism has a maximum contribution of only 2.7% to the total SAMM.

*Lung Adenocarcinoma (Lung_Adeno, Sanger)*

A total of 23 cancer genomes from this dataset have at least 2,000 autosomal somatic SBSs. The dendrogram from the average linkage clustering of their SAMMs is shown in Supplemental Figure 2h. At an AMD of 0.34, there are four clusters; a singleton cluster (LUAD-S01345), two clusters with two members each, and a cluster with the remaining 18 SAMMs. For the clusters with only two members, we examined the SAMM with the largest AMD to the other 22 SAMMs. LU-A08-43 has a larger AMD than LUAD-QY22Z (0.517 versus 0.474), and LUAD-S01341 has a larger AMD than LUAD-E1014 (0.744 versus 0.594), so the SAMMs for LU-A08-43 and LUAD-S01341 were examined. For the cluster with 18 SAMMs, the SAMM with the smallest AMD to the other 17 members (LUAD-FH5PJ) was selected and taken as the representative SAMM.

The heatmaps and five largest mutation frequencies in the SAMMs for LUAD-S01345, LU-A08-43, LUAD-S01341, and LUAD-FH5PJ are presented in Supplemental Figure 14. The SAMM for LUAD-S01345 contains a weak overall signature for ^5m^CpG deamination. This mutational mechanism accounts for the second largest mutation frequency and may contribute up to 8.3% of the total SAMM. The largest maximum contribution is made by APOBEC-mediated mutations which may account for up to 27.2% of the total SAMM. This mutational mechanism (Supplemental Table 1d, Supplemental Figure 1d) is consistent with the other four highest mutation frequencies. Overall, this cancer genome contains 47 mutation clusters, of which 25 contain an APOBEC signature.

In the SAMM for LU-A08-43, all of the top five mutation frequencies may be attributed to ^5m^CpG deamination, and this mechanism has a maximum contribution of 10.3% to its total SAMM. OxD has a maximum contribution of 19.6% to the SAMM, and the maximum contribution from the action of an APOBEC enzyme is 8.0%. This cancer genome contains two mutation clusters, one of which contains an APOBEC signature. The SAMM for LUAD-S01341 shows a strong ^5m^CpG deamination signature. This mechanism accounts for all of the top five mutation frequencies and its maximum contribution is 22.8% of the total SAMM. The maximum contribution from APOBEC-mediated mutations is 13.4% of the total SAMM although none of the five mutation clusters contain an APOBEC signature. Again, the absence of mutation clusters does not exclude APOBEC-mediated mutation ([Roberts and Gordenin 2014](#_ENREF_3)).

The SAMM for LUAD-FH5PJ is very different in that all of the top five mutation frequencies are consistent with OxD (Supplemental Table 1a, Supplemental Figure 1a). This mechanism has a maximum contribution of 29.3% to the overall SAMM. Although the maximum contribution from the action of an APOBEC enzyme is only 2.9%, two of the seven mutation clusters in the cancer genome contain APOBEC signatures.

*Malignant Lymphoma DKFZ (MALY-DE, ICGC)*

This dataset contains 37 cancer genomes that were used to construct SAMMs. The dendrogram obtained from the average linkage clustering of these SAMMs is shown in Supplemental Figure 2i. At an AMD of 0.35, there are five clusters; three singleton clusters (DO27771, DO27777, and DO27779), one cluster that contains the SAMMs of DO27813 and DO27827, and one cluster comprising the remaining 32 SAMMs. The AMD of DO27813 to the other 36 SAMMs (0.430) is larger than the AMD of DO27827 (0.400), so the SAMM of DO27813 was examined. The SAMM for DO27805 had the smallest AMD to the other 31 SAMMs in the largest cluster (0.229) and it was therefore taken as the representative SAMM for this cluster. The heatmaps and five largest elements of the SAMMs for DO27771, DO27777, DO27779, DO27813, and DO27805 are shown in Supplemental Figure 15.

The SAMM for DO27771 does not contain a strong signature from any of the four mutation mechanisms; the largest maximum contribution to the SAMM is from OxD (7.0%). The maximum contribution from the action of an APOBEC enzyme is only 0.6%, and none of the 10 mutation clusters in this cancer genome contain an APOBEC signature. The third and fifth largest mutation frequencies are consistent with ^5m^CpG deamination, although this mechanism only has a maximum contribution of 6.8% to the total SAMM. The other three top mutation frequencies are suggestive of an OxD mechanism.

DO27777 generates a SAMM that has a stronger signature from ^5m^CpG deamination. This mechanism is consistent with all of the top five mutation frequencies and has a maximum contribution of 13.2% to the overall SAMM. The maximum contribution from APOBEC-mediated mutation is only 6.6%, but this cancer genome contains four mutation clusters, all with an APOBEC signature. The largest maximum contribution of a mutational mechanism is for OxD (17.4%).

The SAMM for DO27779 contains a slightly weaker ^5m^CpG deamination, accounting for up to 12.6% of the total SAMM. This mechanism has the largest maximum impact of the four mutational mechanisms considered and may account for four of the top five mutation frequencies. The largest mutation frequency is for GAT→AAT (ATC→ATT) transitions, which may indicate OxD, photo damage via CPD formation, and/or the action of an APOBEC enzyme. Although the maximum contribution of APOBEC-mediated mutations is only 5.8%, six of the 69 mutation clusters contained within this cancer genome harbor an APOBEC signature.

DO27813 produces a SAMM with a definite ^5m^CpG deamination signature contributed by all of the top five mutation frequencies. This mechanism has a maximum contribution of 12.0% to the total SAMM. The largest maximum impact is from OxD (17.5%), and this mechanism may also be responsible for the top five mutation frequencies. Although the maximum contribution from APOBEC-mediated mutation is less than 1% (0.082%), both of the mutation clusters present in this cancer genome contain an APOBEC signature.

The SAMM for DO27805 contains the strongest signature from ^5m^CpG deamination, and this mechanism may be responsible for the top five mutation frequencies. The maximum contribution from this mutational mechanism (22.9%) is much larger than from OxD (9.2%). The maximum contribution from the action of an APOBEC enzyme is only 2.2% and none of the seven mutation clusters present in this cancer genome contains an APOBEC signature.

*Melanoma (Melanoma, (*[*Berger et al. 2012*](#_ENREF_1)*))*

This dataset contains 25 GWS cancer genomes that have sufficient SBSs to generate SAMMs. The dendrogram obtained from the average linkage clustering of these SAMMs is shown in Supplemental Figure 2j. At an AMD of 0.37 there are four clusters, one singleton cluster (ME015), a cluster with two members (ME009 and ME032), a cluster with three members (ME007, ME021, ME048), and a cluster containing the other 19 SAMMs. For the cluster with two SAMMs, the AMD between ME009 and the other 24 SAMMs is 0.702, whereas for ME032 it is 0.671. Since the AMD was larger for ME009, this sample was examined. For the clusters with three and 19 SAMMs, the SAMMs with the smallest AMDs to the other SAMMs in their respective cluster were used as the representative SAMMs; these were ME021 and ME020, respectively. The SAMM heatmaps and five largest elements for ME015, ME009, ME021, and ME020 are shown in Supplemental Figure 16.

The SAMM of ME015 is dominated by a ^5m^CpG deamination signature. This mechanism is consistent with all of the top five mutation frequencies and has a maximum contribution of 17.0% to the total SAMM (Supplemental Table 3k). OxD and photo damage via CPD formation have maximum contributions of 9.1% and 7.8%, respectively. Although the maximum contribution from APOBEC-mediated mutation is only 4.0%, this cancer genome contains 13 mutation clusters, five of which exhibit an APOBEC signature.

The SAMM of ME009 contains a much weaker ^5m^CpG deamination signature, and its maximum contribution to the overall SAMM is 4.5%. Only the fourth largest mutation frequency is consistent with this mechanism, whilst all of the top five mutation frequencies are consistent with OxD and photo damage via CPD formation. The maximum contributions of these two mutational mechanisms are 0.5% and 1.1%, respectively. The second and fifth largest mutation frequencies are also consistent with the action of an APOBEC enzyme, and although the maximum contribution of this mechanism to the total SAMM is only 0.2%, this cancer genome contains 310 mutation clusters, with seven harboring an APOBEC signature.

ME021 generates a SAMM that also has a large ^5m^CpG deamination signature. This mutational mechanism may account for up to 17.0% of the total SAMM, and three of the top five mutation frequencies are consistent with this mechanism. However, these three mutation patterns overlap with the photo damage signatures, whereas all of the top five mutation frequencies are present in the OxD MTMM. The maximum contributions of photo damage and OxD to the total SAMM are 5.7% and 3.5%, respectively. The maximum contribution of APOBEC-mediated mutations is 1.6%; however, we note that although this cancer genome contains 60 mutation clusters, none display an APOBEC signature.

In the SAMM of ME020, the largest frequency is for the mutation CGA→CAA (TCG→TTG). This mutation is consistent with ^5m^CpG deamination, photo damage via CPD formation, and OxD. The second largest mutation frequency in this SAMM is consistent with photo damage and OxD; the third and fifth largest mutation frequencies are consistent with OxD, photo damage, and the action of an APOBEC enzyme; the fourth largest mutation frequency is consistent with OxD and ^5m^CpG deamination. The maximum contribution of OxD, photo damage via CPD formation, ^5m^CpG deamination, and APOBEC-mediated mutations are 2.4, 3.3, 6.0 and 0.9%. Alhough the maximum contribution from the action of an APOBEC enzyme is quite small; this cancer genome contains 68 mutation clusters, five of which exhibit an APOBEC signature.

*Ovarian Cancer QCMG (OV-AU, ICGC)*

This dataset contains 89 cancer genomes with sufficient SBSs to generate SAMMs. The average linkage clustering dendrogram for these SAMMs is shown in Supplemental Figure 2k. At an AMD of 0.30, there are six clusters; three singleton clusters (DO46330, DO46588, and DO46591), one cluster that contains 12 SAMMs, one cluster that contains 18 SAMMs, and one cluster containing the remaining 56 SAMMs. The Sammon map showing the orientation of these six clusters is shown in Supplemental Figure 17. The representative SAMM for each non-singleton cluster is selected as the SAMM with the smallest AMD to other members of its cluster. For the clusters with 12, 18 and 56 SAMMs, the representative SAMM is DO46396, DO46356, and DO46473, respectively. The SAMM heatmaps and top five mutation frequencies for DO46330, DO46588, DO46591, DO46396, DO46356, and DO46473 are shown in Supplemental Figure 18.

The SAMM for DO46330 contains a definite signature from ^5m^CpG deamination, and all of the top five mutation frequencies are consistent with this mechanism. The maximum contribution to the total SAMM from this mechanism is 15.4%. The top five mutation frequencies could also be due to OxD, a mechanism with a maximum contribution of 9.9% to the total SAMM. The second largest maximum contribution comes from APOBEC-mediated mutation and five of the seven mutation clusters in this cancer genome have an APOBEC signature. This mechanism could contribute to the fifth largest mutation frequency. Photo damage via CPD formation is consistent with the third largest mutation frequency, and this mechanism has a maximum contribution of 4.1%.

DO46588 generates a SAMM where the top five mutation frequencies are consistent with ^5m^CpG deamination and OxD. The maximum contributions from these mechanisms to the total SAMM are 20.5 and 17.3%, respectively. The action of an APOBEC enzyme has a maximum contribution of only 2.1%, but this genome contains six mutation clusters and three of them have an APOBEC signature. The top five mutation frequencies in the SAMM for DO46591 are also consistent with ^5m^CpG deamination and OxD, and the maximum contributions of these mechanisms to the total SAMM are 10.5 and 5.2%, respectively. The maximum contribution of APOBEC-mediated mutation is 2.7% and one of the five mutation clusters present in this cancer genome contains an APOBEC signature.

The SAMM for DO46396 contains a very strong ^5m^CpG deamination signature, which accounts for a maximum contribution of 29.9% to the total SAMM. These top frequencies are also consistent with OxD, which has a maximum contribution of 9.0% to the total SAMM. The second highest mutation frequency is also consistent with the action of an APOBEC enzyme. This mechanism has a maximum contribution of 3.0%, and four of the 11 mutation clusters present in this cancer genome contain an APOBEC signature. Similarly, the SAMM for DO46356 also contains a strong ^5m^CpG deamination signature, and all of the top five mutation frequencies are consistent either with this mechanism or OxD. The maximum contribution from these mechanisms to the total SAMM is 26.9% and 9.6% respectively. The second highest mutation frequency is also consistent with APOBEC-mediated mutations. The maximum contribution from this mechanism is 3.2% and two of the five mutation clusters contain an APOBEC signature.

DO46473 produces a SAMM where the top five mutation frequencies are only consistent with OxD and ^5m^CpG deamination. The maximum contributions from these mechanisms are 9.6% and 26.9%, respectively. The maximum contribution from photo damage via CPD formation is 6.5%, but none of the top five mutation frequencies are consistent with this mechanism. APOBEC-mediated mutations have a maximum contribution of 3.2%, but this cancer genome does not contain any mutation clusters.

*Pancreatic Cancer OICR (PACA-CA, ICGC)*

There are 45 cancer genomes in this dataset with at least 2,000 somatic autosomal SBSs. The average linkage clustering dendrogram for these SAMMs is shown in Supplemental Figure 2l. At an AMD of 0.28, there are four clusters; two singleton clusters (DO35442 and DO49418), a cluster containing three SAMMs, and a cluster containing the remaining 40 SAMMs. The orientation of these SAMMs, color coded by cluster, is shown in Supplemental Figure 19. The SAMM for DO49419 and DO49430 are the representative SAMMs for the clusters with three and 40 members, respectively. The heatmaps and top five elements for the SAMMs of DO35442, DO49418, DO49419, and DO49430 are shown in Supplemental Figure 20.

All four of these SAMMs are quite similar in that they show a strong signature from ^5m^CpG deamination. The maximum contribution from this mechanism to the total SAMMs are 31.5, 30.8, 20.5 and 40.2%, respectively (Supplemental Table 3m), and is consistent with all of the top five mutation frequencies in each SAMM. All of these top mutations may also be a result of OxD, for which the maximum contributions are 0.8, 7.6, 12.8 and 7.2%, respectively.

Although the maximum contribution from APOBEC-mediated mutations is only 0.1% in the SAMM of DO35442, this cancer genome contains three mutation clusters, two of which contain an APOBEC signature. The maximum contribution of this mechanism to the SAMM of DO49418 is 3.0%, but this cancer genome only contains a single mutation cluster which does not display an APOBEC signature. For DO49419, the maximum contribution of this mechanism to its SAMM is 4.9% and this cancer genome contains a single mutation cluster, and it has an APOBEC signature. Finally, the cancer genome for DO49430 contains 10 mutation clusters and three of them have an APOBEC signature. The maximum contribution of this mechanism to its overall SAMM is 2.6%.

*Pancreatic Cancer QCMG (PACA-AU, ICGC)*

This dataset contains 137 cancer genomes with sufficient SBSs to generate SAMMs. The average linkage clustering dendrogram for these SAMMs is shown in Supplemental Figure 2m. At an AMD of 0.40, there are seven clusters; four singleton clusters (DO32819, DO32976, DO33043, and DO33056), one cluster containing five SAMMs, one cluster with six SAMMs, and one cluster with the remaining 122 SAMMs. The relative orientation of the 137 SAMMs is shown as Sammon map in Supplemental Figure 21. The SAMMs for DO34128, DO49087, and DO49130 are representative of the clusters with five, six, and 122 members, respectively, since they have the smallest AMD to the other SAMMs in their cluster. The heatmaps and top five mutation frequencies in the SAMMs for DO32819, DO32976, DO33043, DO33056, DO34128, DO49087 and DO49130 are shown in Supplemental Figure 22.

The top five mutation frequencies in SAMM for DO32819 are consistent with OxD, whereas only one is consistent with ^5m^CpG deamination. The maximum contribution for these mechanisms is 10.5% and 13.2%, respectively (Supplemental Table 3n). The maximum contributions from photo damage via CPD formation and APOBEC-mediated mutations is 3.2% and 1.1%. This cancer genome contains five mutation clusters, but none exhibit an APOBEC signature.

The SAMM for DO32976 contains a strong ^5m^CpG deamination signature and the maximum contribution of this mechanism to the overall SAMM is 27.6%. All of the top five mutation frequencies are consistent with this mechanism and OxD, although the maximum contribution of the latter to the total SAMM is only 6.3%, the smallest contribution from this mechanism in any of the seven SAMMs examined. The maximum contribution from the action of an APOBEC enzyme is 2.0% and two of the three mutation clusters present in this cancer genome contain an APOBEC signature.

DO33043 produces a SAMM that has a slightly weaker signature from ^5m^CpG deamination, and this mechanism has a maximum contribution of 16.2%. Only three of the top five mutation frequencies are consistent with either this mechanism or OxD, which has a maximum contribution of 7.3%. The top two mutation frequencies are for GGA→AGA (TCC→TCT) and GGG→GAG (CCC→CTC), both of which appear in the OxD and photo damage MTMMs. The maximum contribution from photo damage is 7.8%, which is the largest for this mechanism in any of the seven SAMMs examined. The maximum contribution from APOBEC-mediated mutations is 2.2%. This cancer genome contains 16 mutation clusters, only one of which has an APOBEC signature.

In the SAMM for DO33056, four of the top five mutation frequencies are consistent with ^5m^CpG deamination and OxD. There is pronounced ^5m^CpG deamination with a maximum contribution of 25.2%, meaning that about one-quarter of the mutation frequencies in this SAMM could be attributable to this mechanism (Supplemental Table 1c, Supplemental Figure 1c). The maximum contribution from OxD is 7.1%. The second largest mutation frequency in this SAMM is GGT→GGG (ACC→CCC), which is consistent with OxD. The maximum contribution to the SAMM from the action of an APOBEC enzyme is 2.7% and one of the three mutation clusters present in this cancer genome contains an APOBEC signature.

The SAMM for DO34128, the representative of the five member cluster, contains a weaker ^5m^CpG deamination signature, but is consistent with all of the top five mutation frequencies. These mutations are also consistent with OxD, and the maximum contributions from ^5m^CpG deamination and OxD are 16.5% and 13.1%, respectively. This is the largest maximum contribution from OxD in any of the seven SAMMs examined. Although the maximum contribution from APOBEC-mediated mutations is 6.9%, the largest value for any of the seven SAMMs, this cancer genome contains three mutation clusters, none of which have an APOBEC signature.

DO49087 is the representative of the six member cluster and its SAMM is characterized by a strong ^5m^CpG deamination signature. All of the top five mutation frequencies are consistent with this mechanism and its maximum contribution to the total SAMM is 23.1%. These mutations are also consistent with OxD, but its maximum contribution is only 7.5% since many of the mutations produced by this mechanism have low frequencies in the SAMM (Supplemental Table 1a, Supplemental Figure 1a). Although the maximum contribution from APOBEC-mediated mutations is only 1.4%, one of the three mutation clusters in this cancer genome contains an APOBEC signature.

The SAMM for DO49130, the representative of the 122 member cluster, contains a very strong ^5m^CpG deamination signature. All of the top five mutation frequencies are consistent with this mechanism and its maximum contribution to the overall SAMM is 36.1%. The maximum contributions from OxD, photo damage via CPD formation, and the action of an APOBEC enzyme are 8.3, 3.9 and 2.7%, respectively. This cancer genome contains five mutation clusters and an APOBEC signature is present in one of them.

*Pancreatic Cancer Endocrine Neoplasms QCMG (PAEN-AU, ICGC)*

This dataset has 12 cancer genomes with sufficient SBSs to produce SAMMs. After average linkage clustering, the 12 SAMMs produce the dendrogram shown in Supplemental Figure 2n. At an AMD of 0.36, there are four clusters; a singleton cluster (DO46779), a cluster with two SAMMs (DO46749 and DO46783), a cluster containing three SAMMs, and one containing the remaining six SAMMs. Since the AMDs from DO46749 and DO46783 to the other 11 SAMMs were 0.694 and 0.518 (Supplemental Table 2n), the SAMM for DO46749 was examined from this cluster. By comparison, the AMD of DO46779 to the other 11 SAMMs is much larger (1.227). DO46785 and DO48581 have the smallest AMD to the other members of the clusters with three and six members, respectively, so they were used as the representative SAMMs. The SAMM heatmaps and top five mutation frequencies for DO46779, DO46749, DO46785 and DO48581 are shown in Supplemental Figure 23.

The SAMM for DO46779 contains a weak signature from ^5m^CpG deamination (Supplemental Figure 1c, Supplemental Table 1c), and this mechanism has a maximum contribution of 5.8% to the total SAMM (Supplemental Table 3o). The largest maximum contribution to this SAMM is from OxD (16.9%). All of the top five mutation frequencies are G→T (C→A) transversions, and they are all consistent with this mutational mechanism.

DO46749 produces a SAMM with the largest maximum contribution from ^5m^CpG deamination of any of the 12 SAMMs in this dataset (38.7%). All of the top five mutation frequencies are consistent with this mechanism. By contrast, the maximum contribution from OxD, which is also consistent with these top mutation frequencies, is only 2.7%.

The top five mutation frequencies in the SAMMs for DO46785 and DO48581 are C→T (G→A) transitions within a CpG step, consistent with ^5m^CpG deamination. The maximum contributions of this mechanism to the SAMMs are 12.2% and 11.3%, respectively. These top mutation frequencies are also consistent with OxD, and the maximum contributions from this mechanism are 6.3% and 14.8%, respectively.

In all four SAMMs, the maximum contribution from APOBEC-mediated mutations is 2.4%. In the SAMM for DO46783, the other member of the cluster with two SAMMs, the maximum contribution from this mechanism is 3.2% and one of the three mutation clusters in this cancer genome contains an APOBEC signature. This is the only cancer genome in this dataset with a putative APOBEC cluster.

*Pancreatic Cancer (Pancreas, Sanger)*

This dataset contains 14 cancer genomes with at least 2,000 somatic autosomal SBSs. The dendrogram for these 14 SAMMs is shown in Supplemental Figure 2o. At an AMD of 0.25, there are four clusters; two singleton clusters (APGI_2137 and APGI_2157), a cluster containing the SAMMs for APGI_2057 and APGI_2179, and a cluster containing the remaining 10 SAMMs. The AMD between APGI_2057 and APGI_2179 and the remaining 13 SAMMs is 0.578 and 0.560, respectively (Supplemental Table 2o). Since APGI_2057 has the larger AMD, it was selected from this cluster. APGI_2353 had the smallest AMD to the other SAMMs in the cluster with 10 members, and it was selected as the representative SAMM. The heatmaps and top five mutation frequencies in the SAMMs for APGI_2137, APGI_2157, APGI_2057, and APGI_2353 are shown in Supplemental Figure 24.

In all four SAMMs, the top five mutation frequencies are C→T (G→A) transitions within a CpG step, suggesting ^5m^CpG deamination. The maximum contribution from this mechanism to the SAMMs is 21.0, 33.2, 12.6 and 32.4%, respectively (Supplemental Table 3p). These mutations are also consistent with OxD, and this mechanism has a maximum contribution of 16.0, 6.0, 13.8 and 5.9%, respectively, to the total SAMM.

APGI_2137 produces a SAMM with a maximum contribution from APOBEC-mediated mutation of 7.2%, and two of the eight mutation clusters in this cancer genome contain an APOBEC signature. The maximum contribution of this mechanism to the SAMM of APGI_2157 is 12.9% and an APOBEC signature is present in six of the seven mutation clusters contained in this cancer genome. In the SAMM for APGI_2057, the maximum contribution from this mechanism is 5.5% and three of the five mutation clusters in the genome contain an APOBEC signature. By contrast, the maximum contribution from the action of an APOBEC enzyme is 2.6% in the SAMM of APGI_2353, and an APOBEC signature is not present in any of the four mutation clusters present in this cancer genome.

*Medulloblastoma (Medulloblastoma, Sanger)*

Eleven of the cancer genomes in this dataset yielded SAMMs and the average linkage clustering dendrogram for these SAMMs is shown in Supplemental Figure 2p. At an AMD of 0.28, there are three clusters; a singleton cluster (MD110), a cluster containing four SAMMs, and a cluster containing six SAMMs. MB34 and MB101 have the smallest AMD to other SAMMs in their clusters of four and six SAMMs (Supplemental Table 2p), respectively, and are the representative SAMMs. The heatmaps and top five mutation frequencies in the SAMMs for MB110, MB34, and MB101 are shown in Supplemental Figure 25.

All three SAMMs contain a definite signature from ^5m^CpG deamination, which increases in the order MB110, MB34 and MB101. All of the top five mutation frequencies are consistent with this mechanism and its maximum contribution to the SAMMs is 13.1, 29.7 and 32.9%, respectively (Supplemental Table 3q). It is interesting to note that the SAMM for MB34 contains no contribution from OxD, meaning that some of the mutations expected by this mechanism (Supplemental Table 1a, Supplemental Figure 1a) are missing from this cancer genome. The maximum contribution from this mechanism to the SAMMs for MB110 and MB101 was 5.4% and 9.3%, respectively.

The maximum contribution of APOBEC-mediated mutation to the SAMMs of MB110, MB34 and MB101 is 3.9, 1.8, and 0.8%, respectively, and none of these cancer genomes contains a mutation cluster. In fact, none of the 11 cancer genomes examined contains a mutation cluster.

*Pediatric Brain Tumors BMBF (PBCA-DE, ICGC)*

This dataset contains 16 cancer genomes with a sufficient number of somatic autosomal mutations to generate SAMMs. The dendrogram from average linkage clustering is shown in Supplemental Figure 2q. At an AMD of 0.28, there are four clusters; two singleton clusters (DO35739 and DO48901), a cluster containing five SAMMs, and a cluster with the remaining nine SAMMs. DO35589 and DO48909 yield the SAMMs with the smallest AMD to the other SAMMs in their clusters and are representative of these clusters. The SAMM heatmaps and top five mutation frequencies in the SAMMs for DO35739, DO48901, DO35589 and DO48909 are shown in Supplemental Figure 26.

These four SAMMs are very similar to the SAMMs from the Medulloblastoma dataset described above. All four SAMMs contain a definite ^5m^CpG deamination signature, and all of the top five mutation frequencies are compatible with this mutational mechanism. The maximum contribution from this mechanism to the SAMMs is 15.1, 26.0, 27.9 and 35.0% for DO35739, DO48901, DO35589 and DO48909, respectively. Some of these top mutation frequencies may also be due to OxD, whose maximum contribution is 13.7, 7.5, 3.5 and 8.1%, respectively. It is interesting to note that all four SAMMs have a pattern of NRC→NRT transitions, which may also be due to ^5m^CpG deamination and OxD.

The maximum impact of APOBEC-mediated mutations to the four SAMMs is 3.6, 2.1, 1.8 and 0.7%, respectively, and as with the Medulloblastoma dataset, none of the 16 cancer genomes in this dataset contains a mutation cluster with an APOBEC signature.

*Prostate Adenocarcinoma (PRAD-UK, ICGC)*

This dataset contains three cancer genomes with enough somatic autosomal SBSs to generate SAMMs. The average linkage clustering dendrogram for these SAMMs is shown in Supplemental Figure 2r. These three SAMMs are very similar and form a single cluster at an AMD of less than 0.16 (Supplemental Table 2r). The heatmaps and top five mutation frequencies for all three SAMMs (DO36221, DO36223 and DO48577) are shown in Supplemental Figure 27.

All three SAMMs show a definite signature of ^5m^CpG deamination (Supplemental Figure 1c), and all of the top five mutation frequencies are compatibile with this mutational mechanism. The maximum contribution of this mechanism to the SAMMs is 30.3, 37.1 and 34.9%, respectively (Supplemental Table 3s). These top mutation frequencies are also consistent with OxD, whose maximum contribution is 6.4, 9.5 and 9.1%, respectively. There is also a clear pattern of NRC→NRT transitions in these SAMMs, which may be attributed to ^5m^CpG deamination and OxD. While DO36221 and DO36223 each contain one mutation cluster, and DO48577 contains two, and one of them harbors an APOBEC signature. The maximum contribution of APOBEC-mediated mutation is 1.5, 1.5 and 1.6%, respectively.

*Prostate Cancer (Prostate, Sanger)*

This dataset contains seven cancer genomes that generate SAMMs. The average linkage clustering dendrogram for these SAMMs is shown in Supplemental Figure 2s. At an AMD of 0.32, there are two clusters; one containing three SAMMs, and one containing four. PR-1701 and PR-3043 provided the representative SAMMs for these two clusters since their AMDs were smallest to the other two or three SAMMs in their cluster, respectively (Supplemental Table 2s). The SAMM heatmaps and top five mutation frequencies for PR-1701 and PR-3043 are shown in Supplemental Figure 28.

Both SAMMs have C→T transitions within CpG steps as the top five mutations, which are consistent with ^5m^CpG deamination. The maximum contribution of this mechanism to the SAMMs is 26.7% and 18.6%, respectively (Supplemental Table 3t). These mutations are also consistent with OxD whose maximum contribution to the SAMMs is 4.7% and 6.5%, respectively.

The maximum contribution of APOBEC-mediated mutations is 2.6% and 3.0% to the SAMMs of PR-1701 and PR-3043. The PR-1701 contains one mutation cluster but it does not contain an APOBEC signature, while PR-3043 does not contain any mutation clusters in its cancer genome. In fact, none of the seven cancer genomes examined in this dataset contains an APOBEC mutation cluster.

*Renal Cell Cancer (RECA-EU, ICGC)*

This dataset contains 71 cancer genomes with at least 2,000 somatic autosomal SBSs. The average linkage clustering dendrogram for these 71 SAMMs is shown in Supplemental Figure 2t. At an AMD of 0.35, there are five clusters; a singleton cluster (DO46877), a cluster containing DO46897 and DO47112, a cluster with three SAMMs, a cluster with six SAMMs, and a cluster containing the remaining 59 SAMMs. The relative orientation of these 71 SAMMs, color coded by cluster, is shown in Supplemental Figure 29. The AMD of the SAMMs from DO46897 and DO47112 to the other 70 SAMMs is 1.167 and 0.962, respectively (Supplemental Table 2t), so the SAMM of DO46897 was used to represent this cluster. The SAMMs for DO46941, DO46905, and DO47174 were the representative SAMMs for the clusters with three, six and 59 members, respectively, since they have the smallest AMD to the other SAMMs in their clusters. The SAMM heatmaps and top five mutation frequencies for DO46877, DO46897, DO46941, DO46905 and DO47174 are shown in Supplemental Figure 30.

The SAMM for DO46877 looks quite similar to the heatmap for ^5m^CpG deamination (Supplemental Figure 1c). The maximum contribution of this mechanism to this SAMM is 31.8%, while the other three mechanisms examined collectively have a maximum impact of 8.8%. The maximum contribution from APOBEC-mediated mutation is only 0.9% and this cancer genome contains one mutation cluster, but it does not have an APOBEC signature.

The heatmap of the SAMM for DO46897 is completely different, with virtually no ^5m^CpG deamination pattern. The maximum contribution of this mutational mechanism to this SAMM is only 1.8%. All of the top five mutation frequencies are A→T (T→A) transversions, which are not consistent with any of the four mutational mechanisms being considered. Instead, they may be indicative of exposure to aristolochic acid ([Hoang et al. 2013](#_ENREF_2)). Collectively, these four mechanisms have a maximum contribution of only 9.5% to the SAMM of DO46897. This cancer genome does not contain any mutation clusters.

DO46941 generates a SAMM which is between the SAMMs of DO46877 and DO46897. It has an intermediate signature from ^5m^CpG deamination (maximum contribution = 9.3%), and the top four mutation frequencies are consistent with this mechanism. Some of these C→T (G→A) transitions may also be due to OxD, which has a maximum contribution of 10.9% to the total SAMM. The fifth largest mutation frequency is for TAG→TTG (CTA→CAA), which is the highest frequency in DO46897. The maximum contribution from the action of an APOBEC enzyme is 3.7% and this cancer genome does not contain a mutation cluster.

The top five mutation frequencies in the SAMM for DO46905 are also A→T (T→A) transversions, a signature characteristic of exposure to aristolochic acid ([Hoang et al. 2013](#_ENREF_2)). The largest maximum contribution from the four mutation mechanisms considered is from OxD (11.6%). The other three mechanisms combined have a maximum contribution of 11.3%. This cancer genome also contains no mutation clusters.

The SAMM for DO47174 has maximum contributions from ^5m^CpG deamination and OxD of 15.1 and 16.1%, respectively, and all of the top five mutation frequencies are C→T (G→A) transitions within a CpG step. The maximum contribution from photo damage via CPD formation is 6.7% and the SAMM heatmap shows C→T (G→A) transitions in di- and tri-pyrimidine motifs. The maximum contribution from APOBEC-mediated mutation to the SAMM for DO47174 is 5.9%. This cancer genome contains one mutation cluster, but it does not have an APOBEC signature.

**SUPPLEMENTAL REFERENCES**

Berger MF, Hodis E, Heffernan TP, Deribe YL, Lawrence MS, Protopopov A, Ivanova E, Watson IR, Nickerson E, Ghosh P, Zhang H, Zeid R, Ren X, Cibulskis K, Sivachenko AY, Wagle N, Sucker A, Sougnez C, Onofrio R, Ambrogio L, Auclair D, Fennell T, Carter SL, Drier Y, Stojanov P, Singer MA, Voet D, Jing R, Saksena G, Barretina J, Ramos AH, Pugh TJ, Stransky N, Parkin M, Winckler W, Mahan S, Ardlie K, Baldwin J, Wargo J, Schadendorf D, Meyerson M, Gabriel SB, Golub TR, Wagner SN, Lander ES, Getz G, Chin L, Garraway LA (2012) Melanoma genome sequencing reveals frequent PREX2 mutations. Nature 485: 502-6. doi: 10.1038/nature11071

Hoang ML, Chen CH, Sidorenko VS, He J, Dickman KG, Yun BH, Moriya M, Niknafs N, Douville C, Karchin R, Turesky RJ, Pu YS, Vogelstein B, Papadopoulos N, Grollman AP, Kinzler KW, Rosenquist TA (2013) Mutational signature of aristolochic acid exposure as revealed by whole-exome sequencing. Sci Transl Med 5: 197ra102. doi: 10.1126/scitranslmed.3006200

Roberts SA, Gordenin DA (2014) Clustered and genome-wide transient mutagenesis in human cancers: Hypermutation without permanent mutators or loss of fitness. Bioessays. doi: 10.1002/bies.201300140

**SUPPLEMENTAL TABLES AND FIGUGES LEGENDS**

**Supplemental Table 1**: Canonical mechanistic template mutation matrices (MTMM) representing a) oxidative damage, b) photo damage by CPD formation, c) ^5m^CpG deamination, and d) deamination by an APOBEC enzyme.

**Supplemental Table 2**: Manhattan distances between the somatic autosomal mutation matrices of the samples in each dataset listed in Table 1; a) LAML-KR, b) BRCA-UK, c) Breast, d) ESAD-UK, e) LINC-JP, f) LIRI-JP, g) Liver, h) Lung_Adeno, i) MALY-DE, j) Melanoma, k) OV-AU, l) PACA-CA, m) PACA-AU, n) PAEN-AU, o) Pancreas, p) Medulloblastoma, q) PBCA-DE, r) PRAD-UK, s) Prostate, and t) RECA-EU. There is no entry for the ALL dataset since this contains only a single cancer genome.

**Supplemental Table 3**: Maximum impact from each of the canonical mutational mechanisms in the somatic autosomal mutation matrices of the samples in each dataset listed in Table 1; a) ALL, b) LAML-KR, c) BRCA-UK, d) Breast, e) ESAD-UK, f) LINC-JP, g) LIRI-JP, h) Liver, i) Lung_Adeno, j) MALY-DE, k) Melanoma, l) OV-AU, m) PACA-CA, n) PACA-AU, o) PAEN-AU, p) Pancreas, q) Medulloblastoma, r) PBCA-DE, s) PRAD-UK, t) Prostate, and u) RECA-EU.

**Supplemental Table 4**: Nearest neighbor to each of the 908 samples’ SAMM for all cancer genomes examined except the single ALL genome.

**Supplemental Table 5**: a) Sample groupings used in the distance dependent *k*-nearest neighbor classifier. b) Results using a distance-dependent 6-nearest neighbor classifier when the un-normalized probability of belonging to a neighbor’s group is 0.5 when the Manhattan distance is 0.5 (*D*_0.5_ = 0.5), and the final normalized probability must be at least 0.5 for assignment. c) Results using a distance-dependent 6-nearest neighbor classifier when the un-normalized probability of belonging to a neighbor’s group is 0.5 when the Manhattan distance is 0.5 (*D*_0.5_ = 0.5), and the final assignment is made to the Type with the largest overall probability (Maximum Likelihood). d) Overall results when the group assignment requires a normalized probability of at least 0.5 and the number of nearest neighbors varies from 3 to 8 (*D*_0.5_ = 0.5). e) Overall results when the group assignment results from the largest normalized probability (Maximum Likelihood) and the number of nearest neighbors varies from 3 to 8 (*D*_0.5_ = 0.5). f) Overall results when the group assignment requires a normalized probability of at least 0.5 and the number of nearest neighbors is 6 and *D*_0.5_ varies from 0.4 to 0.7. g) Overall results when the group assignment results from the largest normalized probability (Maximum Likelihood) and the number of nearest neighbors is 6 and *D*_0.5_ varies from 0.4 to 0.7. h) Overall results using a standard *k*-nearest neighbor when the group assignment requires a normalized probability of at least 0.5 and the number of nearest neighbors varies from 3 to 8. i) Overall results using a standard *k*-nearest neighbor. The group assignment results from the largest normalized probability (Maximum Likelihood) and the number of nearest neighbors varies from 3 to 8. j) Results using a standard 6-nearest neighbor classifier and the final normalized probability must be at least 0.5 for assignment. k) Results using a standard 6-nearest neighbor classifier when the final assignment is made to the Tumor Type with the largest overall probability (Maximum Likelihood).

**Supplemental Table 6**: Predicted cancer tissue using both the requirement that the probability be at least 0.5 and a maximum likelihood criterion for the 904 samples listed in Supplemental Table 5a, as well as the normalized probabilities of belonging to each tissue type and the Undetermined group, for a distance-dependent 6-nearest neighbor classifiers with *D*_0.5_ = 0.5.

**Supplemental Figure 1**: Heatmaps for the mechanistic template mutation matrices (MTMMs) representing a) oxidative damage, b) photo damage resulting from CPD formation, c) ^5m^CpG deamination, and d) the action of APOBEC.

**Supplemental Figure 2**: Dendrograms resulting from an average linkage clustering of the SAMMs based on Manhattan distances for each dataset containing more than two samples; a) LAML-KR, b) BRCA-UK, c) Breast, d) ESAD-UK, e) LINC-JP, f) LIRI-JP, g) Liver, h) Lung_Adeno, i) MALY-DE, j) Melanoma, k) OV-AU, l) PACA-CA, m) PACA-AU, n) PAEN-AU, o) Pancreas, p) Medulloblastoma, q) PBCA-DE, r) PRAD-UK, s) Prostate, t) RECA-EU. There is no dendrogram for the ALL dataset since it only contains a single cancer genome.

**Supplemental Figure 3**: SAMM heatmap for the single sample from the Acute Lymphoblastic Leukemia (ALL, Sanger) dataset and the top five scaled mutation frequencies.

**Supplemental Figure 4**: SAMM heatmaps and top five scaled mutation frequencies for DO49532 and DO49535 from the Acute Myeloid Leukemia (LAML-KR, ICGC) dataset.

**Supplemental Figure 5**: SAMM heatmaps and top five scaled mutation frequencies for DO1003, DO1010, DO1016, and DO1076 from the Breast Triple Negative Lobular Cancer (BRCA-UK, ICGC) dataset.

**Supplemental Figure 6**: Sammon map showing the relative positions of the 77 GWS cancer genomes from the Breast Cancer (Breast, Sanger) dataset. The positions of the samples are color coded to represent the 10 clusters present at an average Manhattan distance of 0.34.

**Supplemental Figure 7**: SAMM heatmaps and top five scaled mutation frequencies for PD4266a, PD6722a, PD4005a, PD4120a, PD7404a, PD4224a, PD4980a, PD4315a, PD4965a, and PD4952a from the Breast Cancer (Breast, Sanger) dataset.

**Supplemental Figure 8**: SAMM heatmaps and top five scaled mutation frequencies for DO10842, DO10840, DO10843, and DO10853 from the Esophageal Adenocarcinoma (ESAD-UK, ICGC) dataset.

**Supplemental Figure 9**: SAMM heatmaps and top five scaled mutation frequencies for DO45031, DO45069, DO45061, DO45041, and DO45047 from the Liver Cancer NCC (LINC-JP, ICGC) dataset.

**Supplemental Figure 10**: Sammon map showing the relative positions of the 188 GWS cancer genomes from the Liver Cancer RIKEN (LIRI-JP, ICGC) dataset. The positions of the samples are color coded to represent the 7 clusters present at an average Manhattan distance of 0.33.

**Supplemental Figure 11**: SAMM heatmaps and top five scaled mutation frequencies for DO45157, DO45301, DO45293, DO48682, DO45211, DO45305, and DO23521 from the Liver Cancer RIKEN (LIRI-JP, ICGC) dataset.

**Supplemental Figure 12**: Sammon map showing the relative positions of the 84 GWS cancer genomes from the Liver Cancer (Liver, Sanger) dataset. The positions of the samples are color coded to represent the 6 clusters present at an average Manhattan distance of 0.35.

**Supplemental Figure 13**: SAMM heatmaps and top five scaled mutation frequencies for HX13T, HX16T, RK126_C01, RK138_C01, HX23T, and RK051_C01 from the Liver Cancer (Liver, Sanger) dataset.

**Supplemental Figure 14**: SAMM heatmaps and top five scaled mutation frequencies for LUAD-S01345, LU-A08-43, LUAD-S01341, and LUAD-FH5PJ from the Lung Adenocarcinoma (Lung_Adeno, Sanger) dataset.

**Supplemental Figure 15**: SAMM heatmaps and top five scaled mutation frequencies for DO27771, DO27777, DO27779, DO27813, and DO27805 from the Malignant Lymphoma DKFZ (MALY-DE, ICGC) dataset.

**Supplemental Figure 16**: SAMM heatmaps and top five scaled mutation frequencies for ME015, ME009, ME021, and ME020 from the Melanoma (Melanoma, ([Berger et al. 2012](#_ENREF_1))) dataset.

**Supplemental Figure 17**: Sammon map showing the relative positions of the 89 GWS cancer genomes from the Ovarian Cancer QCMG (OV-AU, ICGC) dataset. The positions of the samples are color coded to represent the 6 clusters present at an average Manhattan distance of 0.30.

**Supplemental Figure 18**: SAMM heatmaps and top five scaled mutation frequencies for DO46330, DO46588, DO46591, DO46396, DO46356, and DO46473 from the Ovarian Cancer QCMG (OV-AU, ICGC) dataset.

**Supplemental Figure 19**: Sammon map showing the relative positions of the 45 SAMMs representing the GWS cancer genomes from the Pancreatic Cancer OICR (PACA-CA, ICGC) dataset. The positions of the samples are color coded to represent the 4 clusters present at an average Manhattan distance of 0.28.

**Supplemental Figure 20**: SAMM heatmaps and top five scaled mutation frequencies for DO35442, DO49418, DO49419, and DO49430 from the Pancreatic Cancer OICR (PACA-CA, ICGC) dataset.

**Supplemental Figure 21**: Sammon map showing the relative positions of the 137 SAMMs representing the GWS cancer genomes from the Pancreatic Cancer QCMG (PACA-AU, ICGC) dataset. The positions of the samples are color coded to represent the seven clusters present at an average Manhattan distance of 0.40.

**Supplemental Figure 22**: SAMM heatmaps and top five scaled mutation frequencies for DO32819, DO32976, DO33043, DO33056, DO34128, DO49087, and DO49130 from the Pancreatic Cancer QCMG (PACA-AU, ICGC) dataset.

**Supplemental Figure 23**: SAMM heatmaps and top five scaled mutation frequencies for DO46779, DO46749, DO46785, and DO48581 from the Pancreatic Cancer Endocrine Neoplasms QCMG (PAEN-AU, ICGC) dataset.

**Supplemental Figure 24**: SAMM heatmaps and top five scaled mutation frequencies for APGI_2137, APGI_2157, APGI_2057, and APGI_2353 from the Pancreatic Cancer (Pancreas, Sanger) dataset.

**Supplemental Figure 25**: SAMM heatmaps and top five scaled mutation frequencies for MD110, MB34, and MB101 from the Medulloblastoma (Medulloblastoma, Sanger) dataset.

**Supplemental Figure 26**: SAMM heatmaps and top five scaled mutation frequencies for DO35739, DO48901, DO35589, and DO48909 from the Pediatric Brain Tumors BMBF (PBCA-DE, ICGC) dataset.

**Supplemental Figure 27**: SAMM heatmaps and top five scaled mutation frequencies for DO36221, DO36223, and DO48577 from the Prostate Adenocarcinoma (PRAD-UK, ICGC) dataset.

**Supplemental Figure 28**: SAMM heatmaps and top five scaled mutation frequencies for PR-1701 and PR-3043 from the Prostate Cancer (Prostate, Sanger) dataset.

**Supplemental Figure 29**: Sammon map showing the relative positions of the 71 SAMMs representing the GWS cancer genomes from the Renal Cell Cancer (RECA-EU, ICGC) dataset. The positions of the samples are color coded to represent the five clusters present at an average Manhattan distance of 0.35.

**Supplemental Figure 30**: SAMM heatmaps and top five scaled mutation frequencies for DO46877, DO46897, DO46941, DO46905, and DO47174 from the Renal Cell Cancer (RECA-EU, ICGC) dataset.

**Supplemental Table 5**

a

| **Group** | **#Samples** | **Type** | **Dataset(s)** |
| --- | --- | --- | --- |
| 1 | 95 | Breast | BRCA-UK_ICGC, Breast_sanger |
| 2 | 16 | Esophagus | ESAD-UK |
| 3 | 303 | Liver | LINC-JP_ICGC, LIRI-JP_ICGC, Liver_Sanger |
| 4 | 23 | Lung | Lung_Adeno_Sanger |
| 5 | 37 | Lymphoma | MALY-DE_ICGC |
| 6 | 25 | Melanoma | Melanoma_Berger |
| 7 | 89 | Ovarian | OV-AU_ICGC |
| 8 | 208 | Pancreatic | PACA-CA_ICGC, PACA-AU_ICGC, PAEN-AU_ICGC, Pancreas_Sanger |
| 9 | 27 | Brain | Medulloblastoma_Sanger, PBCA-DE_ICGC |
| 10 | 10 | Prostate | PRAD-UK_ICGC, Prostate_Sanger |
| 11 | 71 | Renal | RECA-EU_ICGC |

b

| **Type** | **#Correct** | **#Wrong** | **#Undetermined** | **%Correct** | **%Undetermined** |
| --- | --- | --- | --- | --- | --- |
| Breast | 94 | 0 | 1 | 100.00 | 1.05 |
| Esophagus | 8 | 7 | 1 | 53.33 | 6.25 |
| Liver | 288 | 6 | 9 | 97.96 | 2.97 |
| Lung | 15 | 7 | 1 | 68.18 | 4.35 |
| Lymphoma | 24 | 6 | 7 | 80.00 | 18.92 |
| Melanoma | 20 | 3 | 2 | 86.96 | 8.00 |
| Ovarian | 54 | 19 | 16 | 73.97 | 17.98 |
| Pancreatic | 153 | 28 | 27 | 84.53 | 12.98 |
| Brain | 6 | 16 | 5 | 27.27 | 18.52 |
| Prostate | 0 | 6 | 4 | 0.00 | 40.00 |
| Renal | 54 | 10 | 7 | 84.38 | 9.86 |
| **Overall** | 716 | 108 | 80 | 86.89 | 8.85 |

c

| **Type** | **#Correct** | **#Wrong** | **#Undetermined** | **%Correct** | **%Undetermined** |
| --- | --- | --- | --- | --- | --- |
| Breast | 94 | 0 | 1 | 100.00 | 1.05 |
| Esophagus | 8 | 8 | 0 | 50.00 | 0.00 |
| Liver | 295 | 8 | 0 | 97.36 | 0.00 |
| Lung | 15 | 8 | 0 | 65.22 | 0.00 |
| Lymphoma | 28 | 9 | 0 | 75.68 | 0.00 |
| Melanoma | 22 | 3 | 0 | 88.00 | 0.00 |
| Ovarian | 64 | 25 | 0 | 71.91 | 0.00 |
| Pancreatic | 163 | 45 | 0 | 78.37 | 0.00 |
| Brain | 7 | 20 | 0 | 25.93 | 0.00 |
| Prostate | 0 | 10 | 0 | 0.00 | 0.00 |
| Renal | 58 | 13 | 0 | 81.69 | 0.00 |
| **Overall** | 754 | 149 | 1 | 83.50 | 0.11 |

d

| **#Neighbors** | **#Correct** | **#Wrong** | **#Undetermined** | **%Correct** | **%Undetermined** |
| --- | --- | --- | --- | --- | --- |
| **3** | 764 | 117 | 23 | 86.72 | 2.54 |
| **4** | 735 | 89 | 80 | 89.20 | 8.85 |
| **5** | 739 | 123 | 42 | 85.73 | 4.65 |
| **6** | 716 | 108 | 80 | 86.89 | 8.85 |
| **7** | 729 | 127 | 48 | 85.16 | 5.31 |
| **8** | 711 | 114 | 79 | 86.18 | 8.74 |

e

| **#Neighbors** | **#Correct** | **#Wrong** | **#Undetermined** | **%Correct** | **%Undetermined** |
| --- | --- | --- | --- | --- | --- |
| **3** | 777 | 126 | 1 | 86.05 | 0.11 |
| **4** | 766 | 137 | 1 | 84.83 | 0.11 |
| **5** | 756 | 147 | 1 | 83.72 | 0.11 |
| **6** | 754 | 149 | 1 | 83.50 | 0.11 |
| **7** | 748 | 155 | 1 | 82.83 | 0.11 |
| **8** | 744 | 159 | 1 | 82.39 | 0.11 |

f

| ***D*_0.5_** | **#Correct** | **#Wrong** | **#Undetermined** | **%Correct** | **%Undetermined** |
| --- | --- | --- | --- | --- | --- |
| **0.4** | 715 | 106 | 83 | 87.09 | 9.18 |
| **0.5** | 716 | 108 | 80 | 86.89 | 8.85 |
| **0.6** | 717 | 110 | 77 | 86.70 | 8.52 |
| **0.7** | 719 | 110 | 75 | 86.73 | 8.30 |

g

| ***D*_0.5_** | **#Correct** | **#Wrong** | **#Undetermined** | **%Correct** | **%Undetermined** |
| --- | --- | --- | --- | --- | --- |
| **0.4** | 754 | 149 | 1 | 83.50 | 0.11 |
| **0.5** | 754 | 149 | 1 | 83.50 | 0.11 |
| **0.6** | 754 | 149 | 1 | 83.50 | 0.11 |
| **0.7** | 754 | 149 | 1 | 83.50 | 0.11 |

h

| **#Neighbors** | **#Correct** | **#Wrong** | **#Undetermined** | **%Correct** | **%Undetermined** |
| --- | --- | --- | --- | --- | --- |
| **3** | 761 | 124 | 19 | 85.99 | 2.10 |
| **4** | 773 | 130 | 1 | 85.60 | 0.11 |
| **5** | 739 | 135 | 30 | 84.55 | 3.32 |
| **6** | 748 | 139 | 17 | 84.33 | 1.88 |
| **7** | 728 | 138 | 38 | 84.06 | 4.20 |
| **8** | 731 | 147 | 26 | 83.26 | 2.88 |

i

| **#Neighbors** | **#Correct** | **#Wrong** | **%Correct** |
| --- | --- | --- | --- |
| **3** | 778 | 126 | 86.06 |
| **4** | 774 | 130 | 85.62 |
| **5** | 756 | 148 | 83.63 |
| **6** | 758 | 146 | 83.85 |
| **7** | 749 | 155 | 82.85 |
| **8** | 746 | 158 | 82.52 |

j

| **Type** | **#Correct** | **#Wrong** | **#Undetermined** | **%Correct** | **%Undetermined** |
| --- | --- | --- | --- | --- | --- |
| Breast | 95 | 0 | 0 | 100.00 | 0.00 |
| Esophagus | 8 | 8 | 0 | 50.00 | 0.00 |
| Liver | 294 | 9 | 0 | 97.03 | 0.00 |
| Lung | 15 | 8 | 0 | 65.22 | 0.00 |
| Lymphoma | 27 | 7 | 3 | 79.41 | 8.11 |
| Melanoma | 21 | 4 | 0 | 84.00 | 0.00 |
| Ovarian | 65 | 23 | 1 | 73.86 | 1.12 |
| Pancreatic | 161 | 42 | 5 | 79.31 | 2.40 |
| Brain | 8 | 17 | 2 | 32.00 | 7.41 |
| Prostate | 0 | 8 | 2 | 0.00 | 20.00 |
| Renal | 54 | 13 | 4 | 80.60 | 5.63 |
| **Overall** | 748 | 139 | 17 | 84.33 | 1.88 |

k

| **Type** | **#Correct** | **#Wrong** | **%Correct** |
| --- | --- | --- | --- |
| Breast | 95 | 0 | 100.00 |
| Esophagus | 8 | 8 | 50.00 |
| Liver | 294 | 9 | 97.03 |
| Lung | 15 | 8 | 65.22 |
| Lymphoma | 29 | 8 | 78.38 |
| Melanoma | 21 | 4 | 84.00 |
| Ovarian | 66 | 23 | 74.16 |
| Pancreatic | 164 | 44 | 78.85 |
| Brain | 8 | 19 | 29.63 |
| Prostate | 0 | 10 | 0.00 |
| Renal | 58 | 13 | 81.69 |
| **Overall** | 758 | 146 | 83.85 |

**SUPPLEMENTAL FIGURES**

**Supplemental Figure 1**

| a  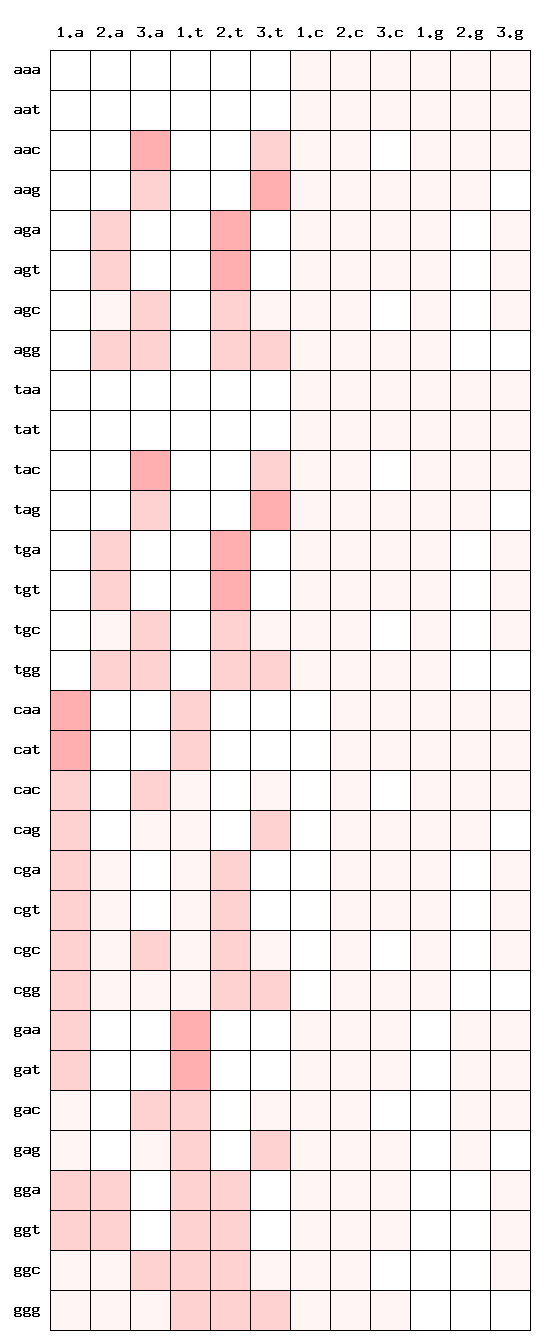 | b  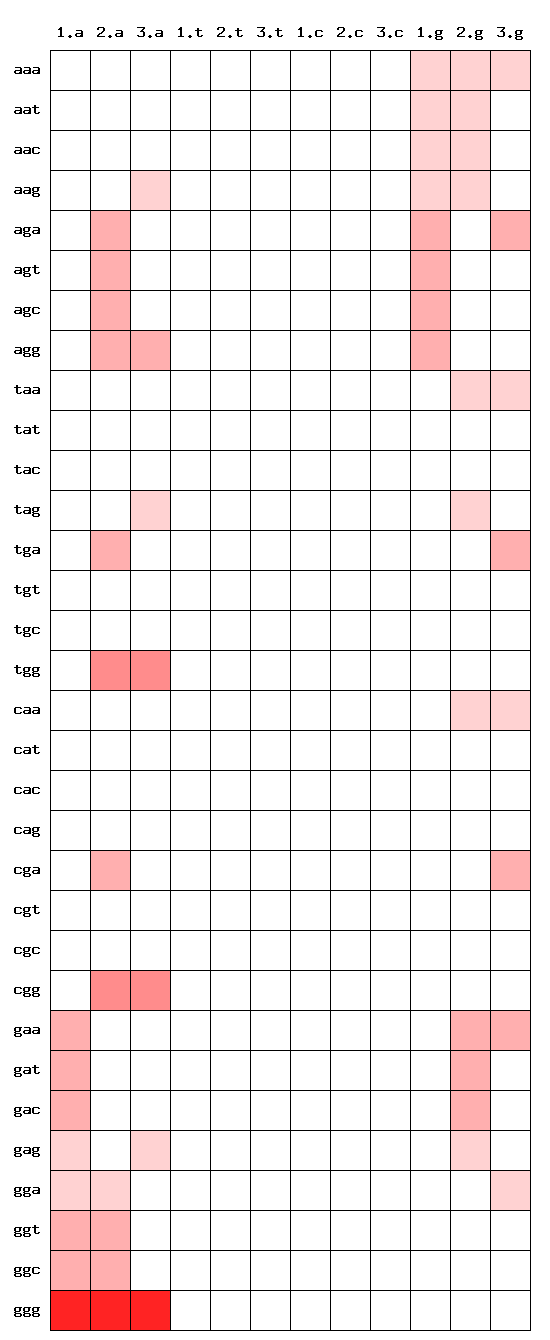 |
| --- | --- |

| c  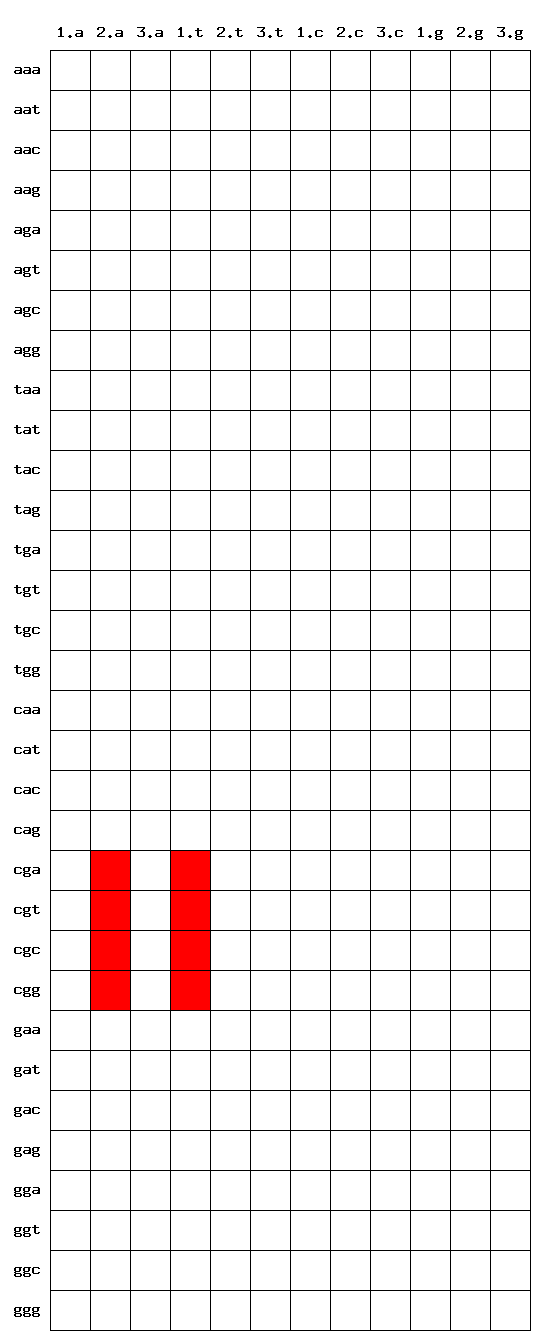 | d  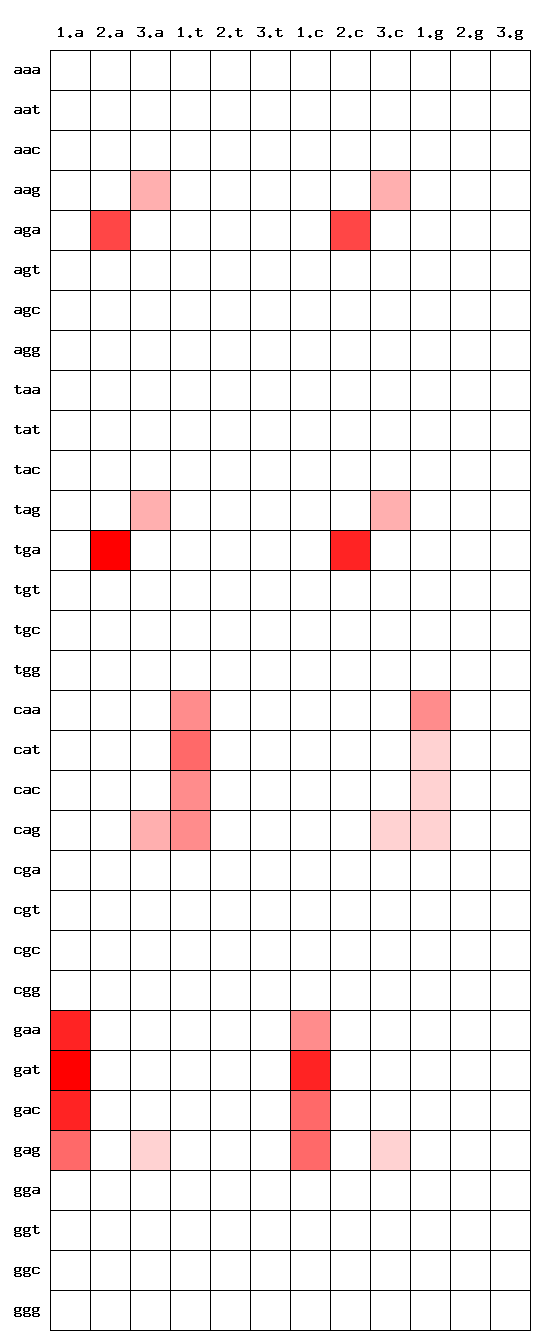 |
| --- | --- |

**Supplemental Figure 2**

a) LAML-KR

**
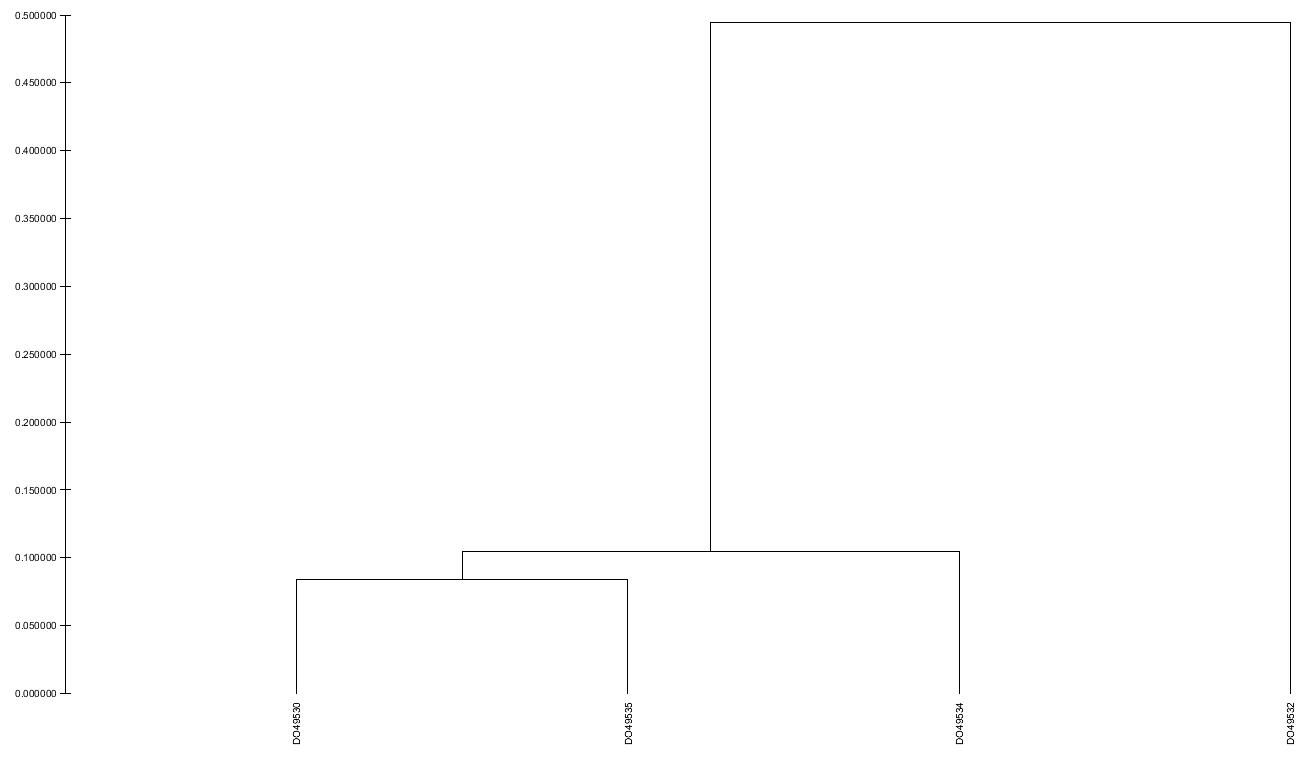
**

b) BRCA-UK


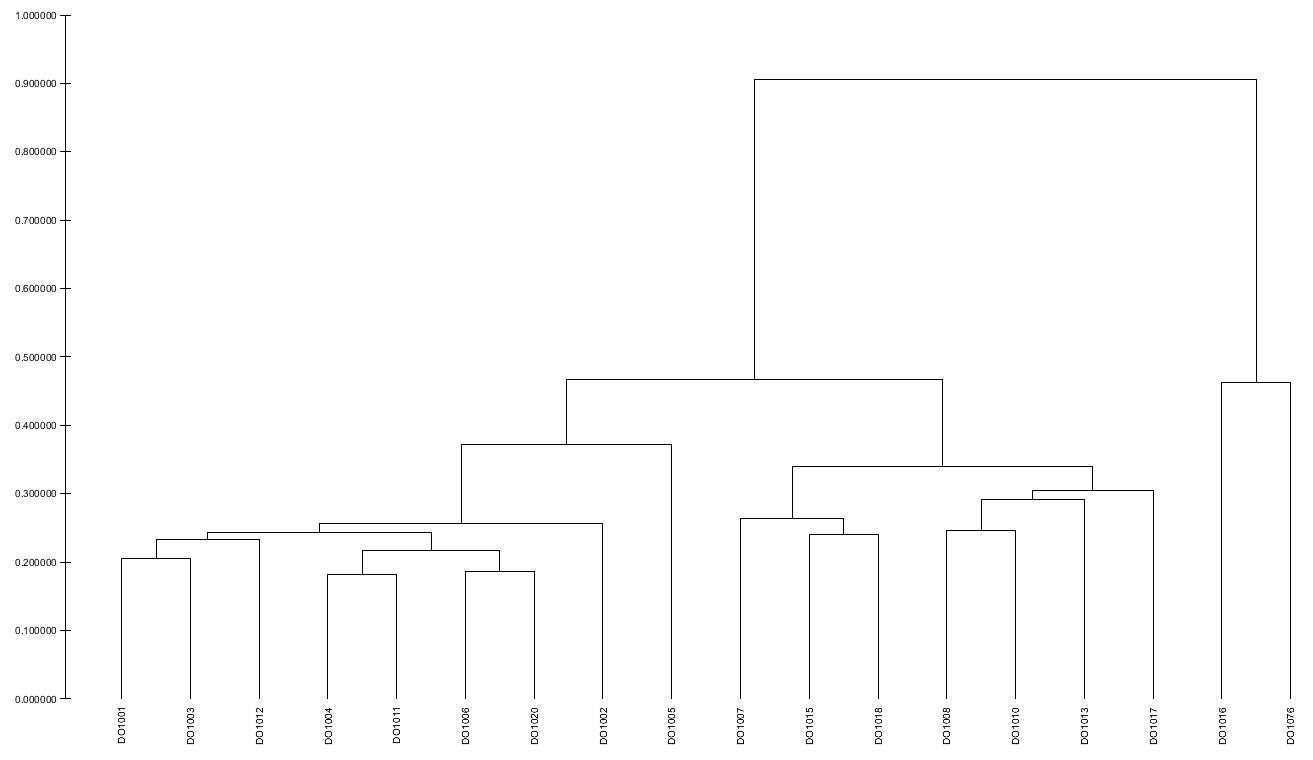


c) Breast


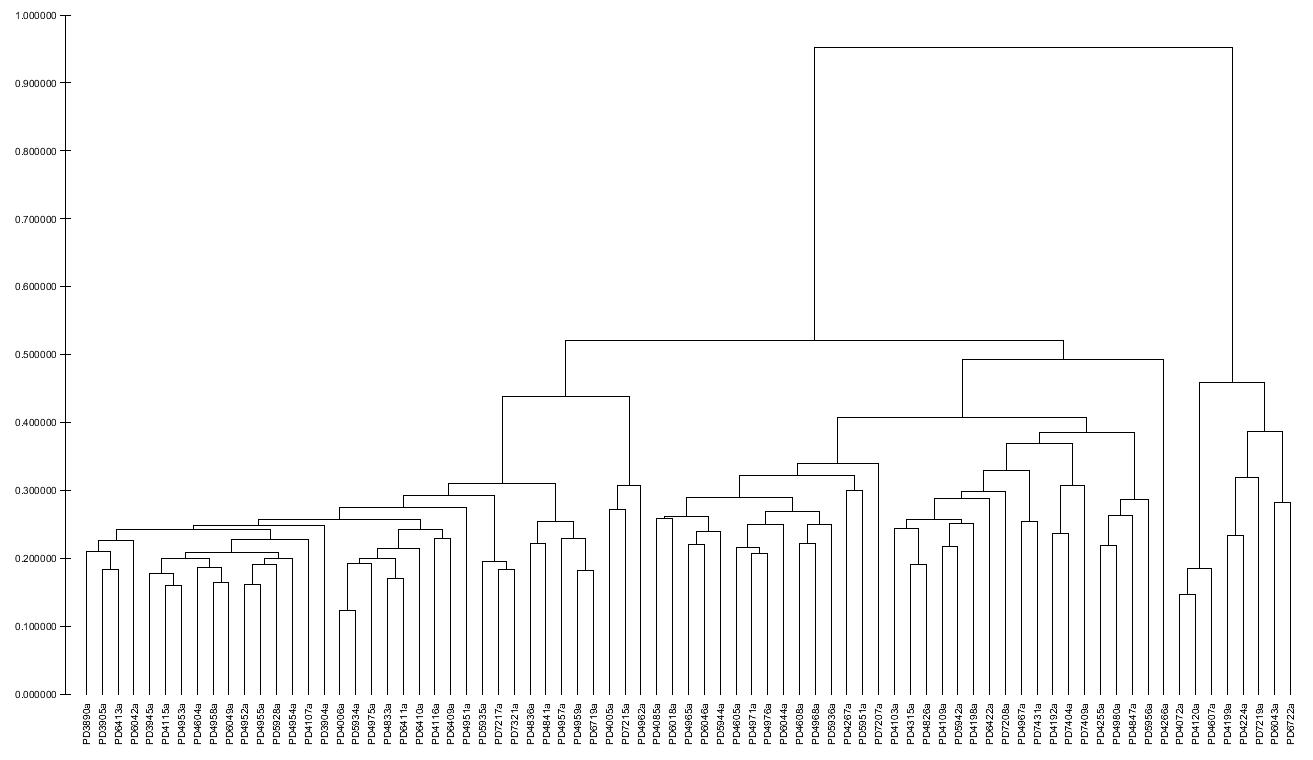


d) ESAD-UK


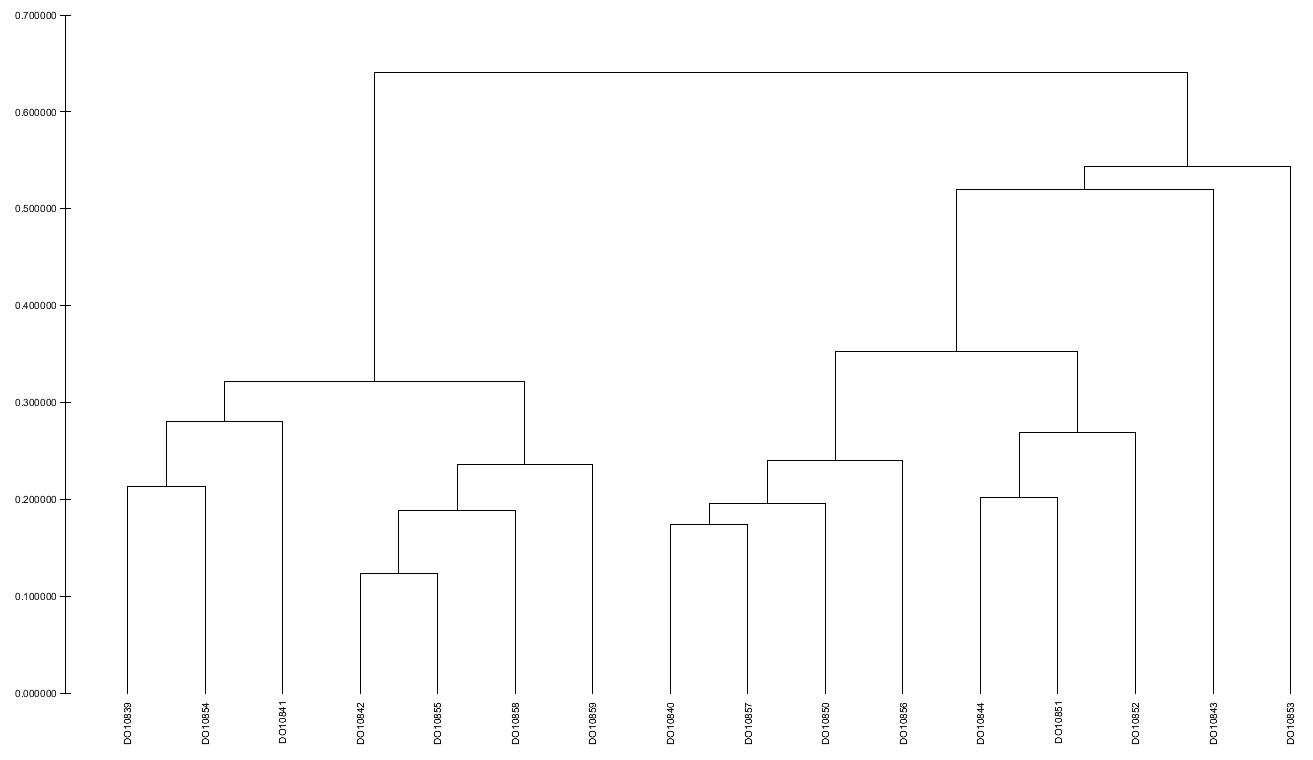


e) LINC-JP


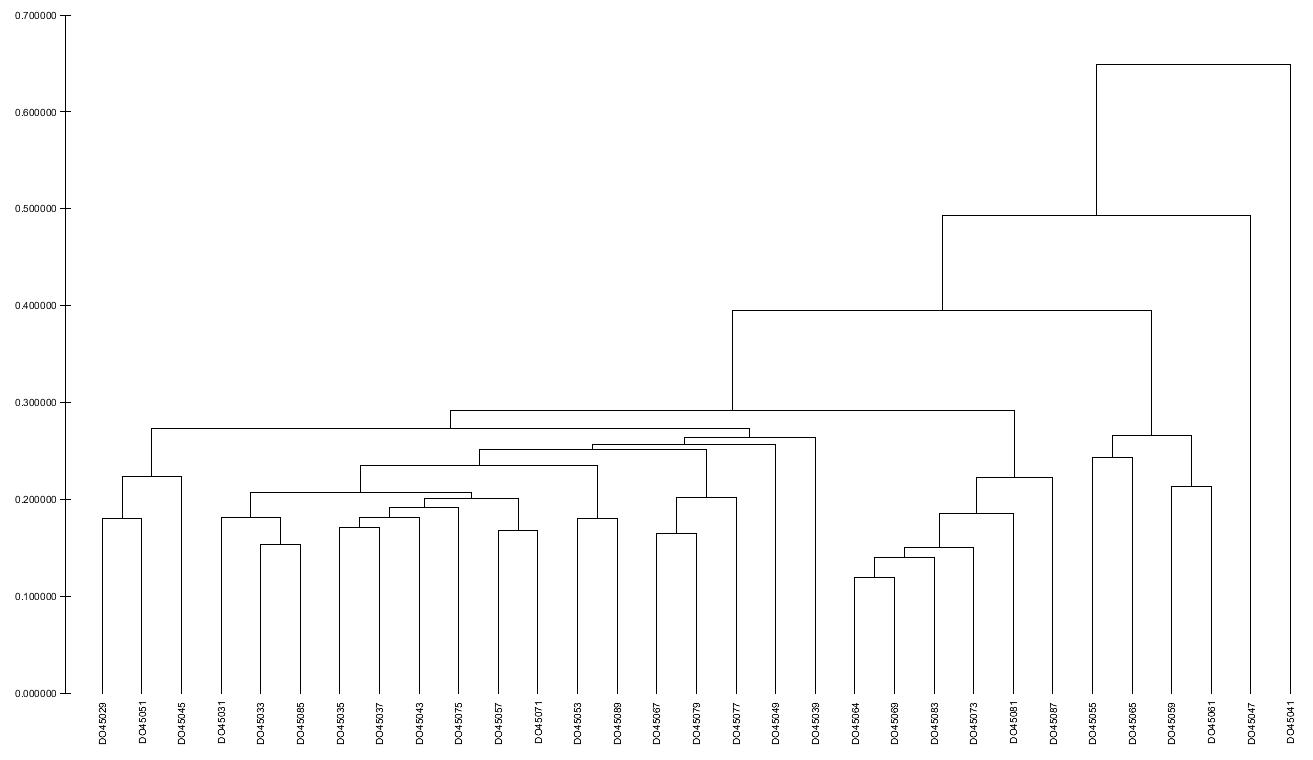


f) LIRI-JP


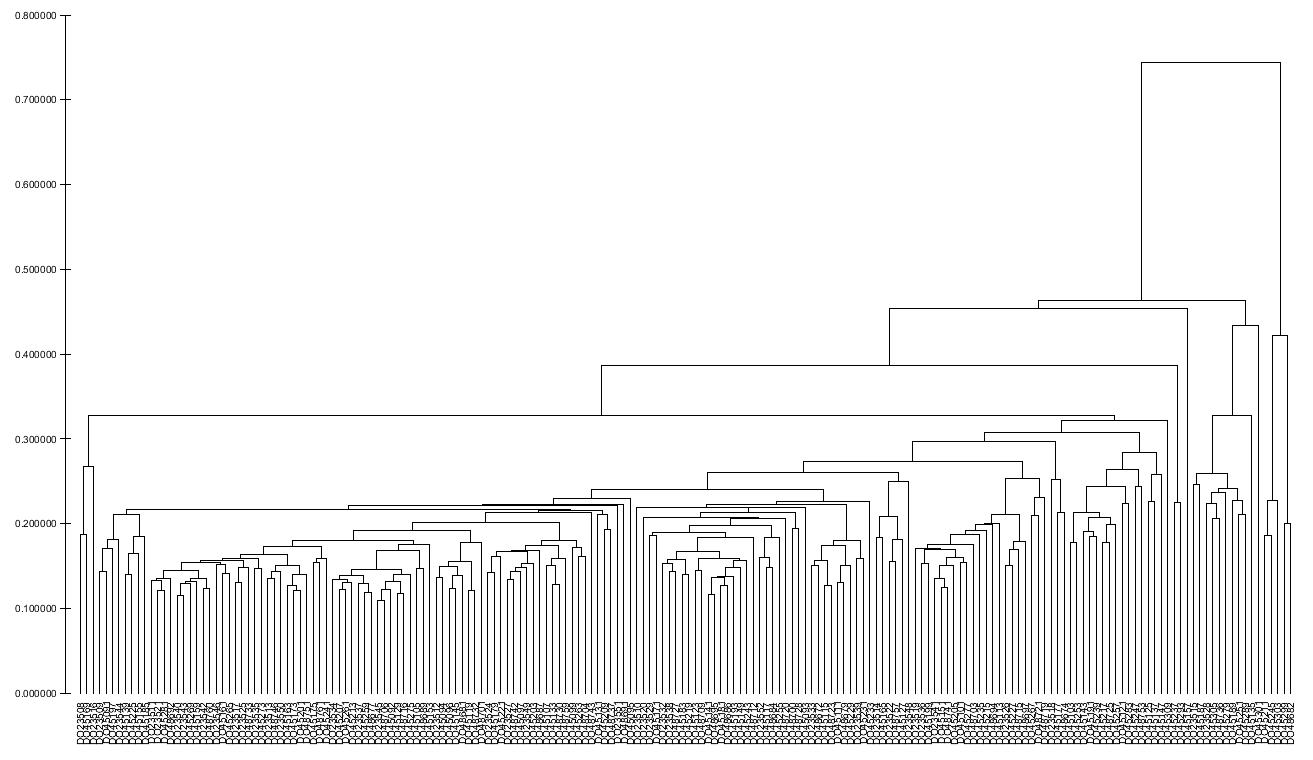


g) Liver


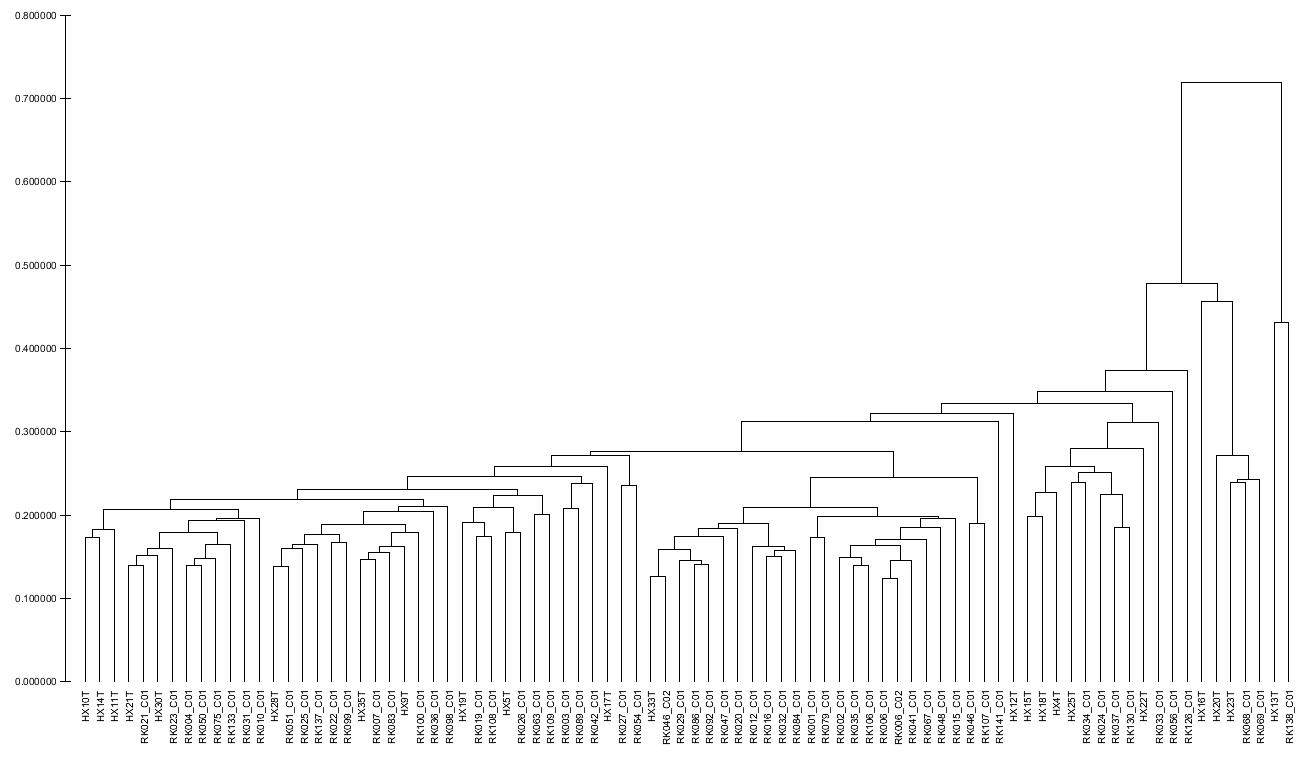


h) Lung_Adeno


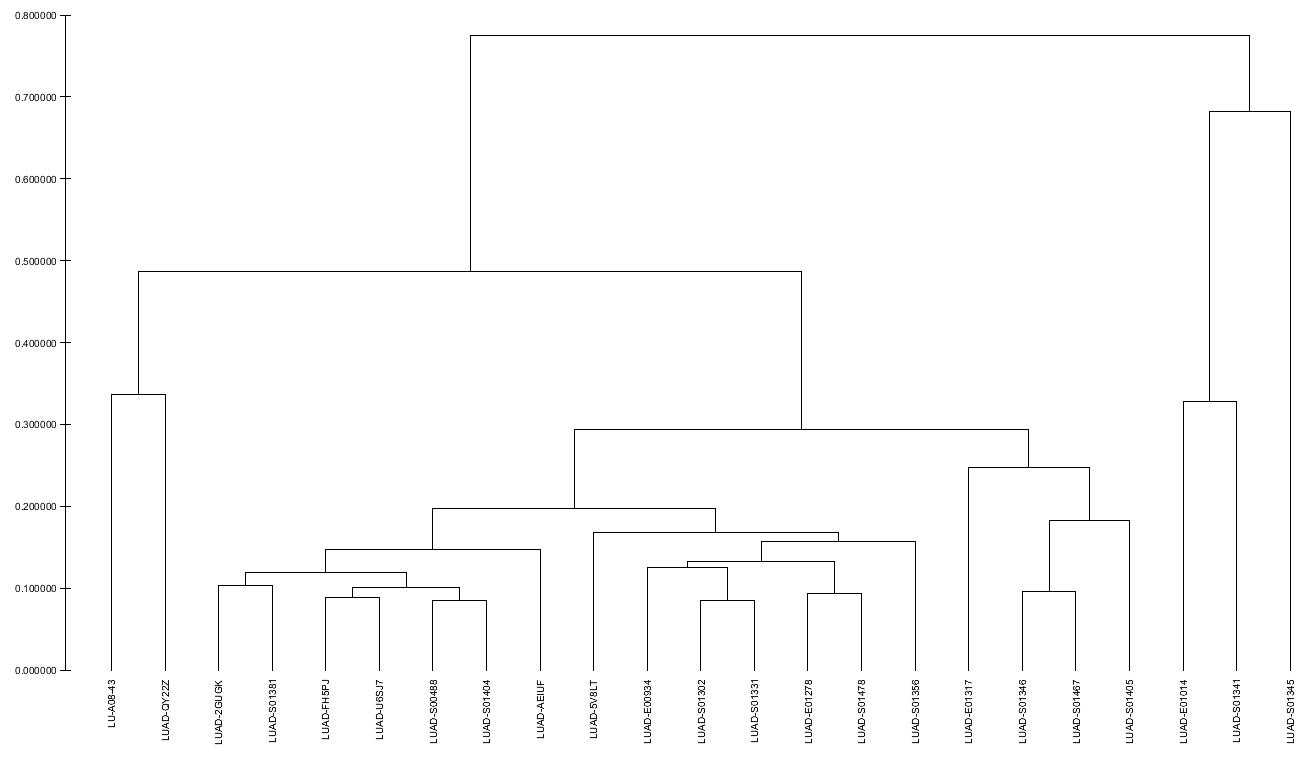


i) MALY-DE


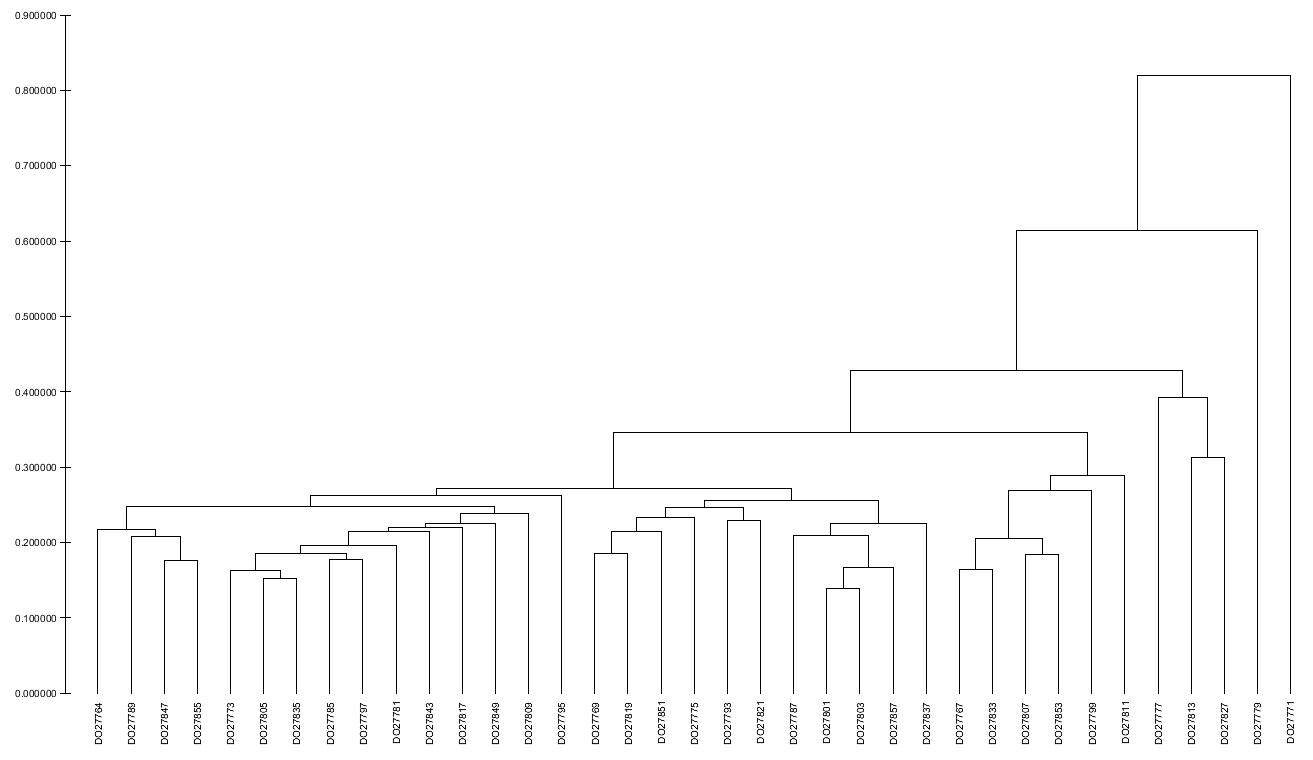


j) Melanoma


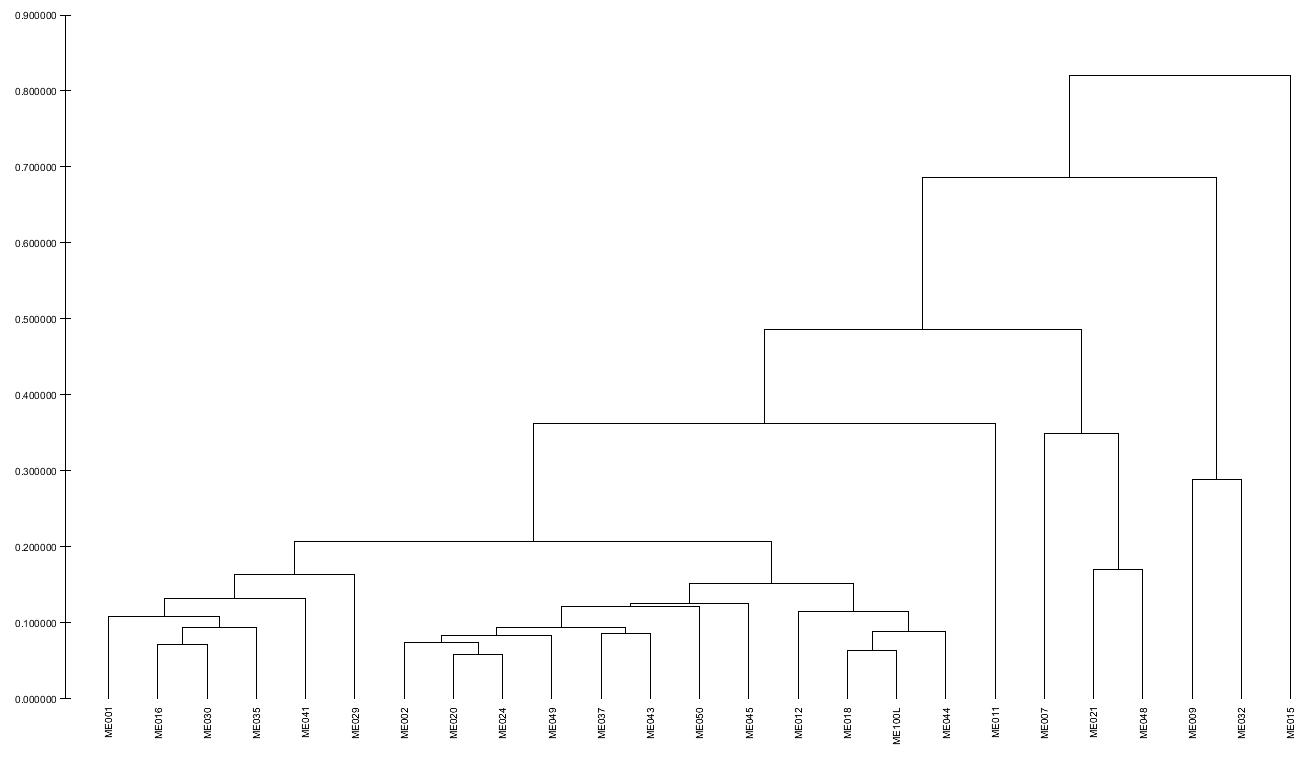


k) OV-AU

**
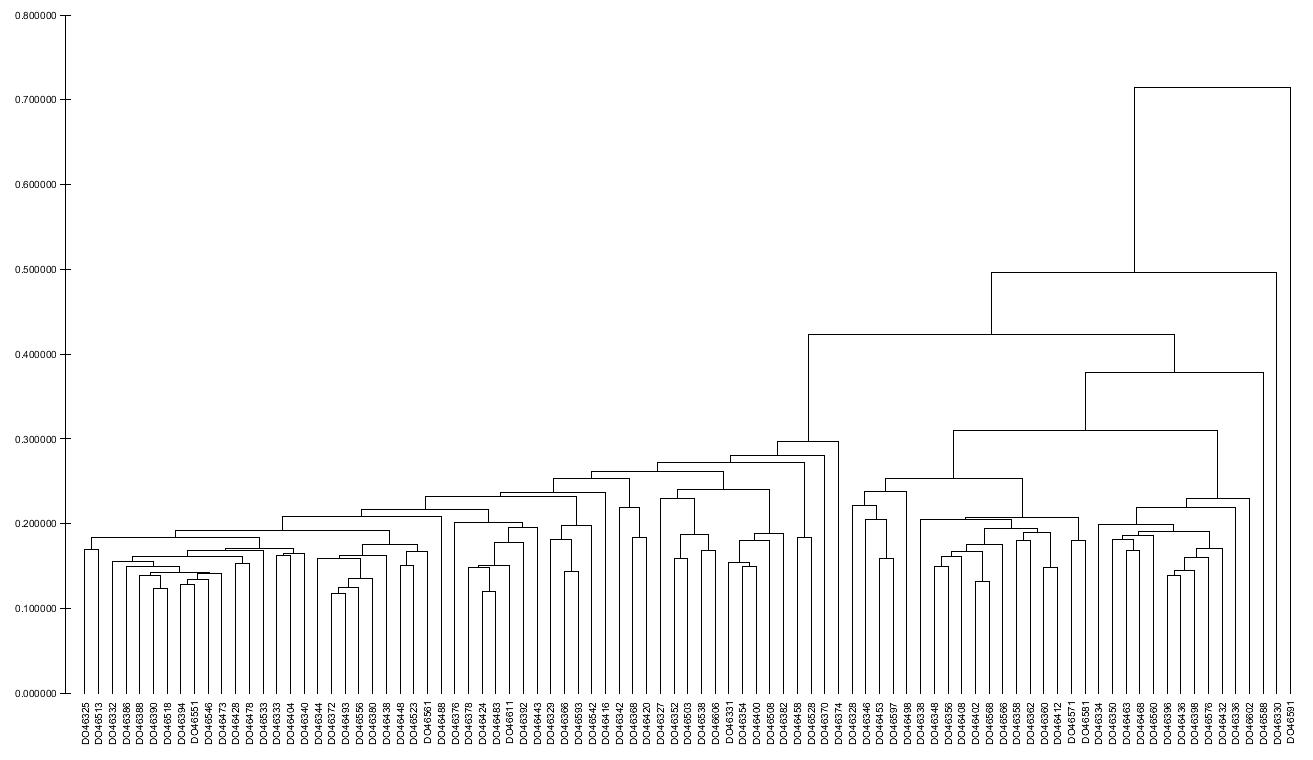
**

l) PACA-CA


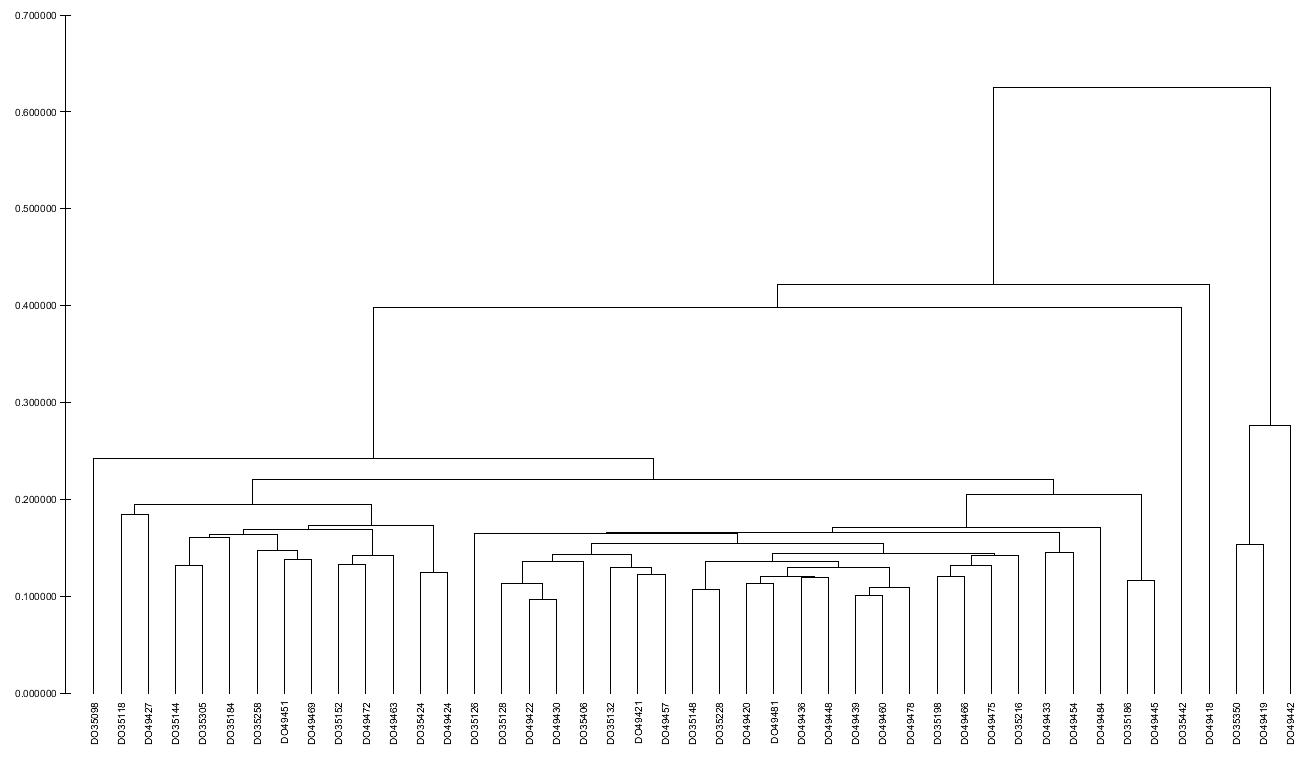


m) PACA-AU


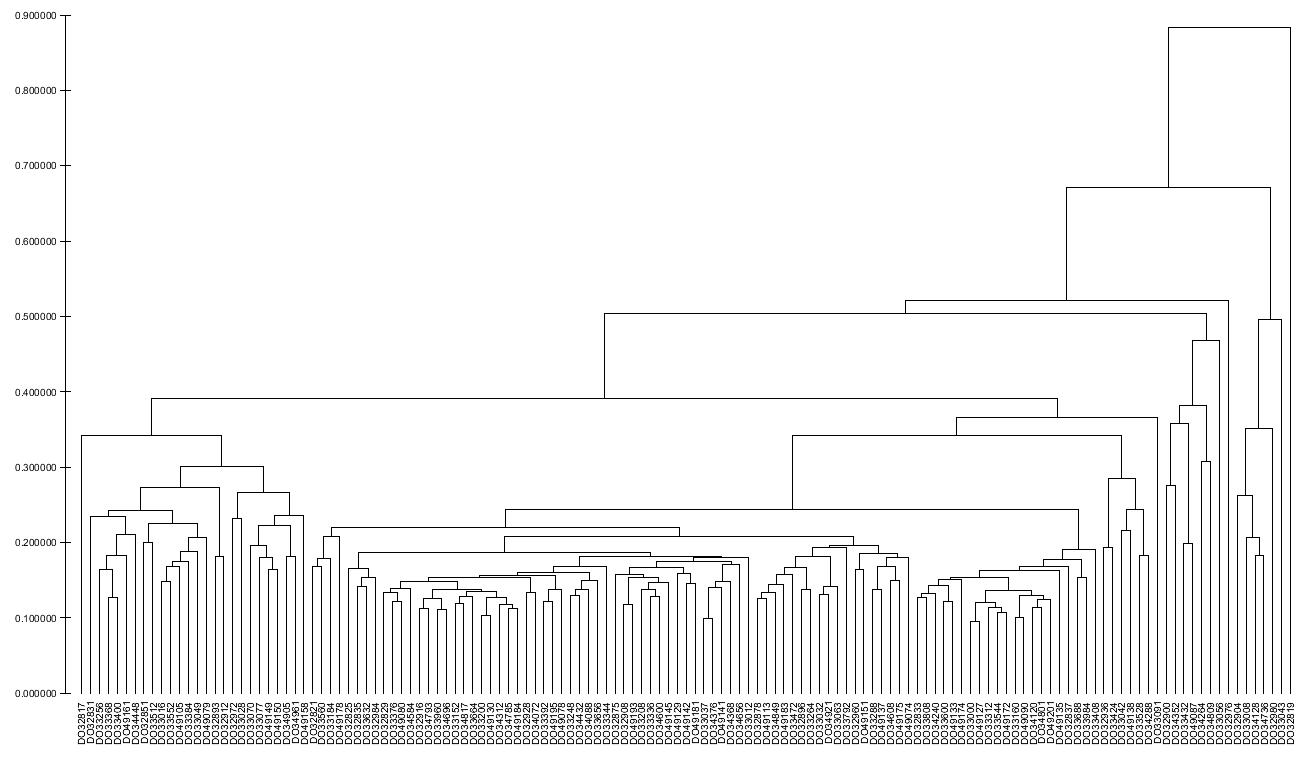


n) PAEN-AU


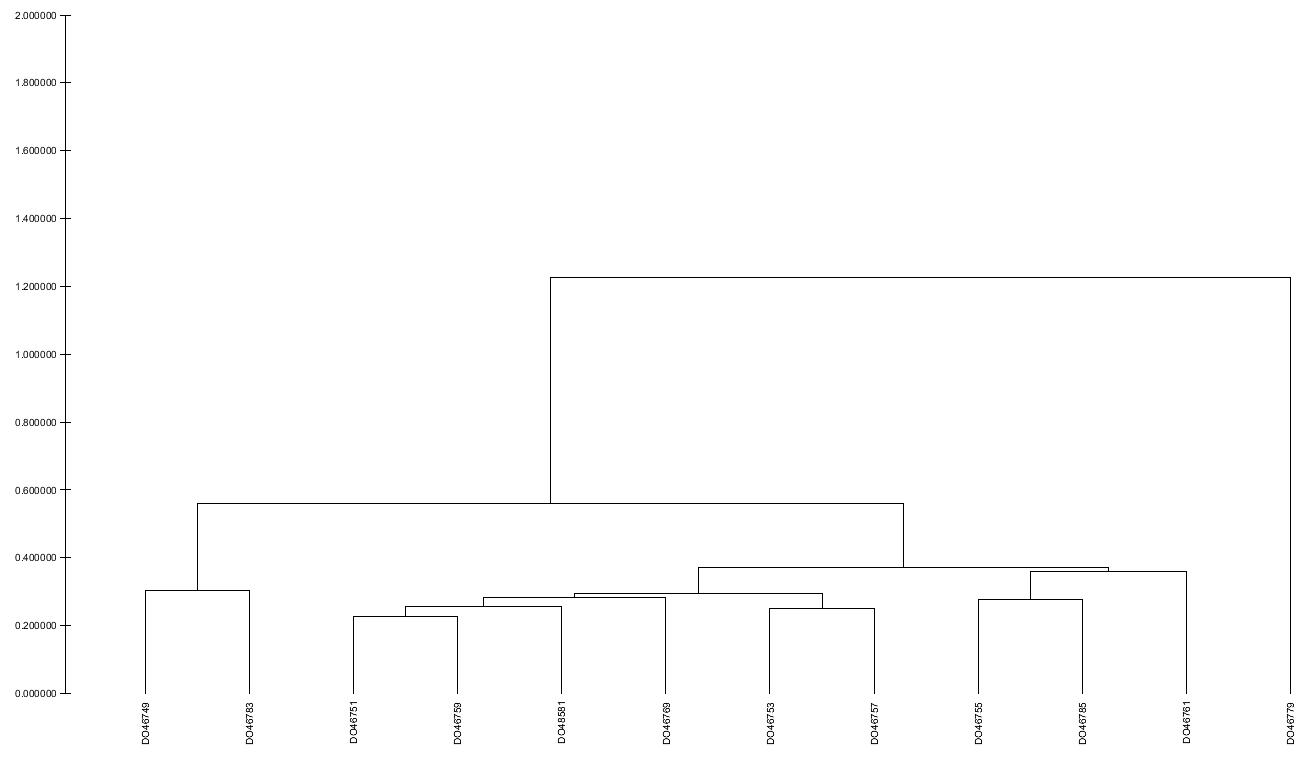


o) Pancreas


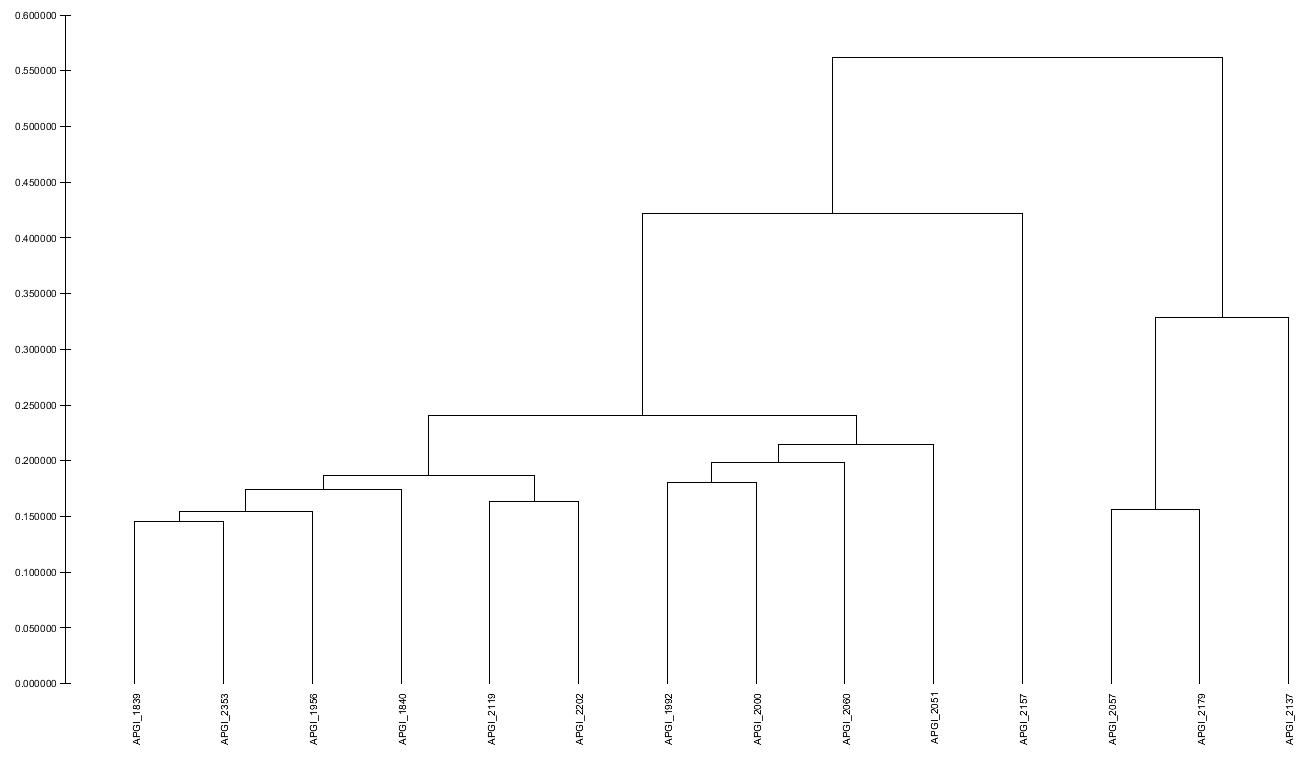


p) Medulloblastoma


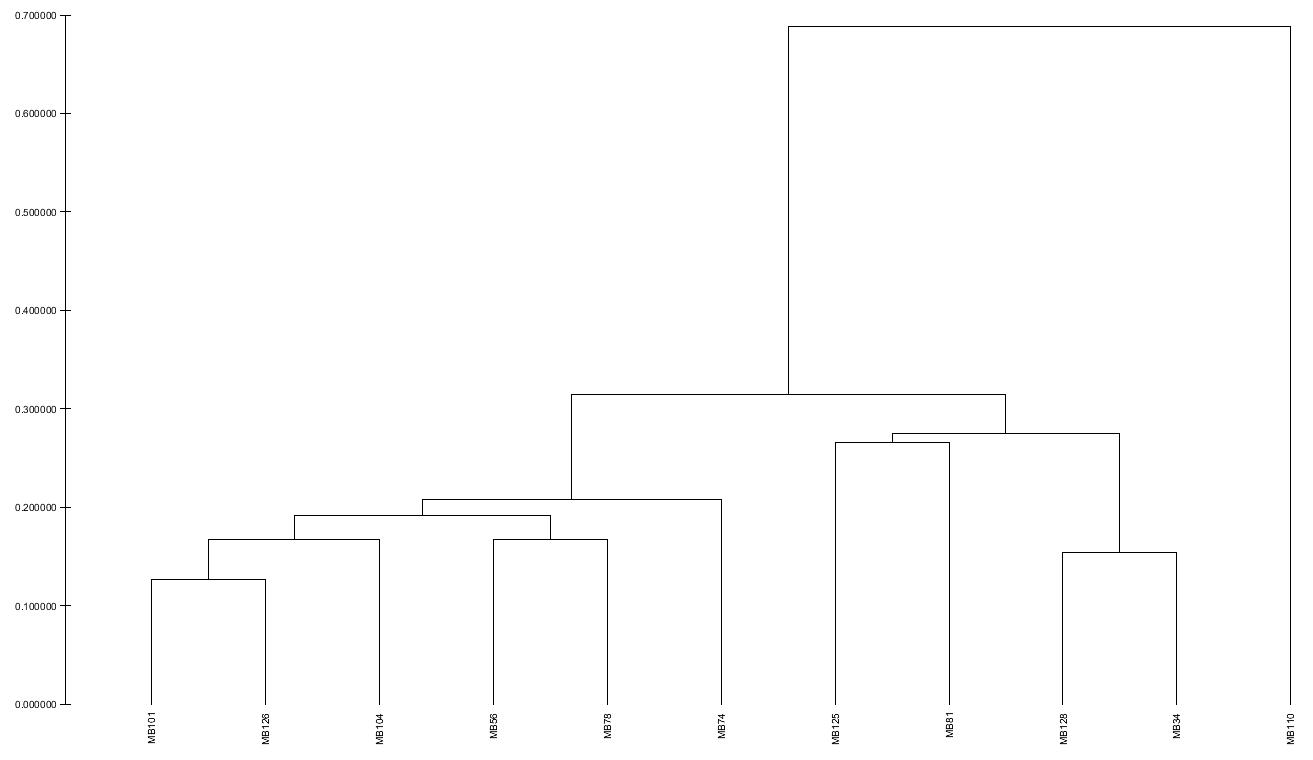


q) PBCA-DE


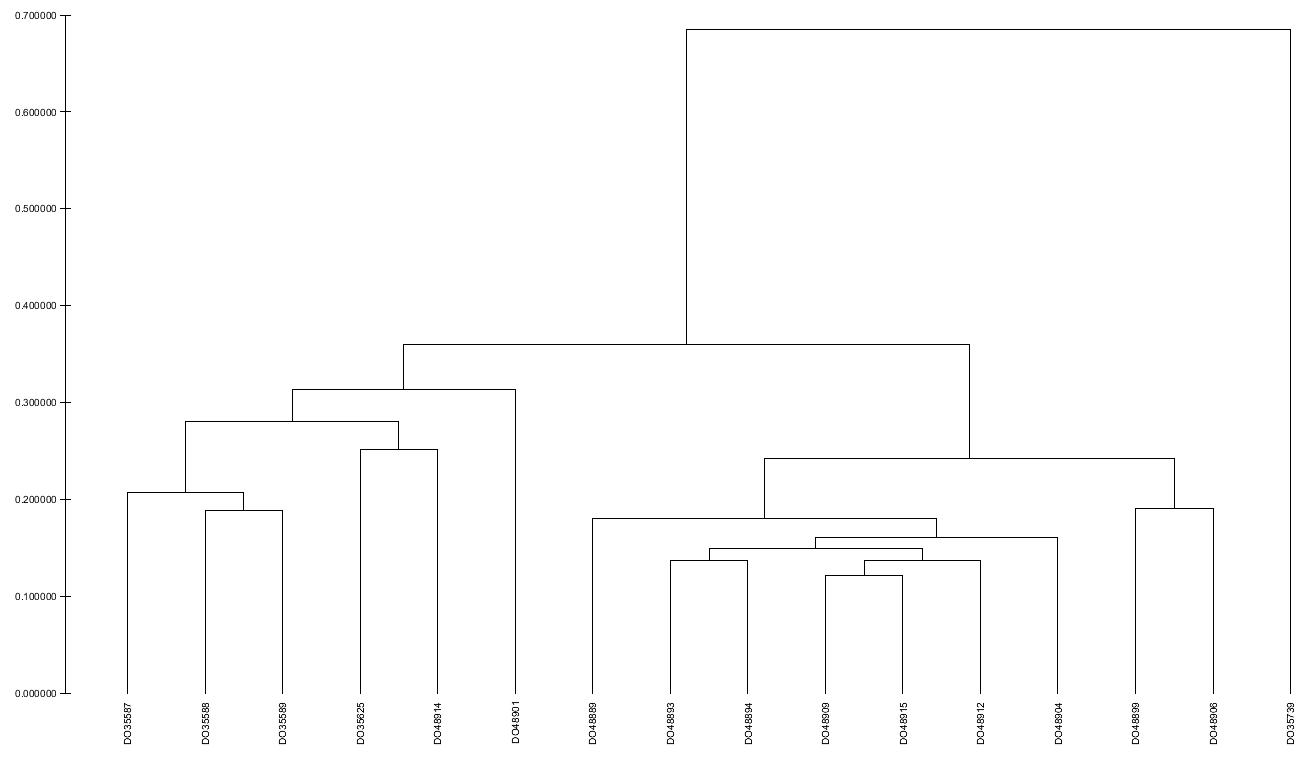


r) PRAD-UK


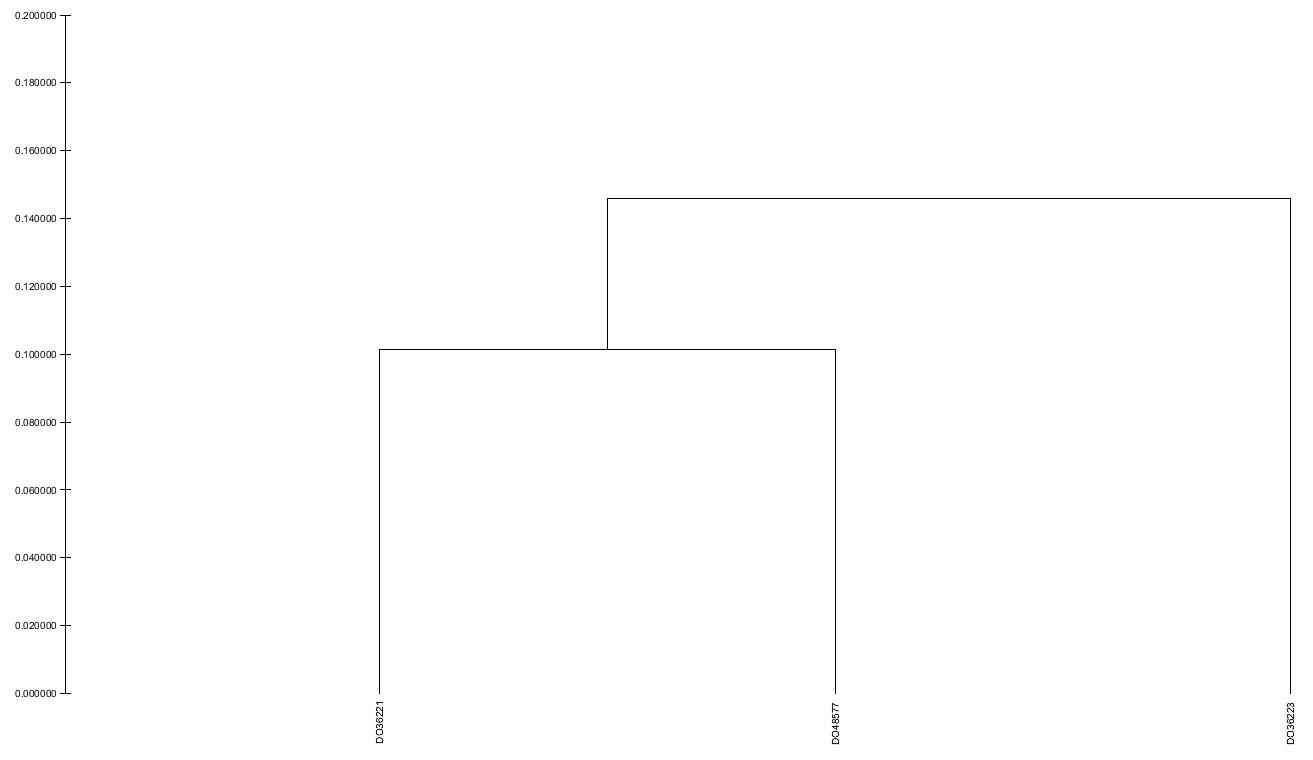


s) Prostate


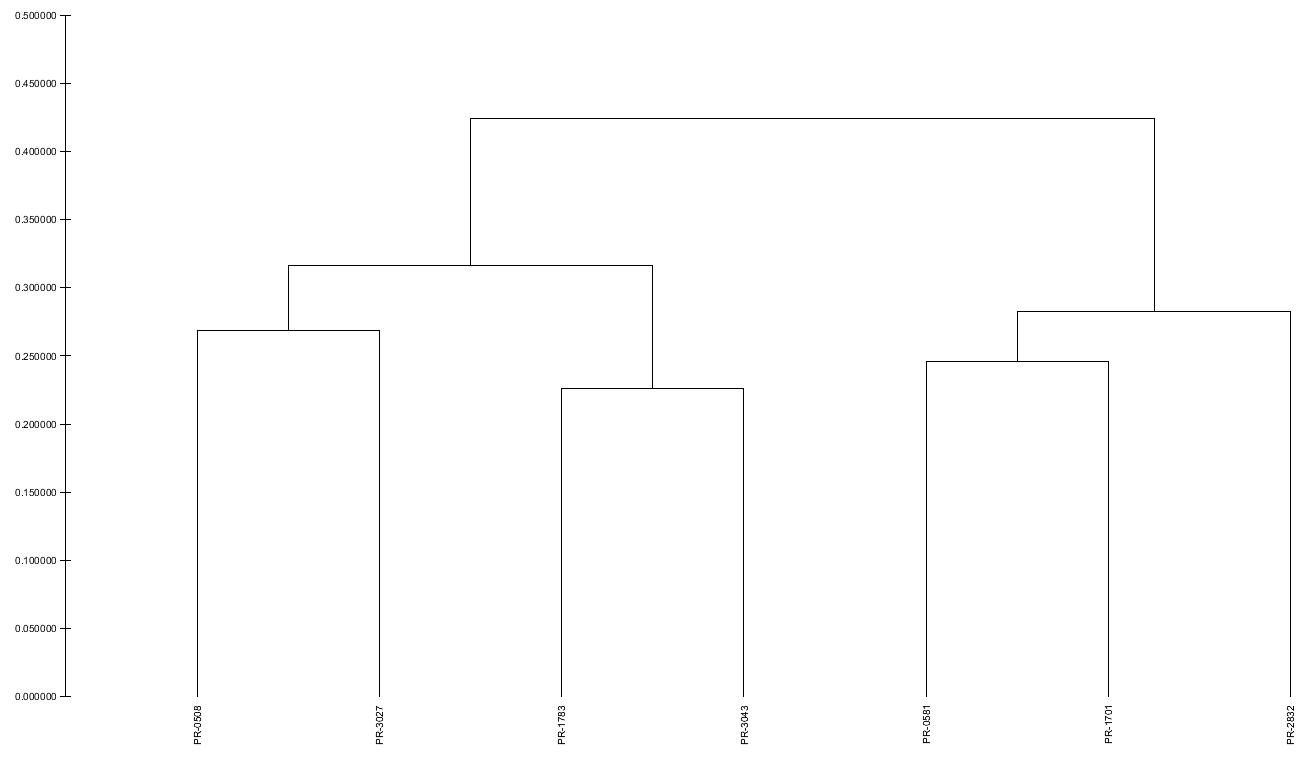


t) RECA-EU


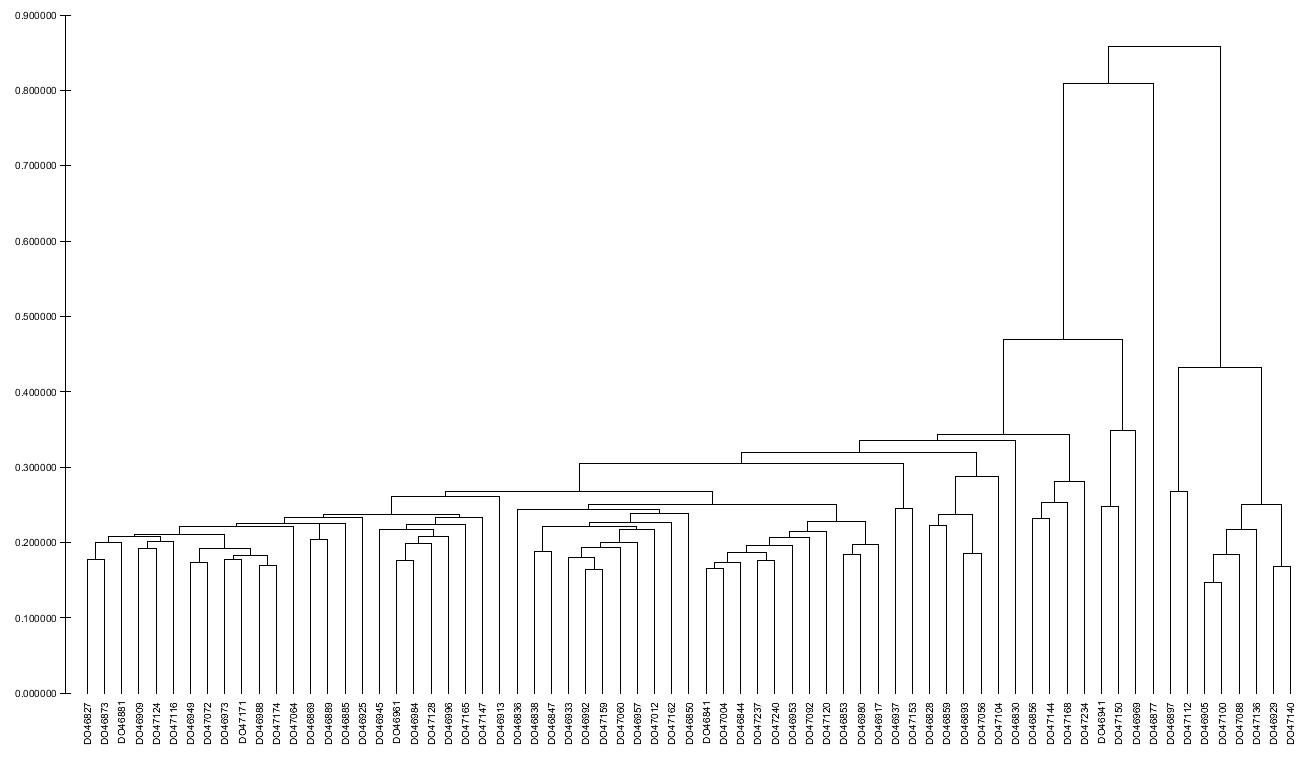


**Supplemental Figure 3**

| PD4020a |
| --- |
| 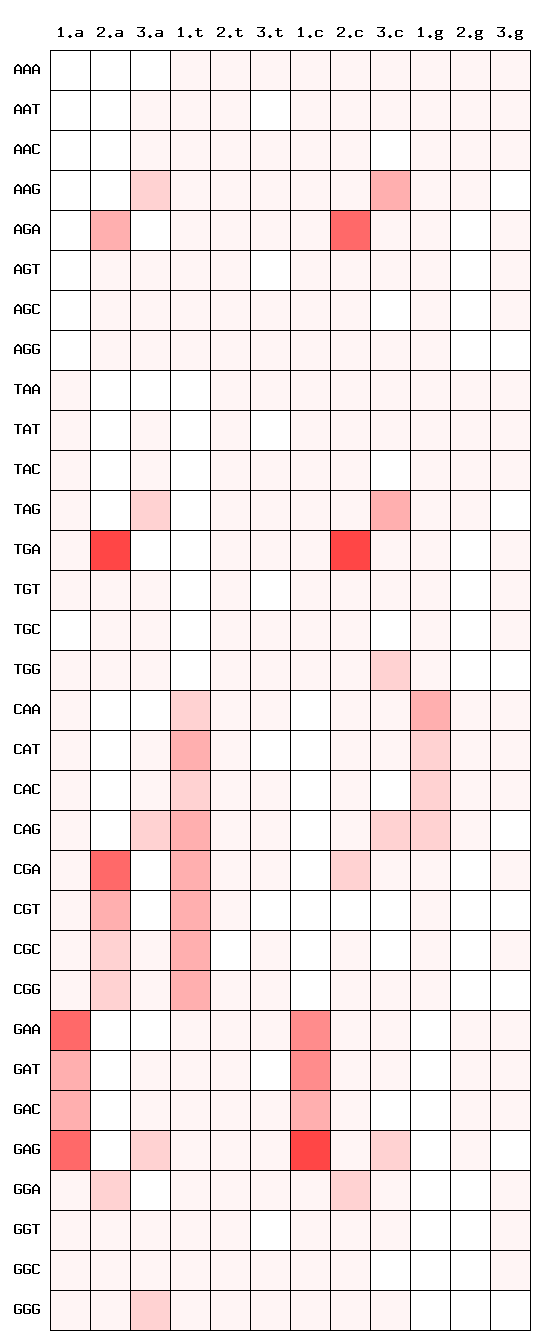 |
| \| Pattern \| Mutation \| Fraction \| \| --- \| --- \| --- \| \| TGA \| 2.a \| 0.054179 \| \| GAG \| 1.c \| 0.050260 \| \| TGA \| 2.c \| 0.046353 \| \| AGA \| 2.c \| 0.044194 \| \| GAG \| 1.a \| 0.037753 \| |

**Supplemental Figure 4**

| DO49532 | DO49535 |
| --- | --- |
| 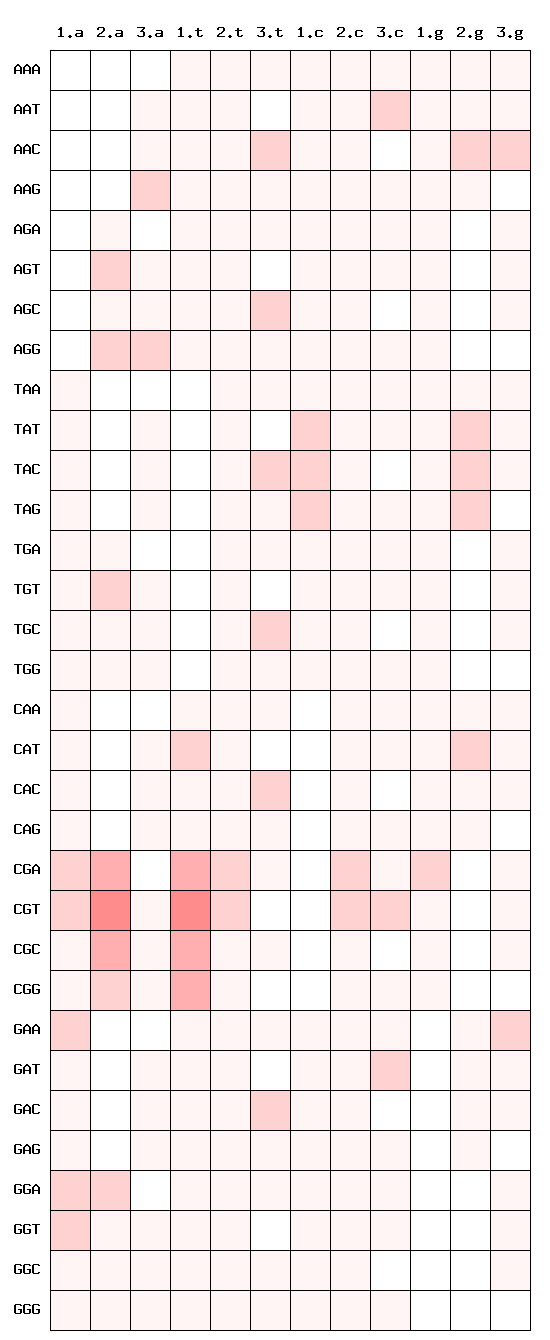 | 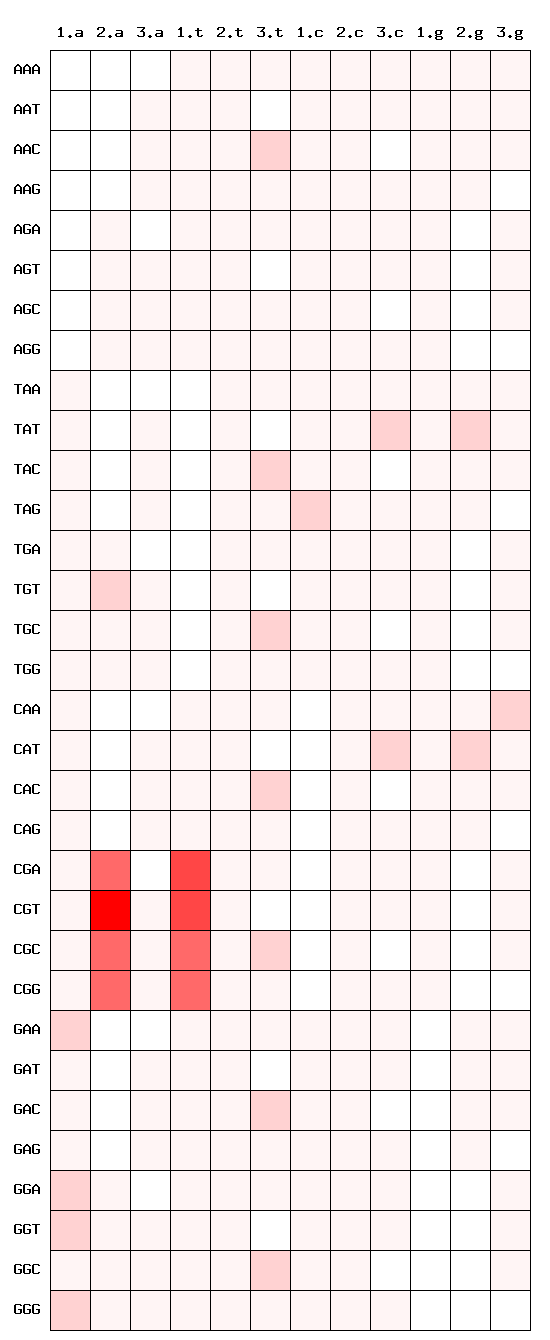 |
| \| Trinucleotide \| Mutation \| Fraction \| \| --- \| --- \| --- \| \| CGT \| 2.a \| 0.033042 \| \| CGT \| 1.t \| 0.028223 \| \| CGA \| 2.a \| 0.023530 \| \| CGA \| 1.t \| 0.021177 \| \| CGC \| 1.t \| 0.020193 \| | \| Trinucleotide \| Mutation \| Fraction \| \| --- \| --- \| --- \| \| CGT \| 2.a \| 0.068450 \| \| CGA \| 1.t \| 0.050520 \| \| CGT \| 1.t \| 0.047813 \| \| CGG \| 1.t \| 0.044610 \| \| CGC \| 1.t \| 0.042065 \| |

**Supplemental Figure 5**

| DO1003 | DO1010 |
| --- | --- |
| 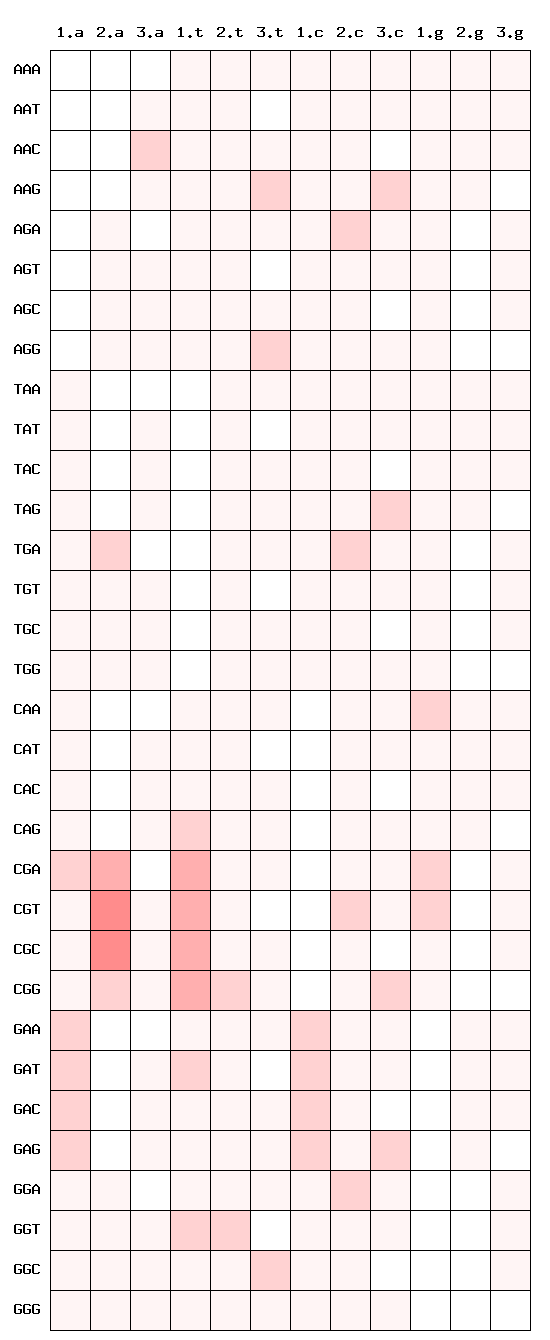 | 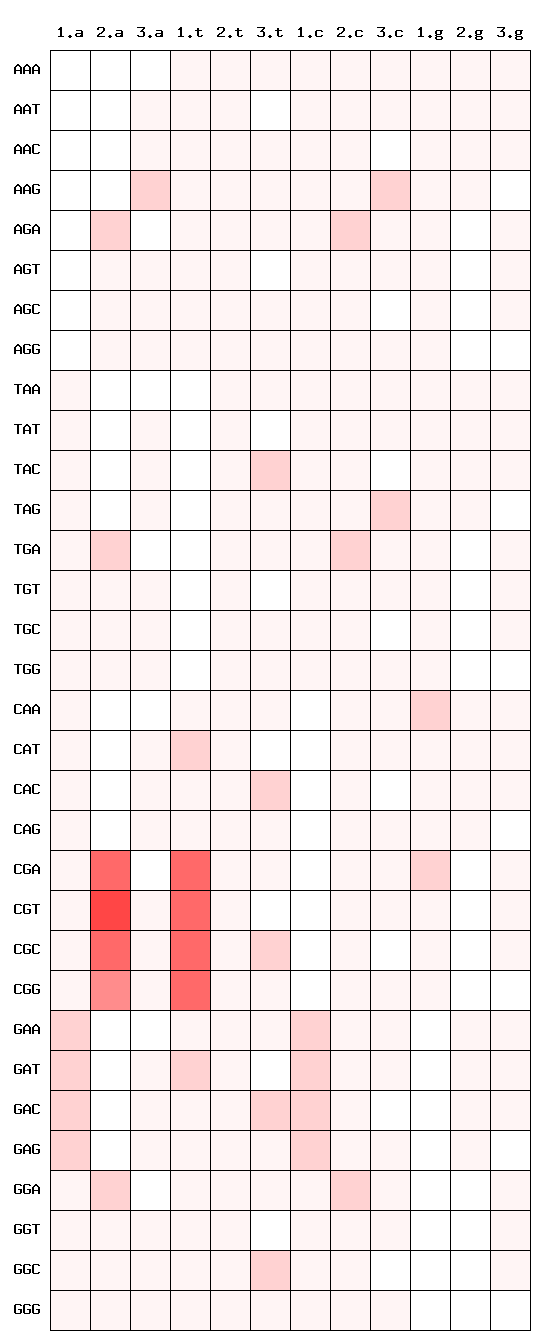 |
| \| Trinucleotide \| Mutation \| Fraction \| \| --- \| --- \| --- \| \| CGT \| 2.a \| 0.032481 \| \| CGC \| 2.a \| 0.026799 \| \| CGT \| 1.t \| 0.023955 \| \| CGC \| 1.t \| 0.023396 \| \| CGA \| 1.t \| 0.023131 \| | \| Trinucleotide \| Mutation \| Fraction \| \| --- \| --- \| --- \| \| CGT \| 2.a \| 0.050953 \| \| CGA \| 1.t \| 0.041083 \| \| CGC \| 2.a \| 0.040259 \| \| CGT \| 1.t \| 0.038934 \| \| CGC \| 1.t \| 0.037421 \| |

| DO1016 | DO1076 |
| --- | --- |
| 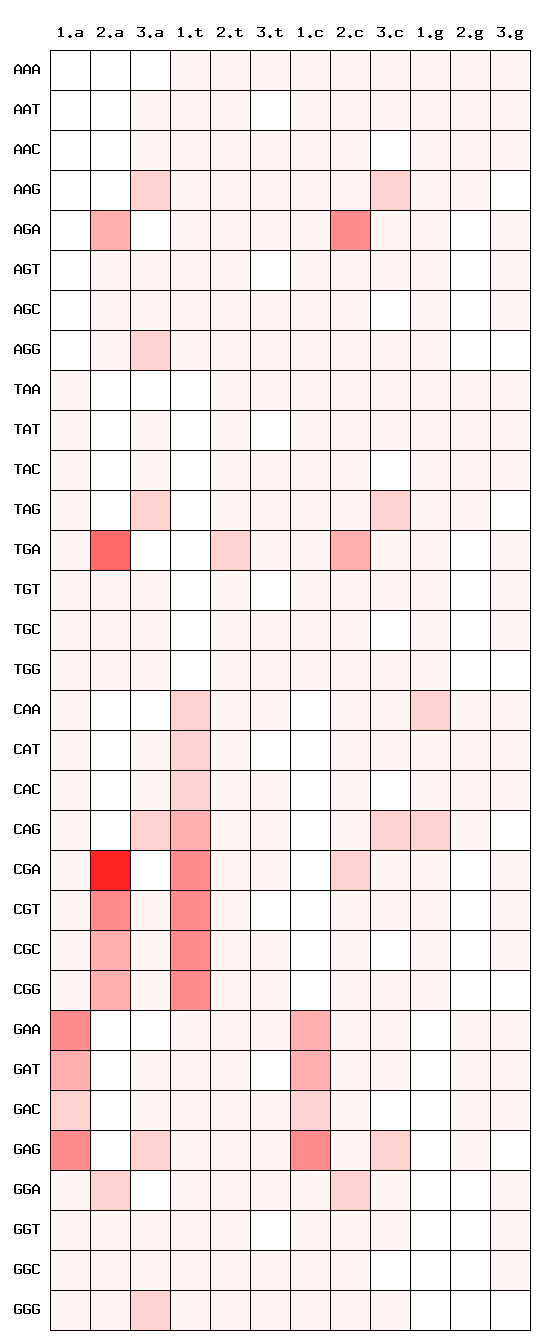 | 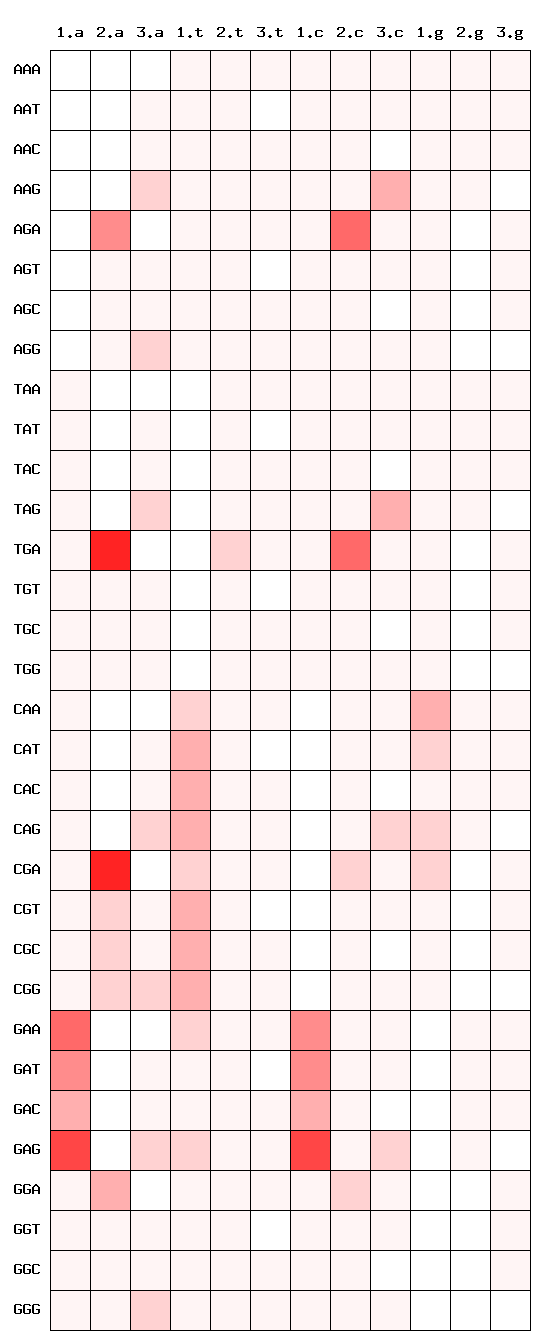 |
| \| Trinucleotide \| Mutation \| Fraction \| \| --- \| --- \| --- \| \| CGA \| 2.a \| 0.060676 \| \| TGA \| 2.a \| 0.041081 \| \| GAG \| 1.a \| 0.034629 \| \| CGA \| 1.t \| 0.033486 \| \| CGT \| 1.t \| 0.033157 \| | \| Trinucleotide \| Mutation \| Fraction \| \| --- \| --- \| --- \| \| TGA \| 2.a \| 0.062447 \| \| CGA \| 2.a \| 0.059465 \| \| GAG \| 1.a \| 0.049604 \| \| GAG \| 1.c \| 0.048431 \| \| GAA \| 1.a \| 0.042211 \| |

**Supplemental Figure 6**


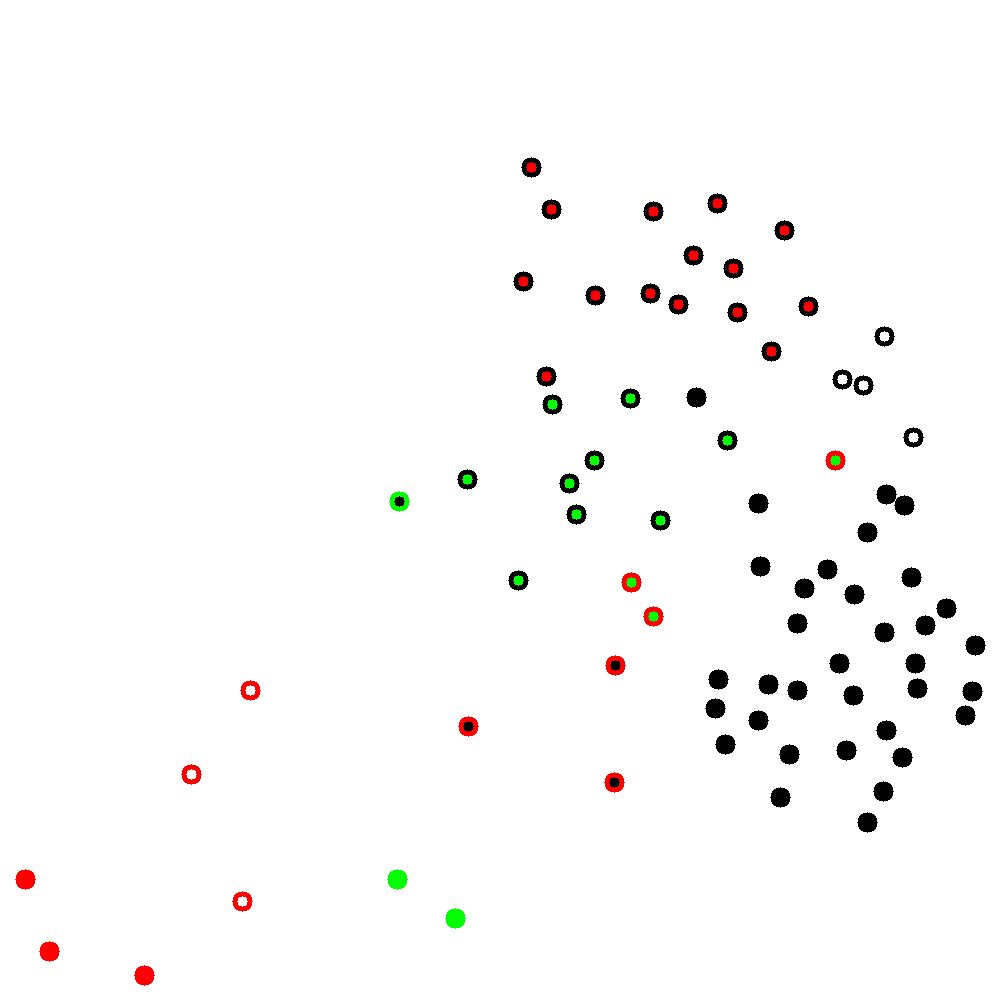


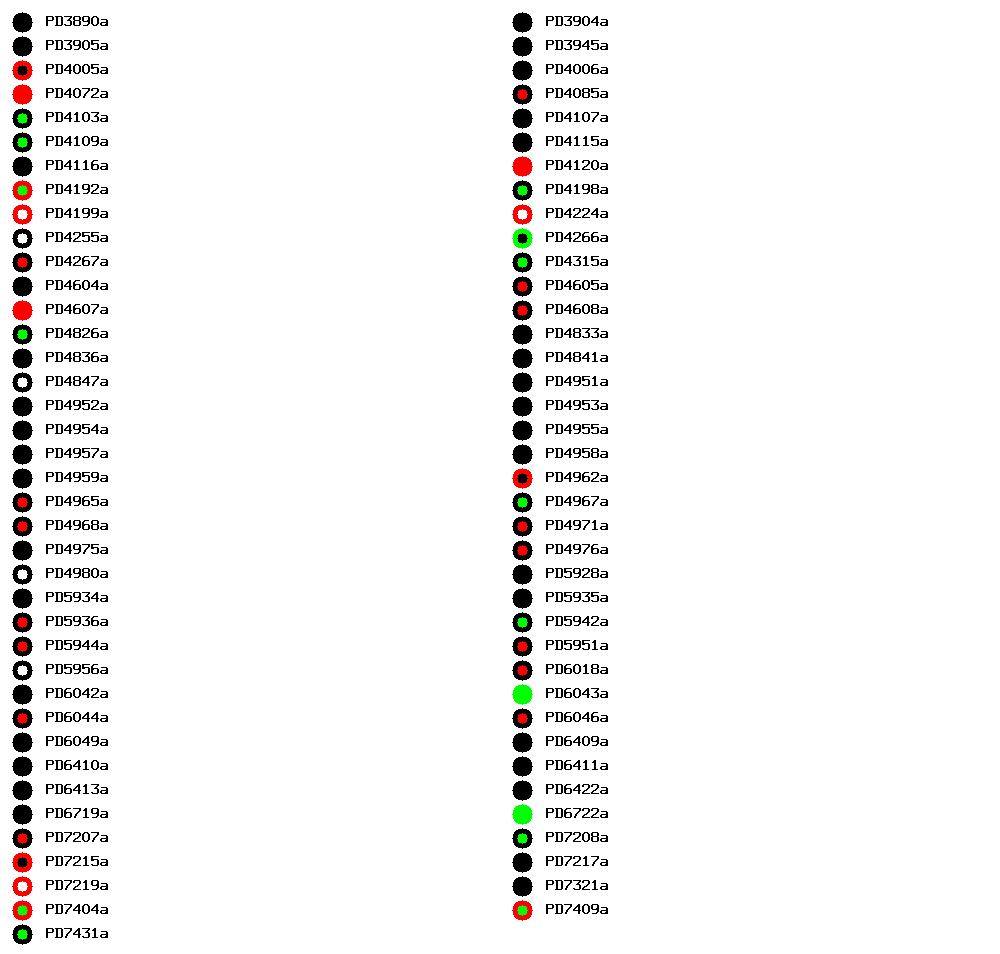


**Supplemental Figure 7**

| PD4266a | PD6722a | PD4005a |
| --- | --- | --- |
| 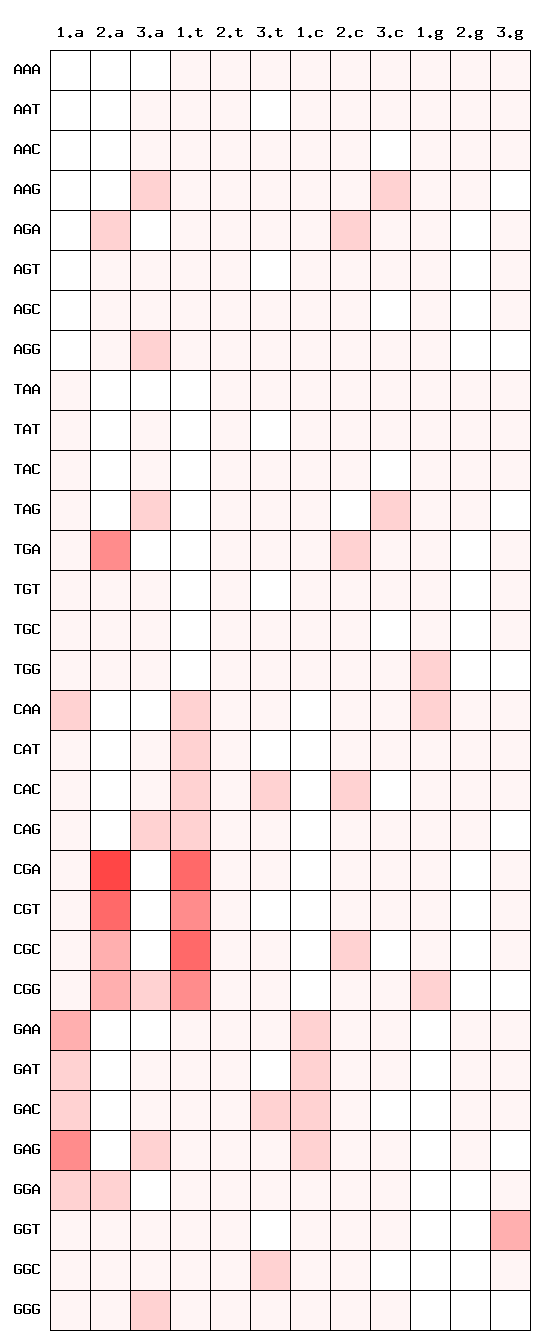 | 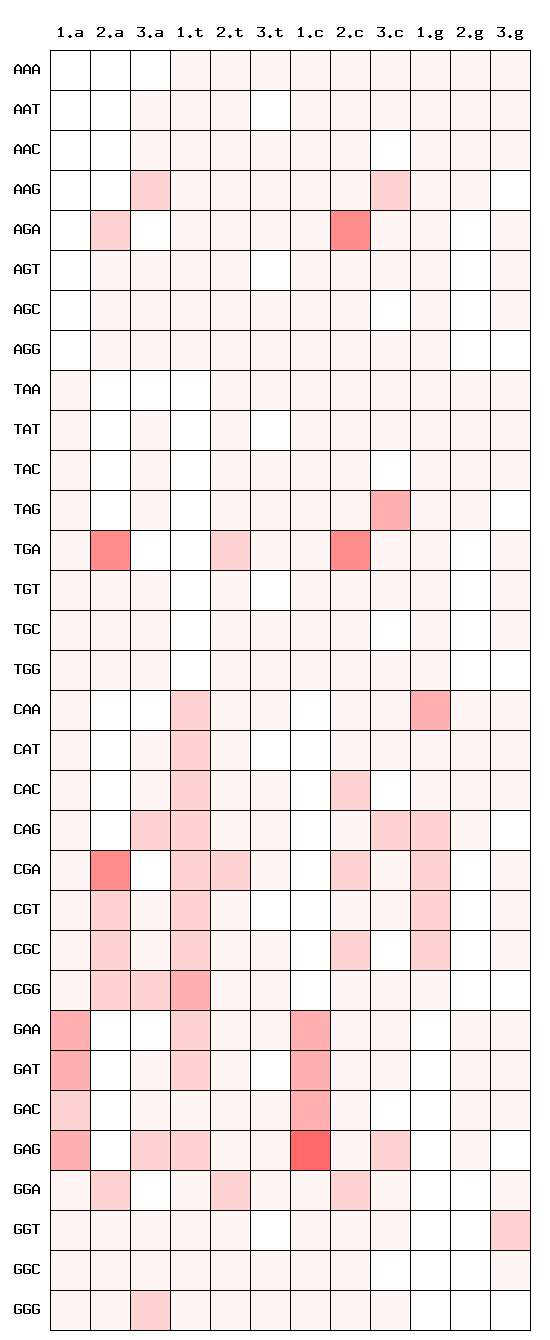 | 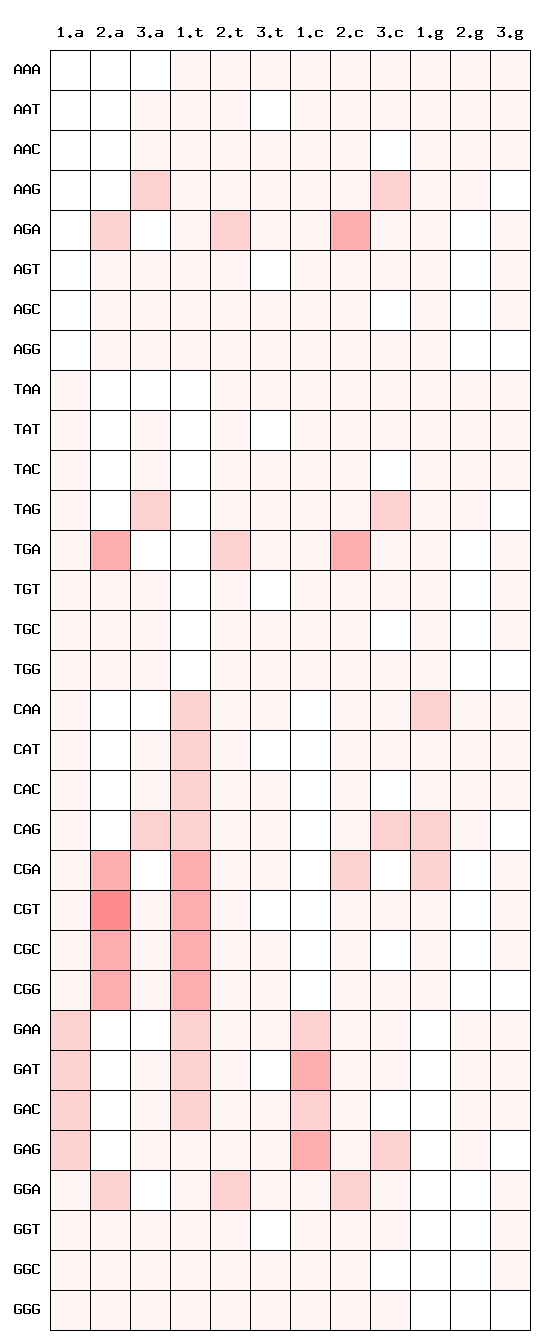 |
| \| Trinucleotide \| Mutation \| Fraction \| \| --- \| --- \| --- \| \| CGA \| 2.a \| 0.050594 \| \| CGT \| 2.a \| 0.043779 \| \| CGC \| 1.t \| 0.038004 \| \| CGA \| 1.t \| 0.037767 \| \| CGT \| 1.t \| 0.034397 \| | \| Trinucleotide \| Mutation \| Fraction \| \| --- \| --- \| --- \| \| GAG \| 1.c \| 0.036496 \| \| AGA \| 2.c \| 0.033526 \| \| TGA \| 2.c \| 0.031206 \| \| TGA \| 2.a \| 0.029449 \| \| CGA \| 2.a \| 0.027404 \| | \| Trinucleotide \| Mutation \| Fraction \| \| --- \| --- \| --- \| \| CGT \| 2.a \| 0.025595 \| \| AGA \| 2.c \| 0.022945 \| \| GAG \| 1.c \| 0.021122 \| \| CGG \| 1.t \| 0.020703 \| \| TGA \| 2.a \| 0.018765 \| |

| PD4120a | PD7404a | PD4224a |
| --- | --- | --- |
| 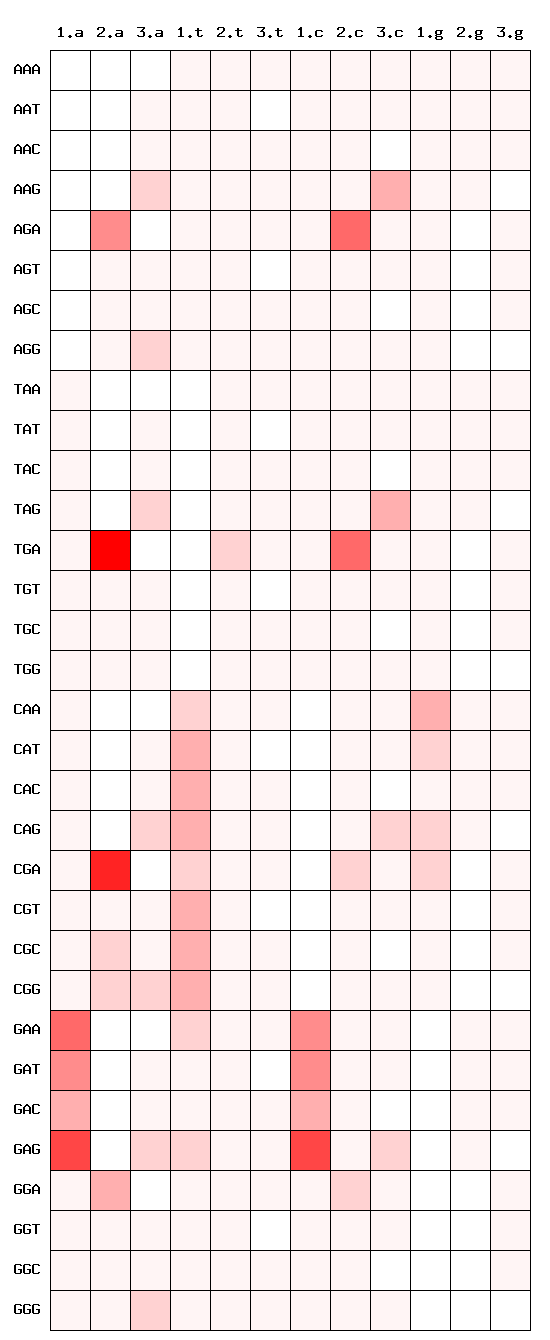 | 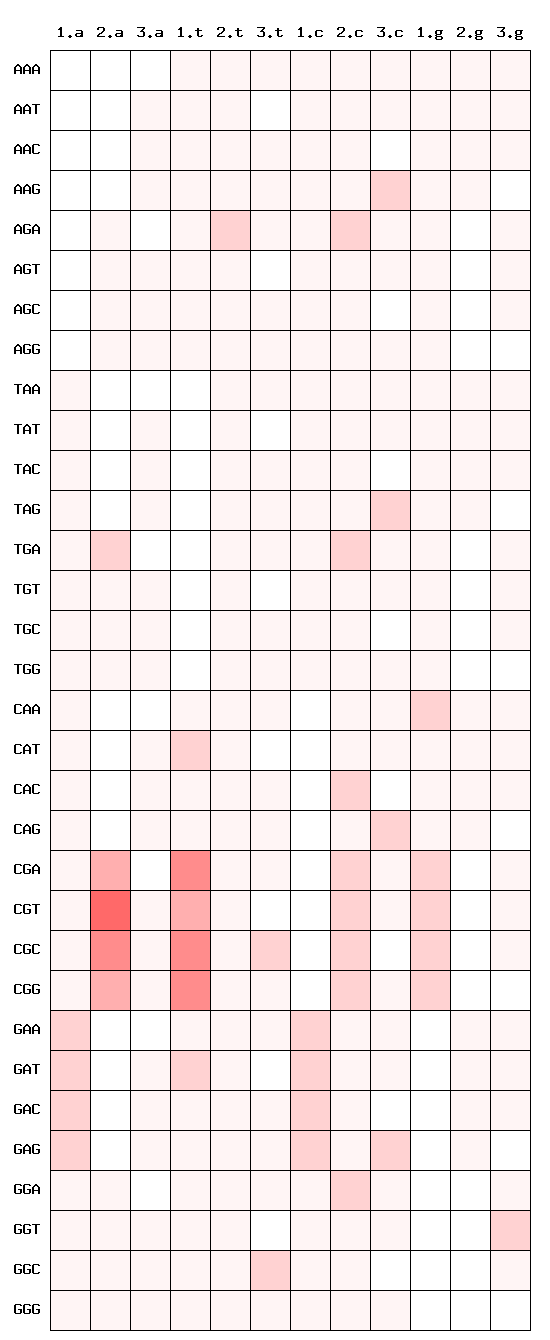 | 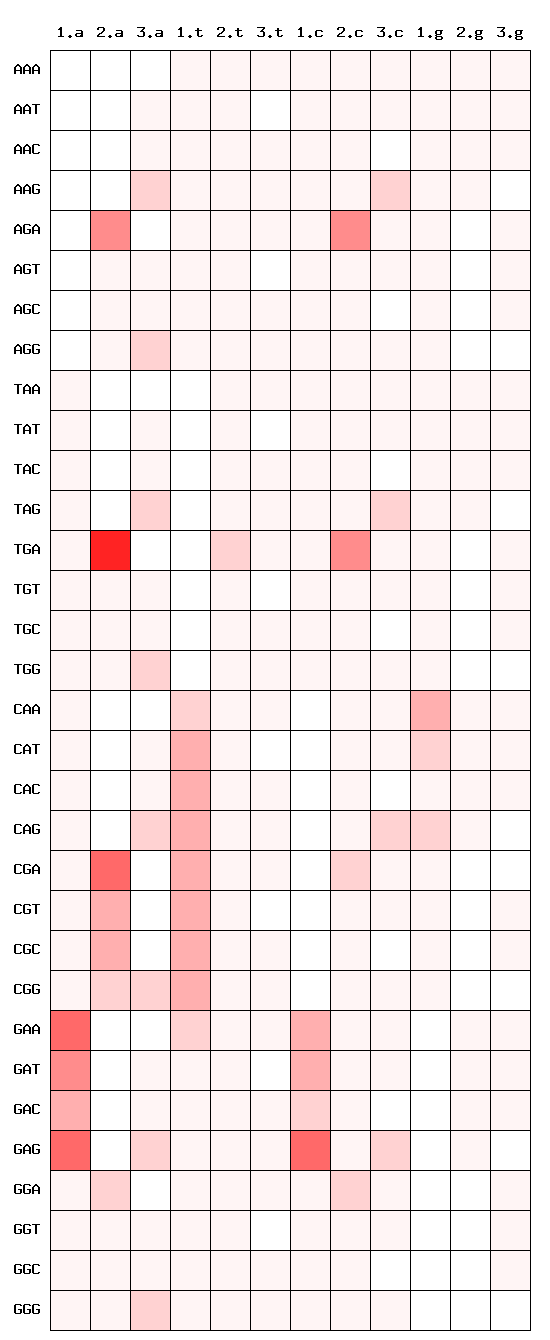 |
| \| Trinucleotide \| Mutation \| Fraction \| \| --- \| --- \| --- \| \| TGA \| 2.a \| 0.066098 \| \| CGA \| 2.a \| 0.056226 \| \| GAG \| 1.a \| 0.052043 \| \| GAG \| 1.c \| 0.050910 \| \| GAA \| 1.a \| 0.044247 \| | \| Trinucleotide \| Mutation \| Fraction \| \| --- \| --- \| --- \| \| CGT \| 2.a \| 0.036338 \| \| CGC \| 2.a \| 0.033667 \| \| CGG \| 1.t \| 0.031135 \| \| CGC \| 1.t \| 0.031110 \| \| CGA \| 1.t \| 0.029353 \| | \| Trinucleotide \| Mutation \| Fraction \| \| --- \| --- \| --- \| \| TGA \| 2.a \| 0.060264 \| \| GAG \| 1.a \| 0.043799 \| \| CGA \| 2.a \| 0.041747 \| \| GAA \| 1.a \| 0.040316 \| \| GAG \| 1.c \| 0.035191 \| |

| PD4980a | PD4315a | PD4965a |
| --- | --- | --- |
| 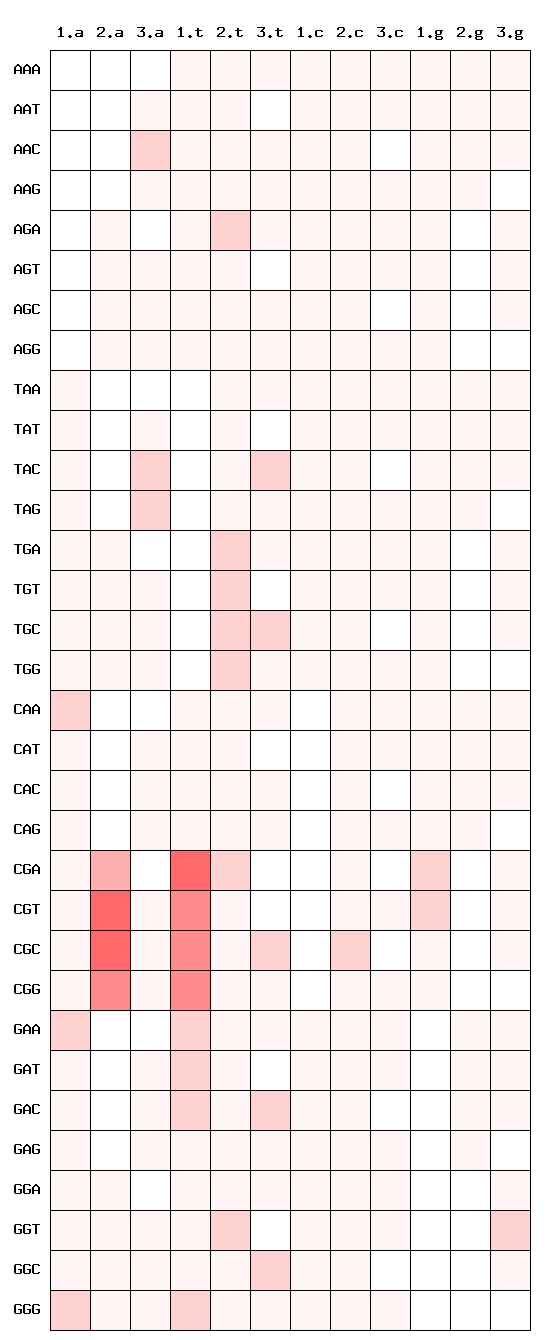 | 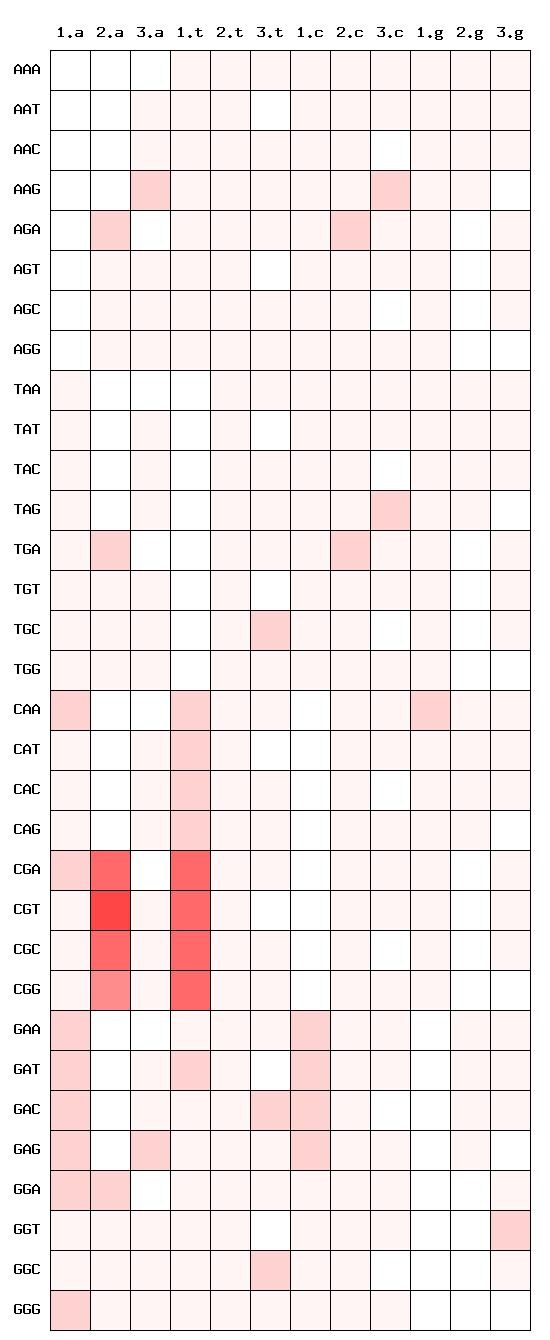 | 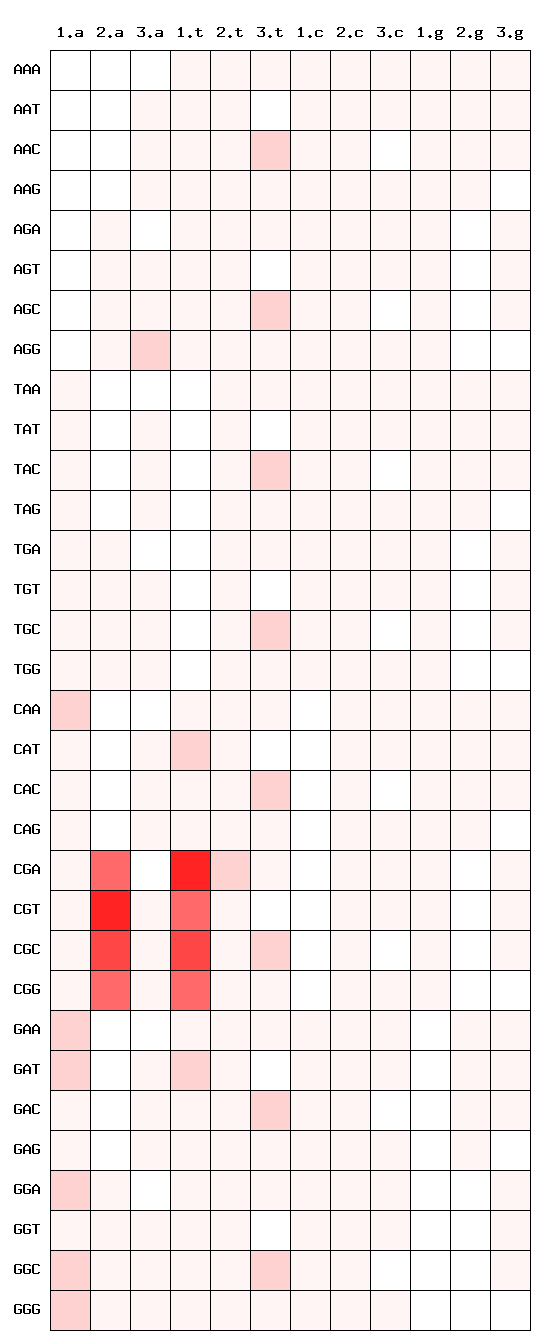 |
| \| Trinucleotide \| Mutation \| Fraction \| \| --- \| --- \| --- \| \| CGC \| 2.a \| 0.044982 \| \| CGA \| 1.t \| 0.041998 \| \| CGT \| 2.a \| 0.041720 \| \| CGG \| 1.t \| 0.034054 \| \| CGC \| 1.t \| 0.033525 \| | \| Trinucleotide \| Mutation \| Fraction \| \| --- \| --- \| --- \| \| CGT \| 2.a \| 0.045967 \| \| CGA \| 1.t \| 0.042852 \| \| CGA \| 2.a \| 0.041424 \| \| CGG \| 1.t \| 0.038155 \| \| CGC \| 1.t \| 0.036338 \| | \| Trinucleotide \| Mutation \| Fraction \| \| --- \| --- \| --- \| \| CGA \| 1.t \| 0.059315 \| \| CGT \| 2.a \| 0.058826 \| \| CGC \| 1.t \| 0.053995 \| \| CGC \| 2.a \| 0.049632 \| \| CGG \| 2.a \| 0.043768 \| |

| PD4952a |
| --- |
| 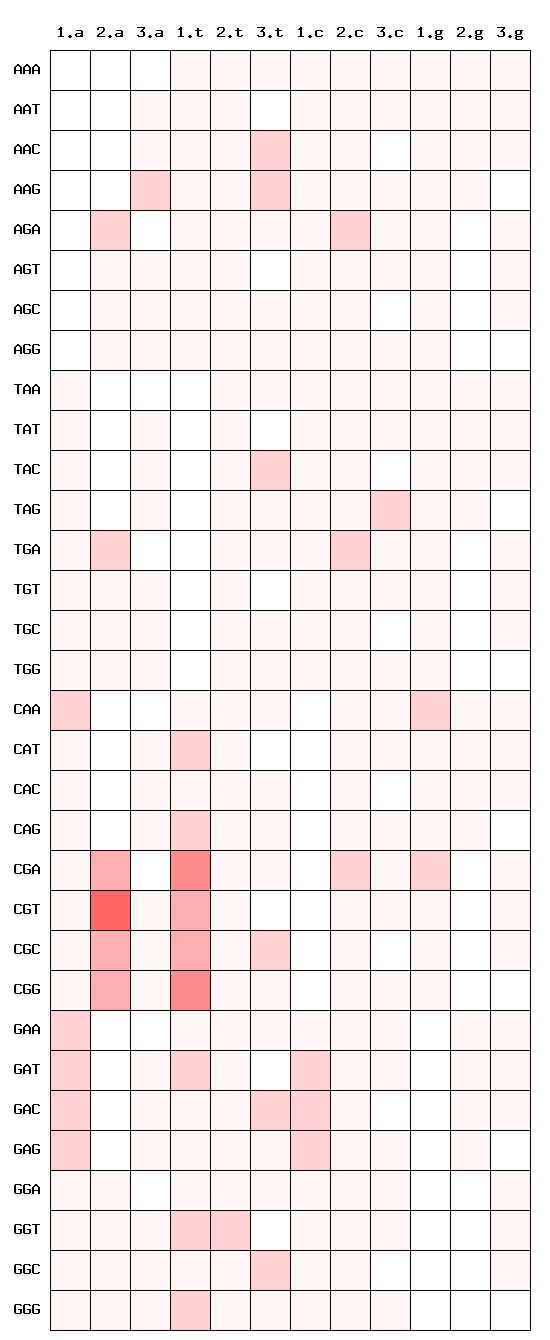 |
| \| Trinucleotide \| Mutation \| Fraction \| \| --- \| --- \| --- \| \| CGT \| 2.a \| 0.035435 \| \| CGA \| 1.t \| 0.026746 \| \| CGG \| 1.t \| 0.025006 \| \| CGC \| 1.t \| 0.024437 \| \| CGC \| 2.a \| 0.023967 \| |

**Supplemental Figure 8**

| DO10842 | DO10840 |
| --- | --- |
| 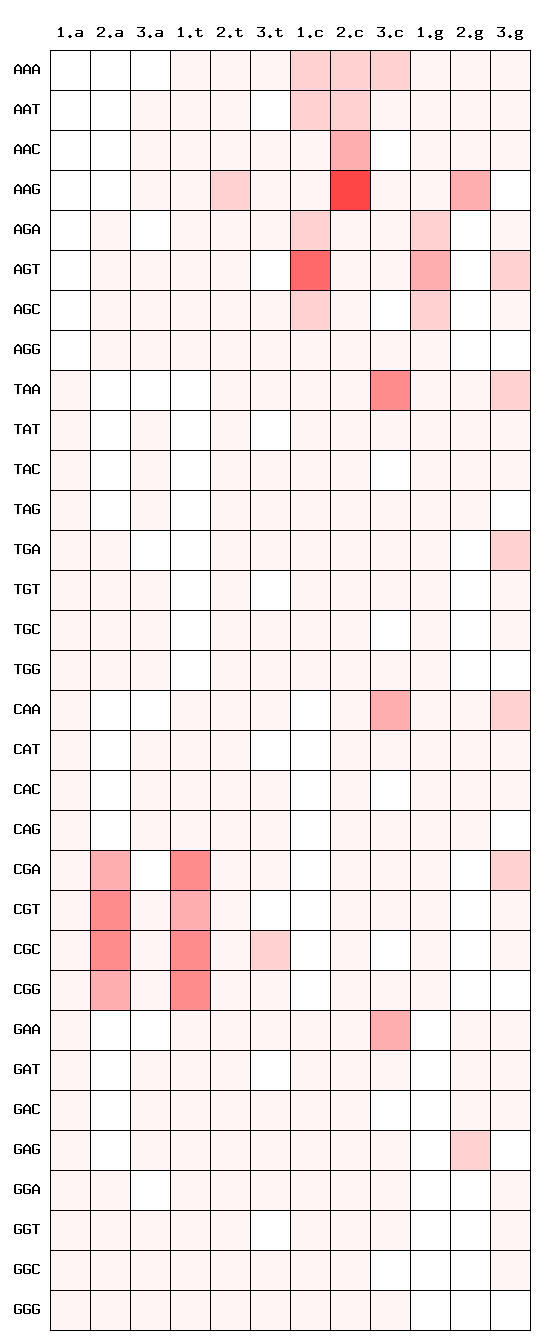 | 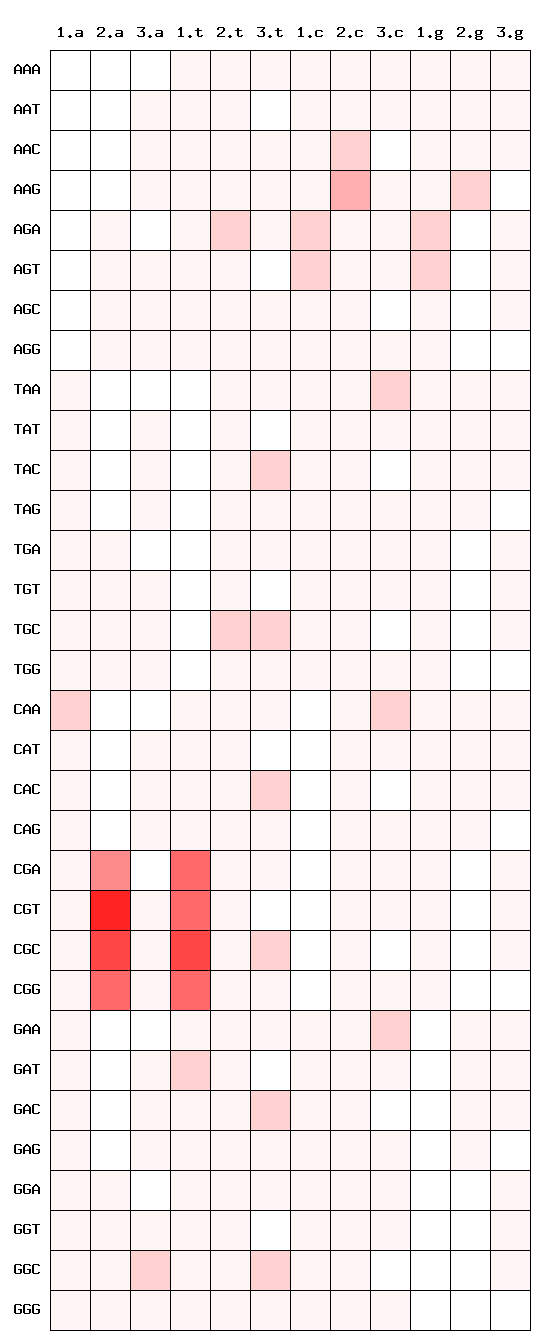 |
| \| Trinucleotide \| Mutation \| Fraction \| \| --- \| --- \| --- \| \| AAG \| 2.c \| 0.052602 \| \| AGT \| 1.c \| 0.040608 \| \| CGT \| 2.a \| 0.030900 \| \| TAA \| 3.c \| 0.029476 \| \| CGC \| 1.t \| 0.029353 \| | \| Trinucleotide \| Mutation \| Fraction \| \| --- \| --- \| --- \| \| CGT \| 2.a \| 0.058823 \| \| CGC \| 1.t \| 0.052175 \| \| CGC \| 2.a \| 0.049357 \| \| CGA \| 1.t \| 0.044386 \| \| CGG \| 1.t \| 0.044080 \| |

| DO10843 | DO10853 |
| --- | --- |
| 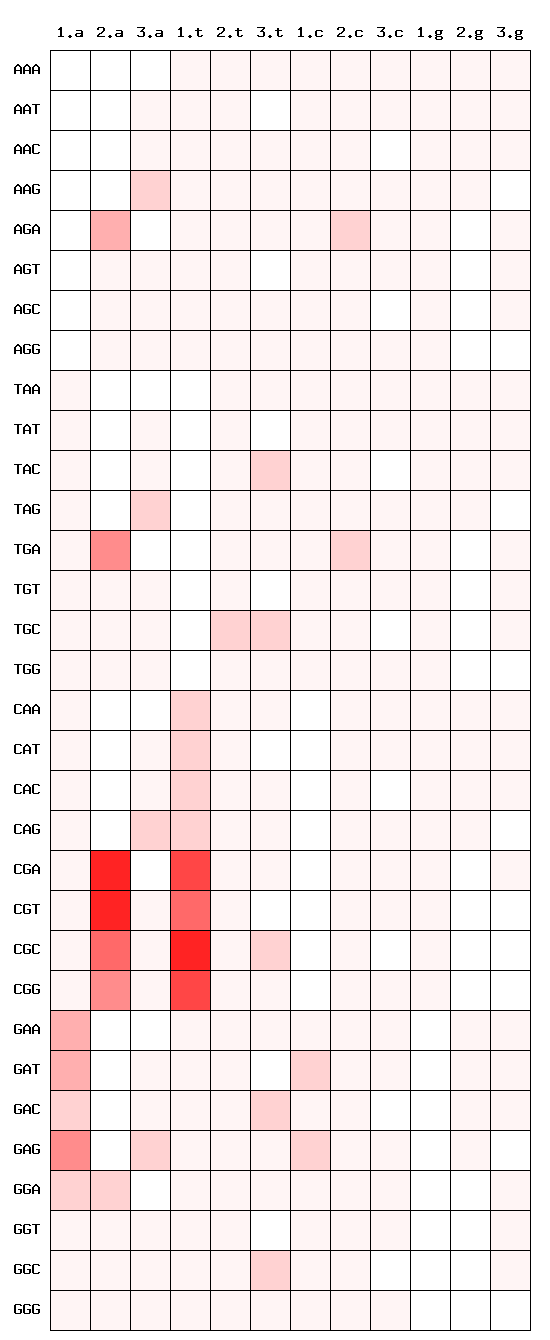 | 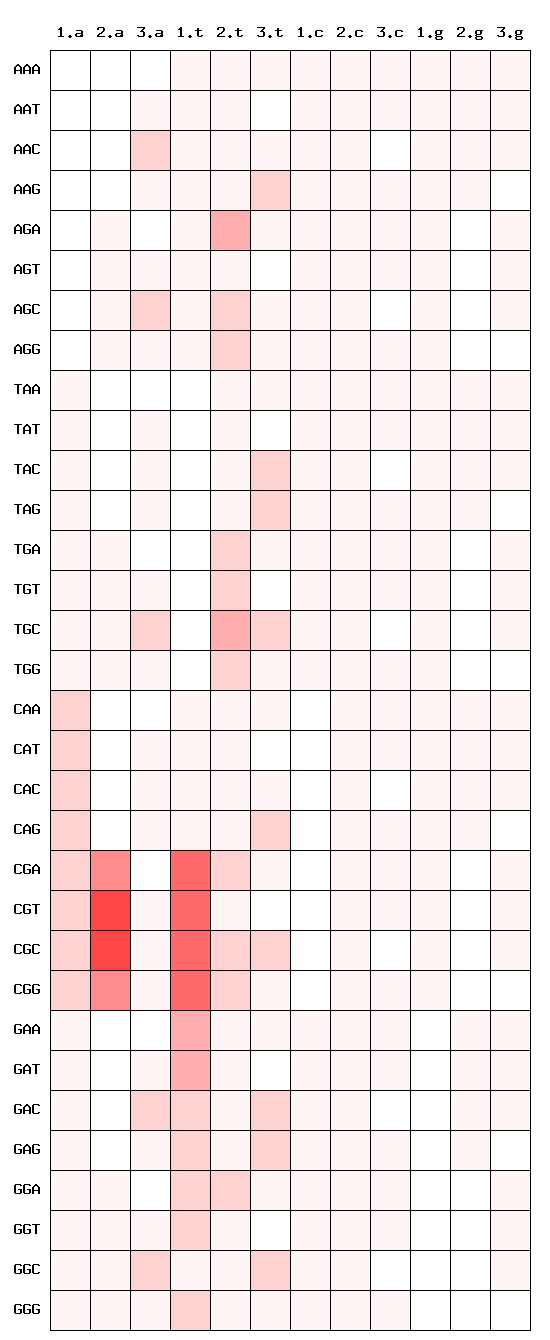 |
| \| Trinucleotide \| Mutation \| Fraction \| \| --- \| --- \| --- \| \| CGA \| 2.a \| 0.064590 \| \| CGT \| 2.a \| 0.062807 \| \| CGC \| 1.t \| 0.057872 \| \| CGA \| 1.t \| 0.052479 \| \| CGG \| 1.t \| 0.048772 \| | \| Trinucleotide \| Mutation \| Fraction \| \| --- \| --- \| --- \| \| CGT \| 2.a \| 0.051324 \| \| CGC \| 2.a \| 0.050335 \| \| CGG \| 1.t \| 0.042910 \| \| CGC \| 1.t \| 0.041946 \| \| CGA \| 1.t \| 0.040341 \| |

**Supplemental Figure 9**

| DO45031 | DO45069 | DO45061 |
| --- | --- | --- |
| 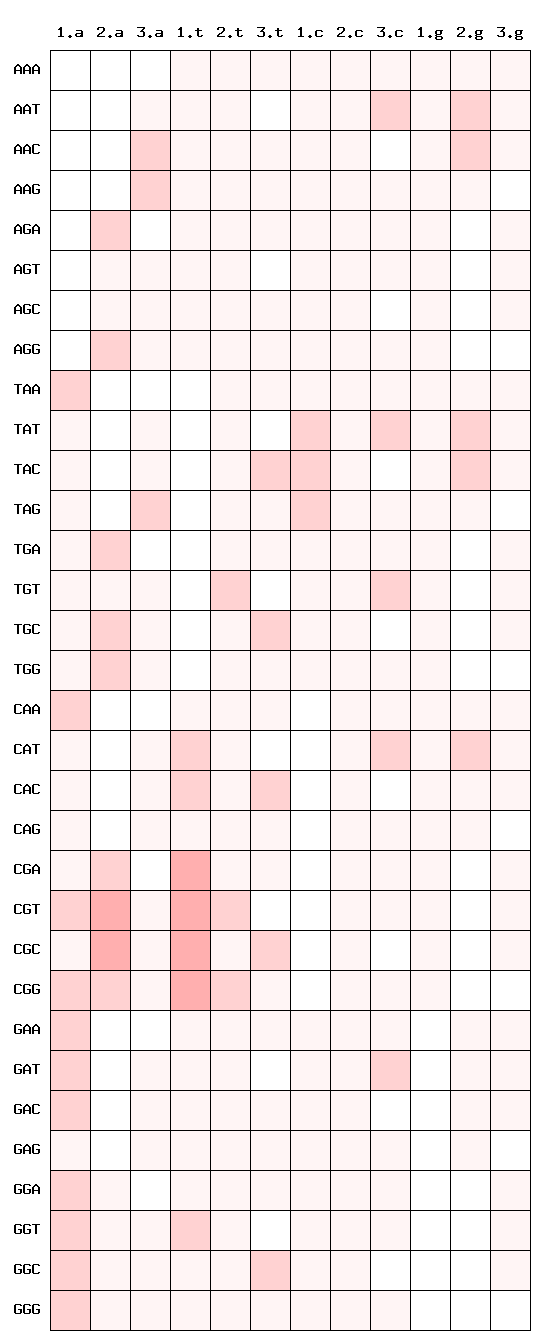 | 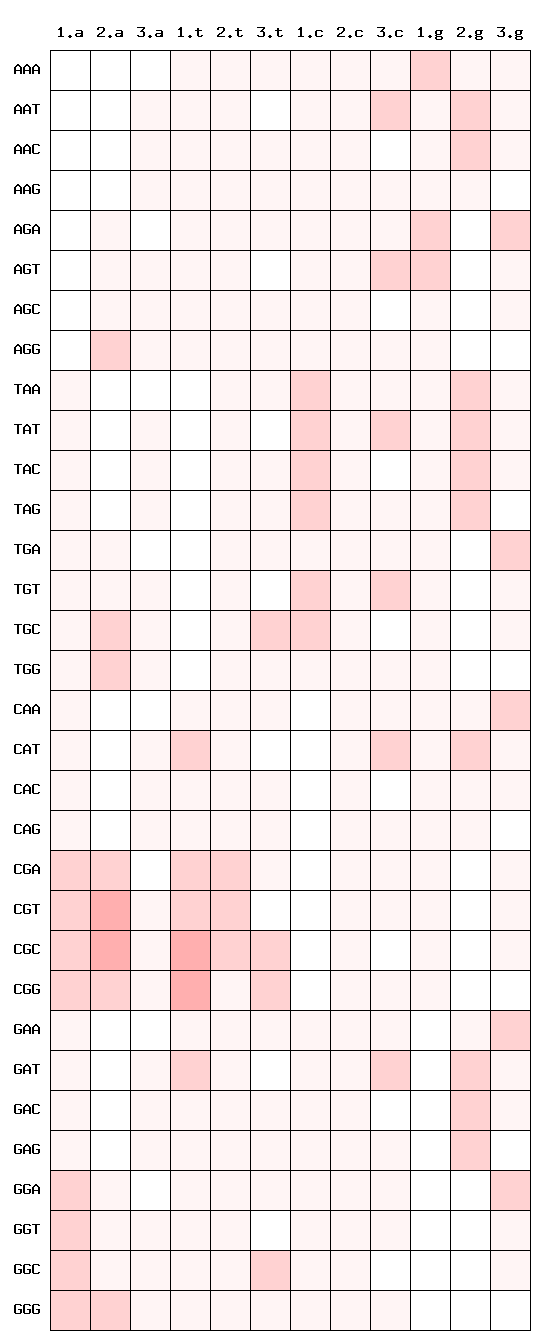 | 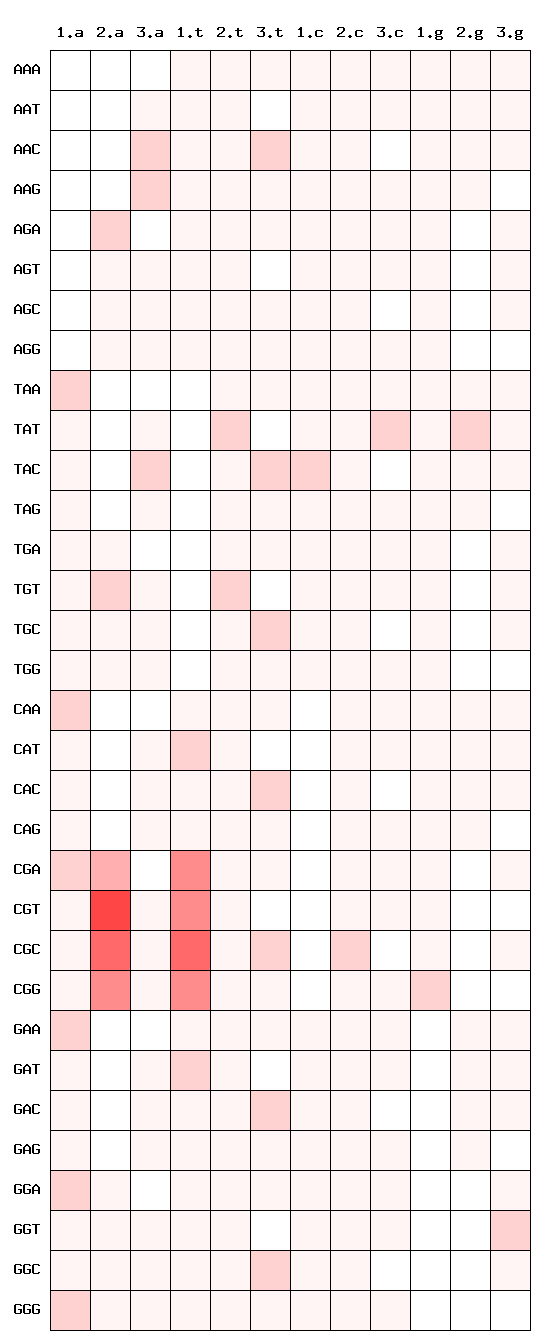 |
| \| Trinucleotide \| Mutation \| Fraction \| \| --- \| --- \| --- \| \| CGT \| 2.a \| 0.023786 \| \| CGC \| 2.a \| 0.021881 \| \| CGC \| 1.t \| 0.018842 \| \| CGA \| 1.t \| 0.018068 \| \| CGG \| 1.t \| 0.017832 \| | \| Trinucleotide \| Mutation \| Fraction \| \| --- \| --- \| --- \| \| CGC \| 2.a \| 0.017683 \| \| CGC \| 1.t \| 0.017040 \| \| CGT \| 2.a \| 0.017032 \| \| CGG \| 1.t \| 0.015675 \| \| CGA \| 1.t \| 0.014336 \| | \| Trinucleotide \| Mutation \| Fraction \| \| --- \| --- \| --- \| \| CGT \| 2.a \| 0.045608 \| \| CGC \| 1.t \| 0.038753 \| \| CGC \| 2.a \| 0.036496 \| \| CGA \| 1.t \| 0.032326 \| \| CGG \| 1.t \| 0.032141 \| |

| DO45041 | DO45047 |
| --- | --- |
| 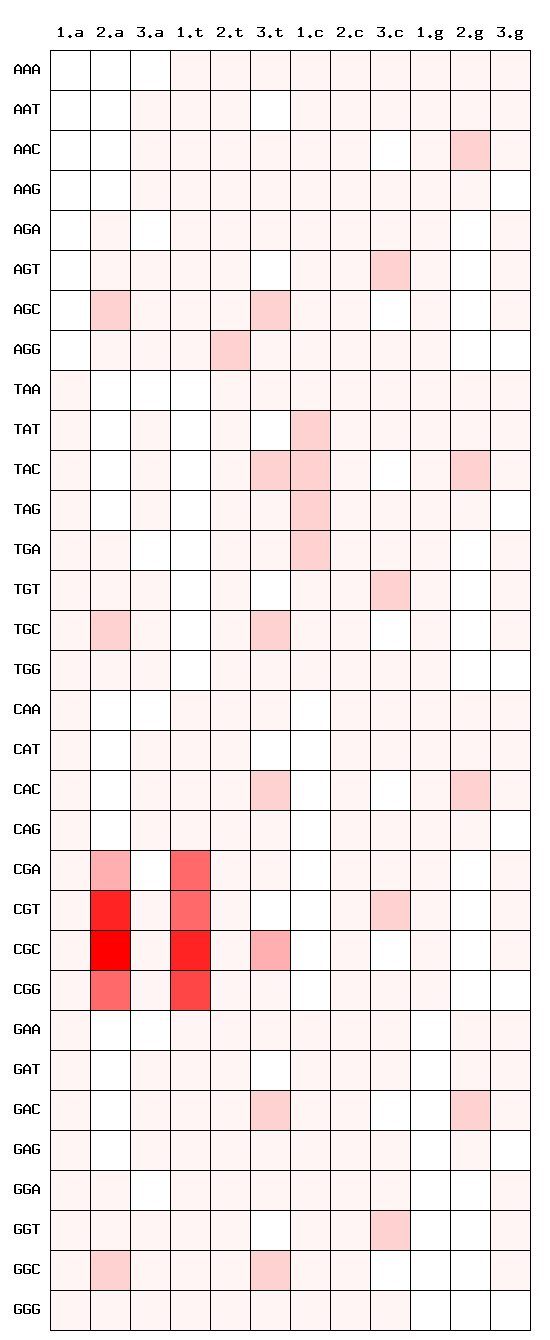 | 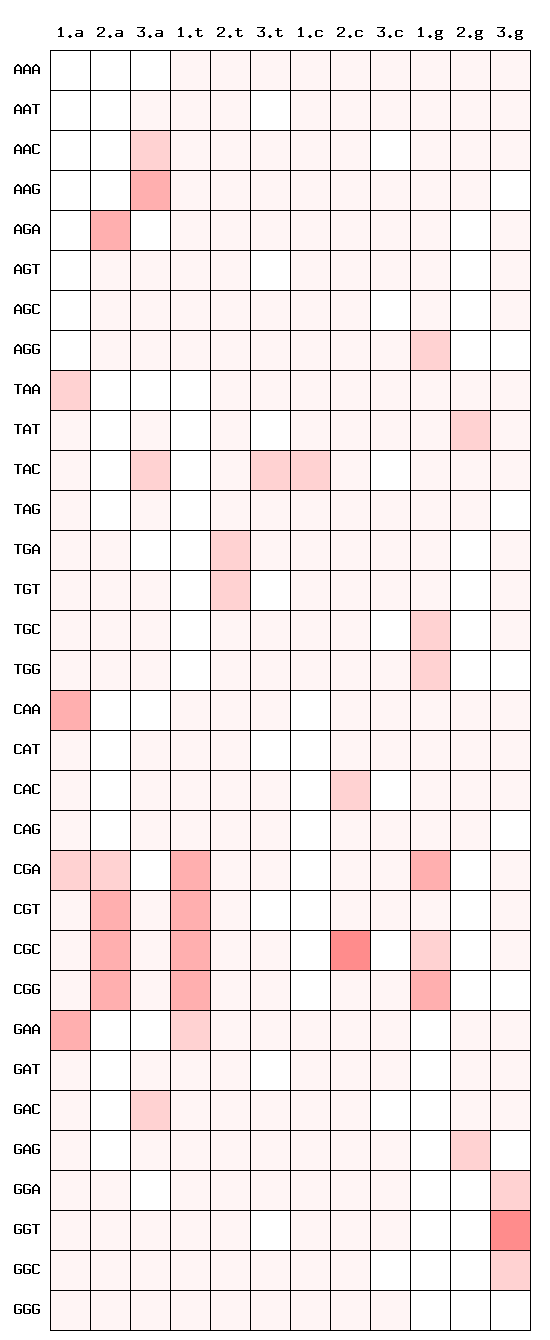 |
| \| Trinucleotide \| Mutation \| Fraction \| \| --- \| --- \| --- \| \| CGC \| 2.a \| 0.075380 \| \| CGC \| 1.t \| 0.063725 \| \| CGT \| 2.a \| 0.059675 \| \| CGG \| 1.t \| 0.047553 \| \| CGT \| 1.t \| 0.044923 \| | \| Trinucleotide \| Mutation \| Fraction \| \| --- \| --- \| --- \| \| CGC \| 2.c \| 0.033061 \| \| GGT \| 3.g \| 0.025967 \| \| CGT \| 2.a \| 0.024996 \| \| CGC \| 2.a \| 0.022474 \| \| CGG \| 1.t \| 0.021956 \| |

**Supplemental Figure 10**


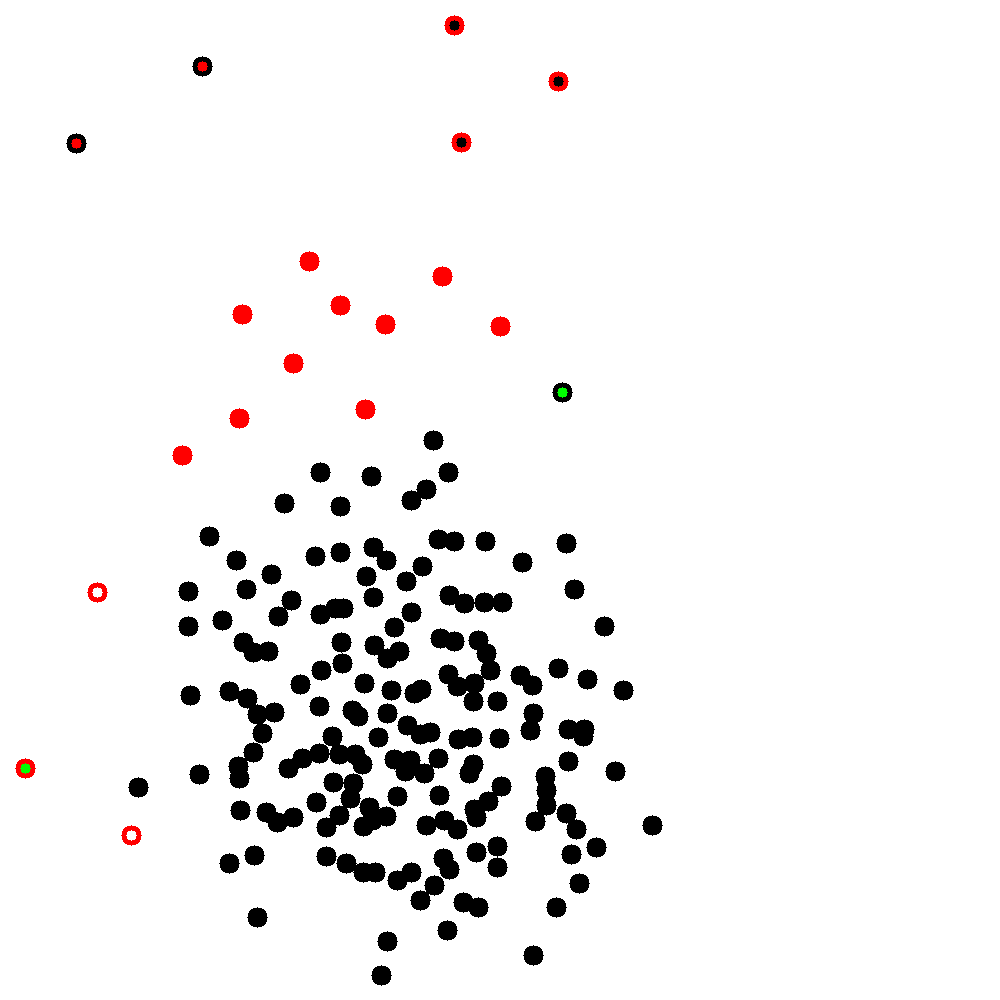

**Supplemental Figure 11**

| DO45157 | DO45301 | DO45293 |
| --- | --- | --- |
| 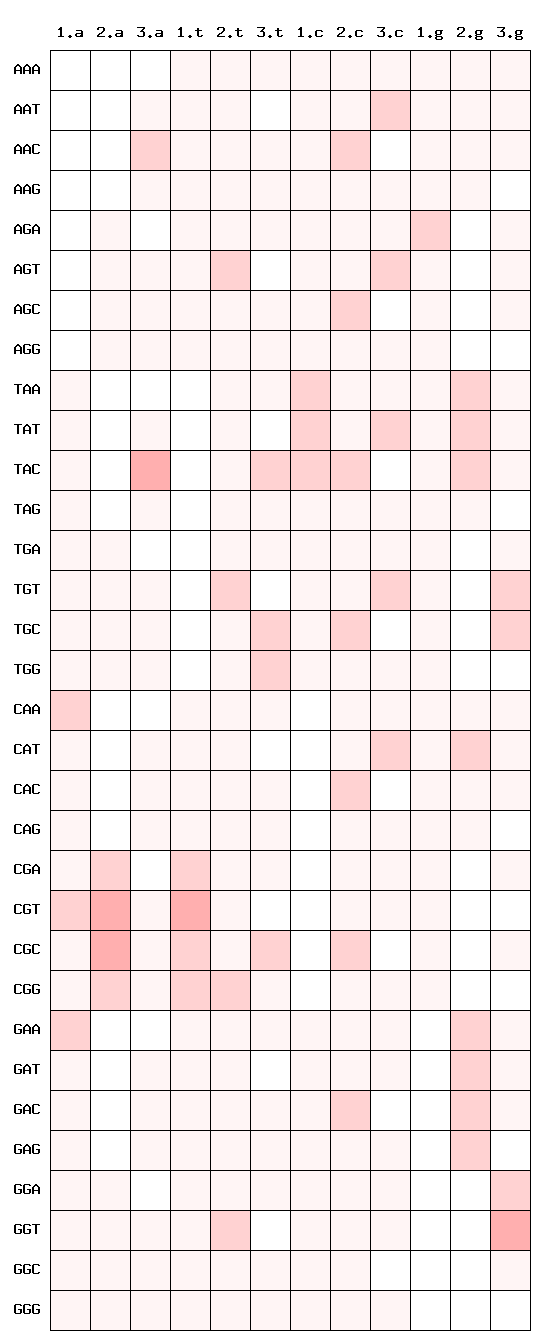 | 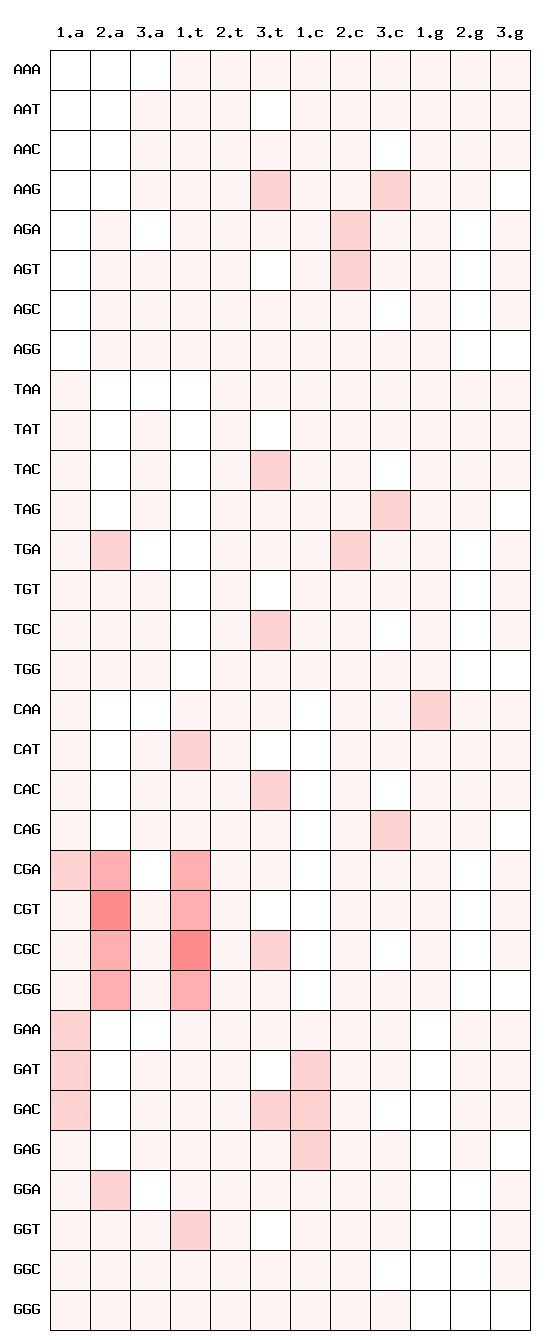 | 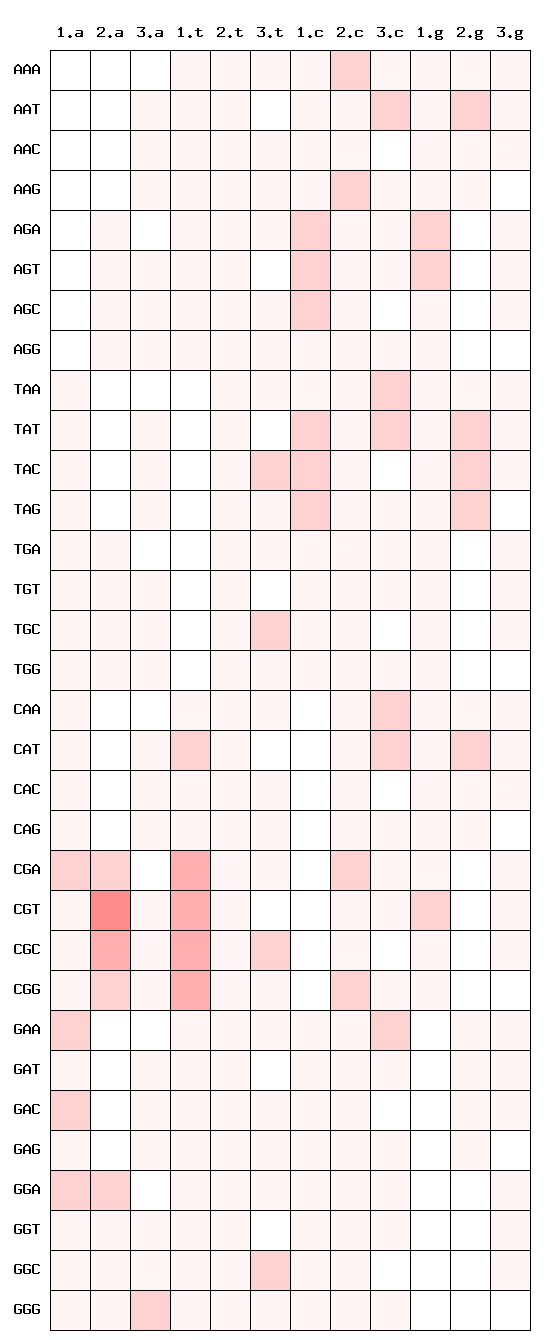 |
| \| Trinucleotide \| Mutation \| Fraction \| \| --- \| --- \| --- \| \| GGT \| 3.g \| 0.024474 \| \| CGT \| 2.a \| 0.023823 \| \| TAC \| 3.a \| 0.023262 \| \| CGT \| 1.t \| 0.015882 \| \| CGC \| 2.a \| 0.015127 \| | \| Trinucleotide \| Mutation \| Fraction \| \| --- \| --- \| --- \| \| CGT \| 2.a \| 0.033259 \| \| CGC \| 1.t \| 0.026134 \| \| CGG \| 1.t \| 0.022192 \| \| CGA \| 1.t \| 0.020752 \| \| CGC \| 2.a \| 0.020741 \| | \| Trinucleotide \| Mutation \| Fraction \| \| --- \| --- \| --- \| \| CGT \| 2.a \| 0.027093 \| \| CGC \| 2.a \| 0.021204 \| \| CGG \| 1.t \| 0.020952 \| \| CGT \| 1.t \| 0.018606 \| \| CGA \| 1.t \| 0.017481 \| |

| DO48682 | DO45211 | DO45305 |
| --- | --- | --- |
| 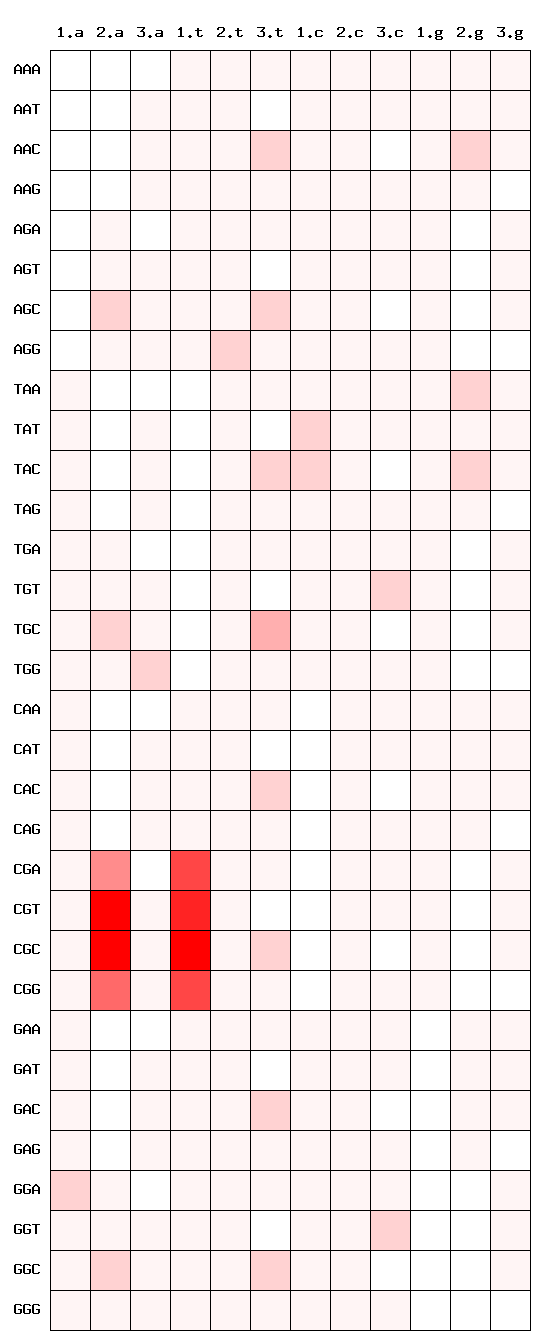 | 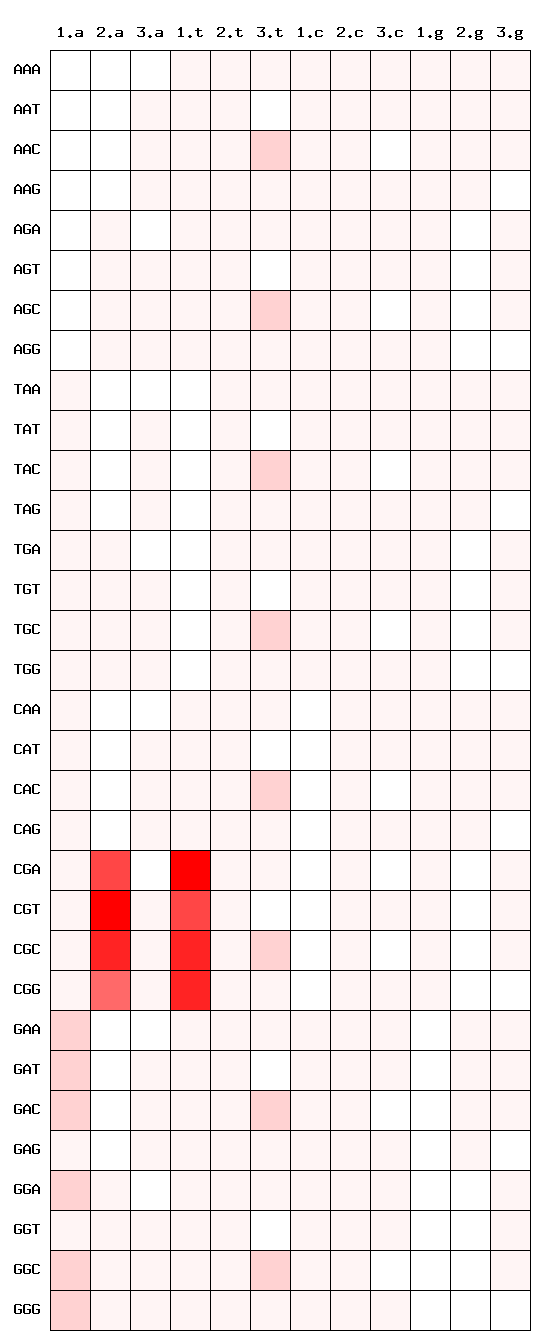 | 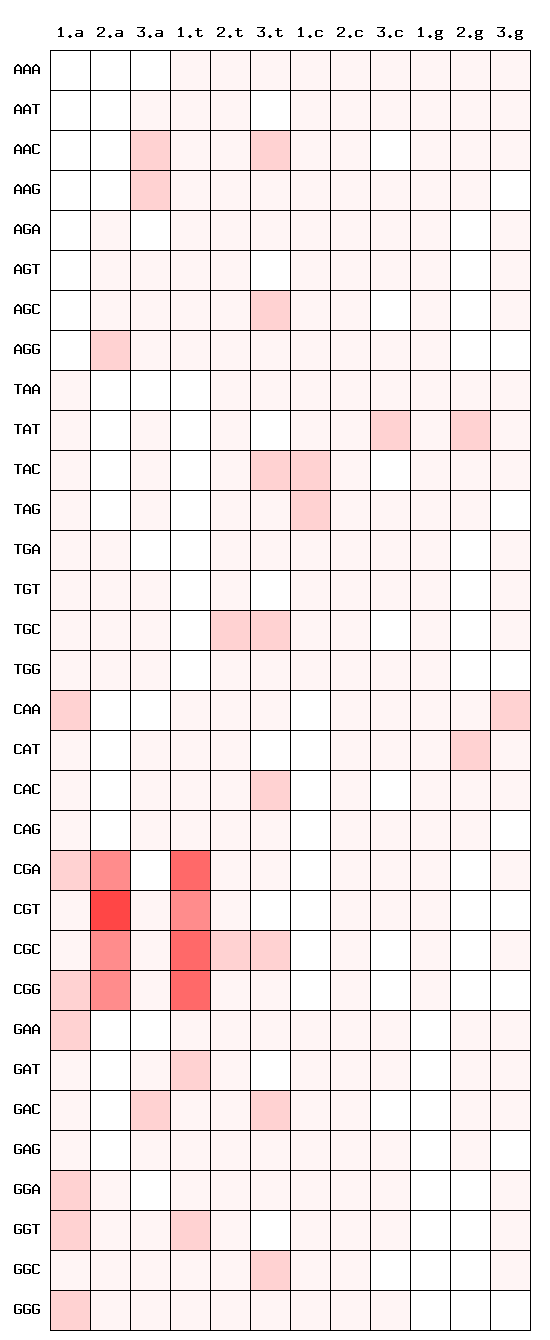 |
| \| Trinucleotide \| Mutation \| Fraction \| \| --- \| --- \| --- \| \| CGC \| 2.a \| 0.080251 \| \| CGT \| 2.a \| 0.076826 \| \| CGC \| 1.t \| 0.071140 \| \| CGT \| 1.t \| 0.056926 \| \| CGG \| 1.t \| 0.054450 \| | \| Trinucleotide \| Mutation \| Fraction \| \| --- \| --- \| --- \| \| CGT \| 2.a \| 0.078230 \| \| CGA \| 1.t \| 0.069898 \| \| CGC \| 2.a \| 0.058375 \| \| CGC \| 1.t \| 0.056901 \| \| CGG \| 1.t \| 0.056222 \| | \| Trinucleotide \| Mutation \| Fraction \| \| --- \| --- \| --- \| \| CGT \| 2.a \| 0.053035 \| \| CGC \| 1.t \| 0.042288 \| \| CGA \| 1.t \| 0.037968 \| \| CGG \| 1.t \| 0.036489 \| \| CGC \| 2.a \| 0.034420 \| |

| DO23521 |
| --- |
| 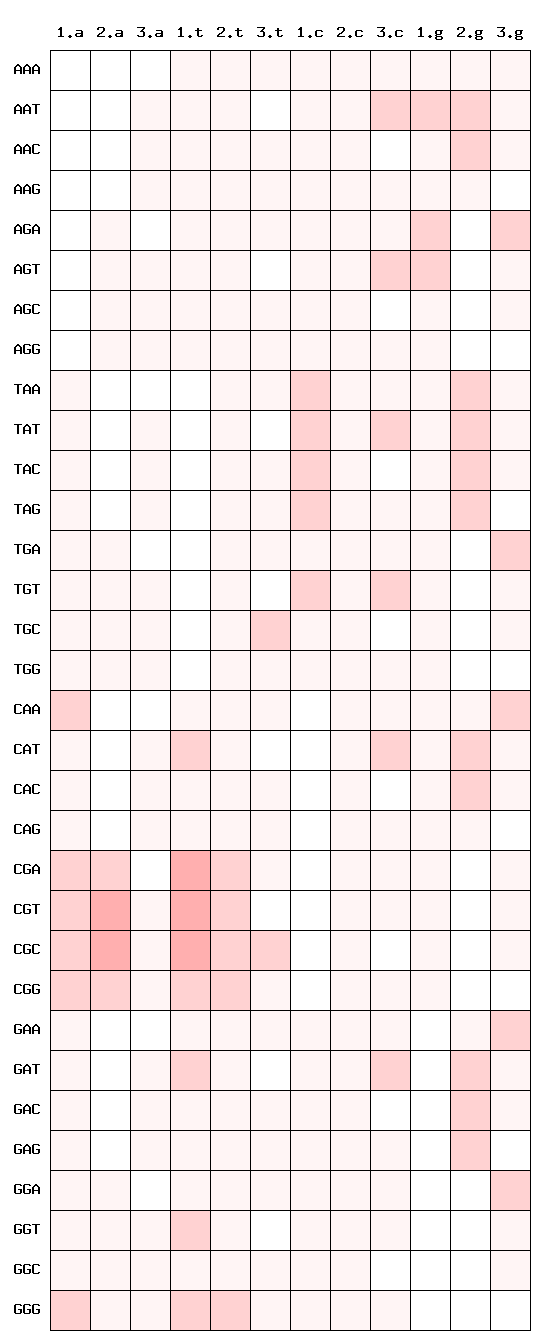 |
| \| Trinucleotide \| Mutation \| Fraction \| \| --- \| --- \| --- \| \| CGT \| 2.a \| 0.024074 \| \| CGA \| 1.t \| 0.018466 \| \| CGC \| 1.t \| 0.017474 \| \| CGC \| 2.a \| 0.016815 \| \| CGT \| 1.t \| 0.015420 \| |

**Supplemental Figure 12**


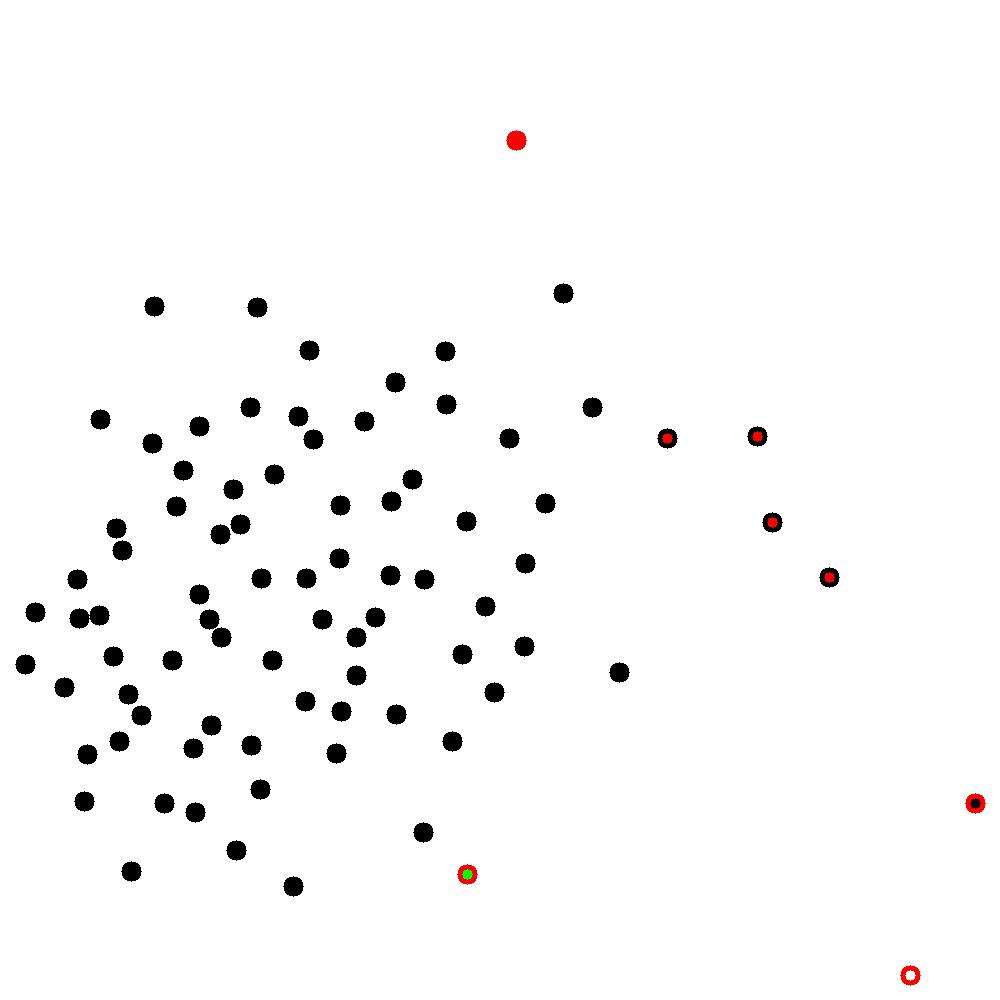


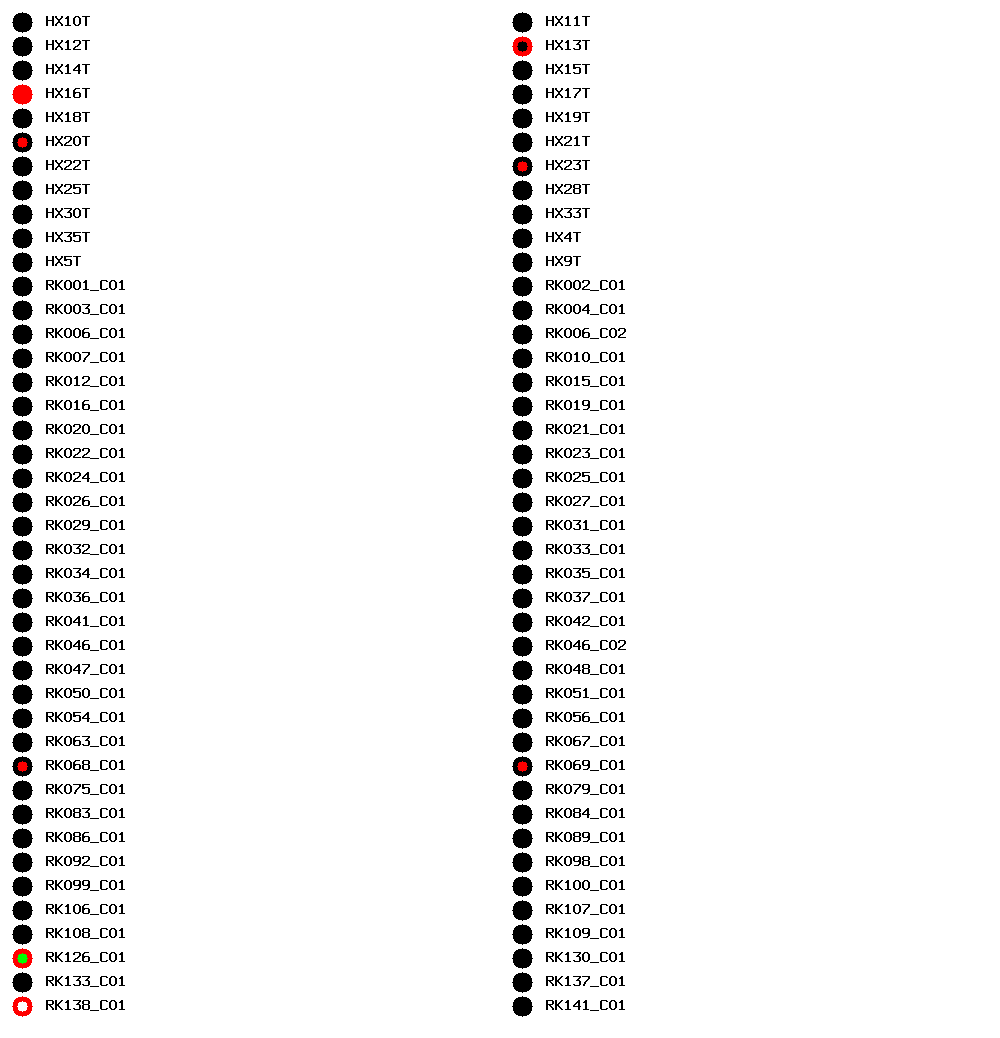


**Supplemental Figure 13**

| HX13T | HX16T | RK126_C01 |
| --- | --- | --- |
| 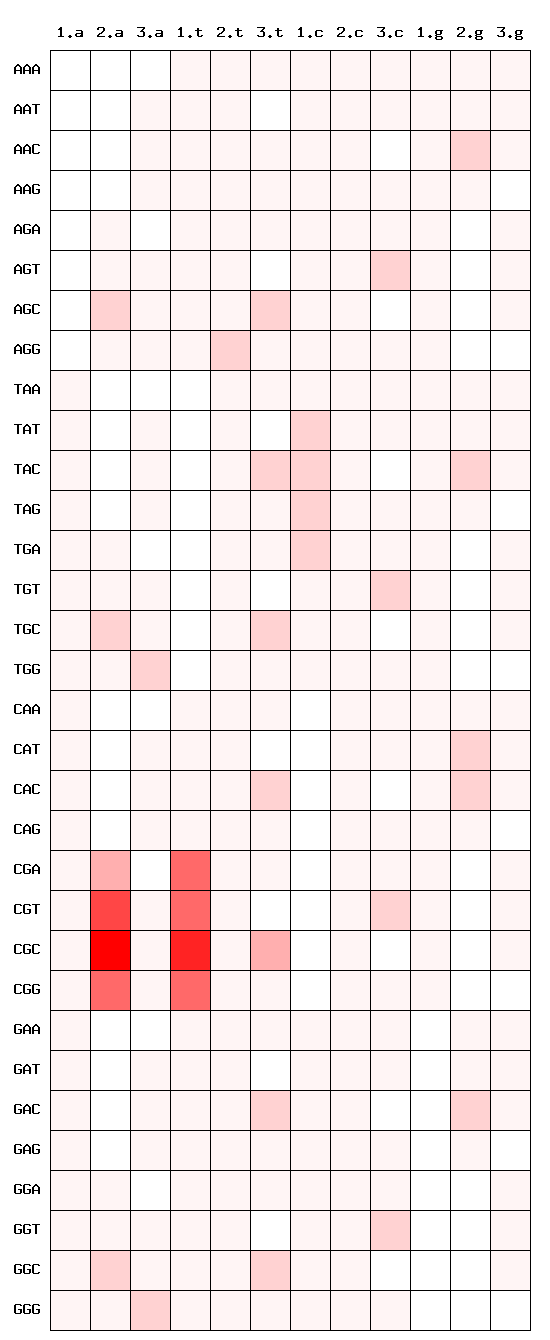 | 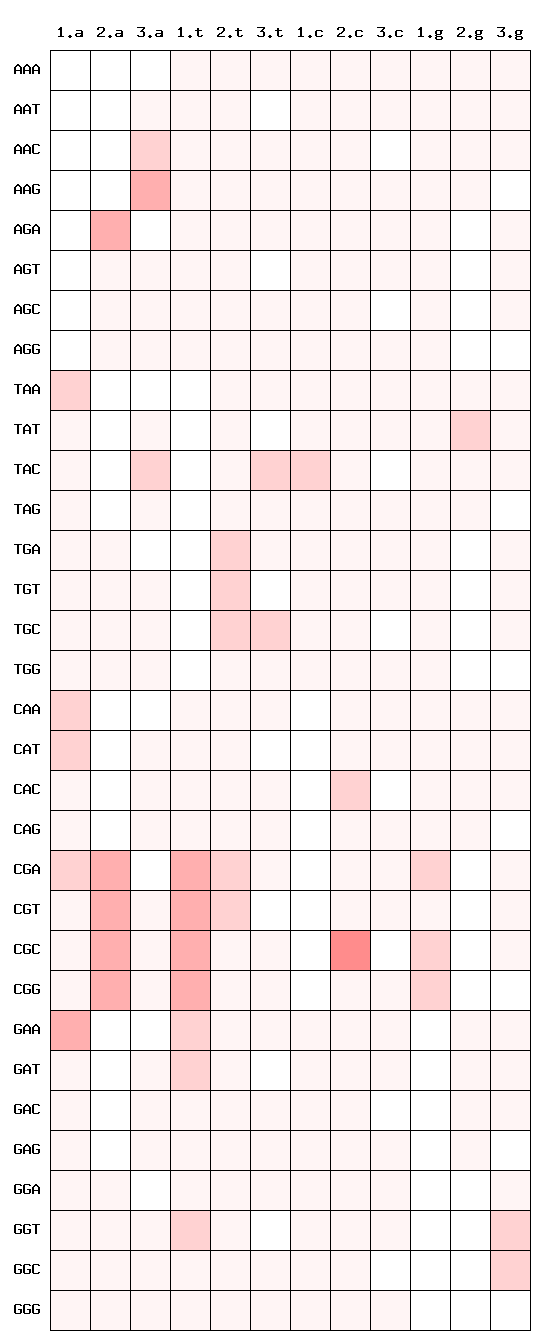 | 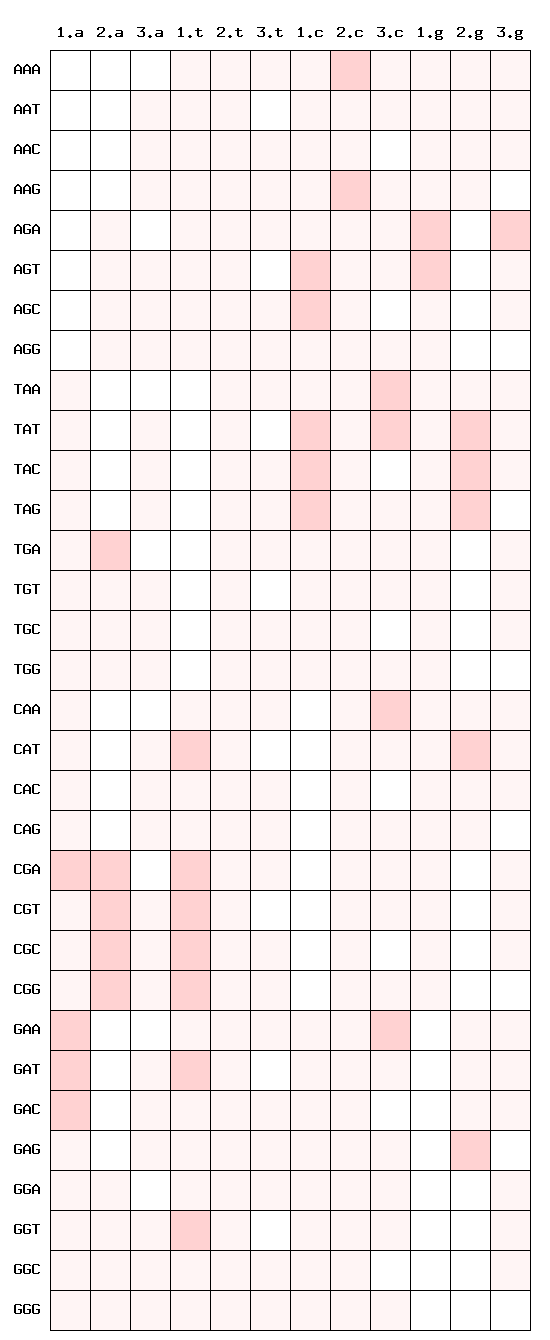 |
| \| Trinucleotide \| Mutation \| Fraction \| \| --- \| --- \| --- \| \| CGC \| 2.a \| 0.071808 \| \| CGC \| 1.t \| 0.058980 \| \| CGT \| 2.a \| 0.054585 \| \| CGG \| 1.t \| 0.044462 \| \| CGT \| 1.t \| 0.043093 \| | \| Trinucleotide \| Mutation \| Fraction \| \| --- \| --- \| --- \| \| CGC \| 2.c \| 0.026796 \| \| CGT \| 2.a \| 0.024714 \| \| CGG \| 1.t \| 0.022342 \| \| CGC \| 1.t \| 0.019871 \| \| CGC \| 2.a \| 0.019269 \| | \| Trinucleotide \| Mutation \| Fraction \| \| --- \| --- \| --- \| \| AAG \| 2.c \| 0.013492 \| \| CGT \| 2.a \| 0.013113 \| \| CGG \| 1.t \| 0.010491 \| \| CGC \| 2.a \| 0.010334 \| \| CGC \| 1.t \| 0.010334 \| |

| RK138_C01 | HX23T | RK051_C01 |
| --- | --- | --- |
| 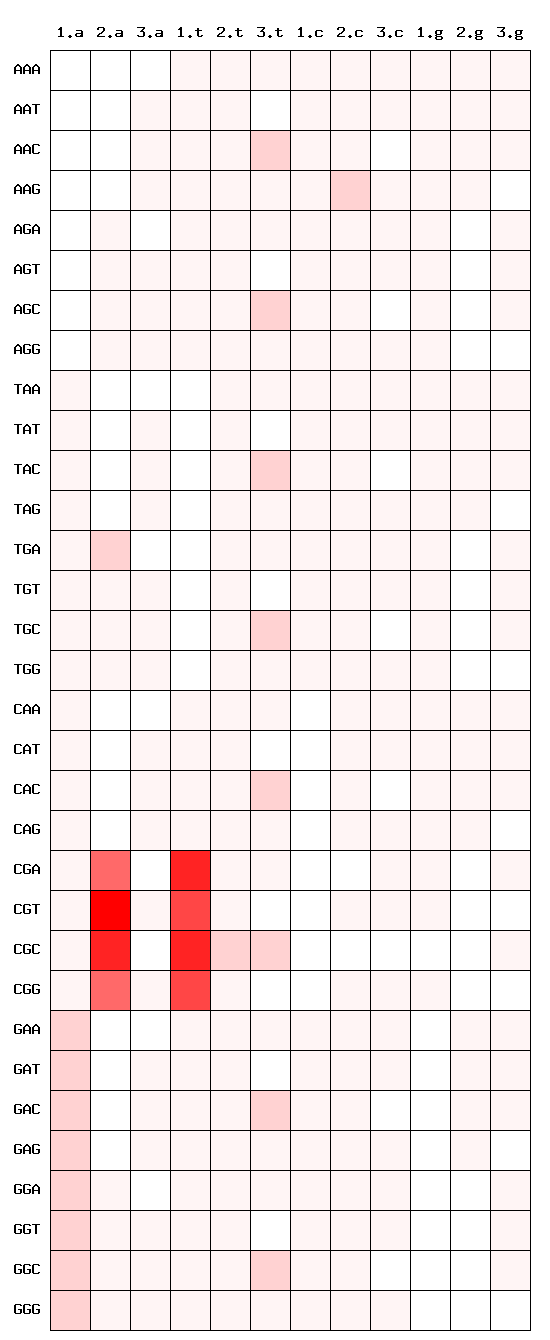 | 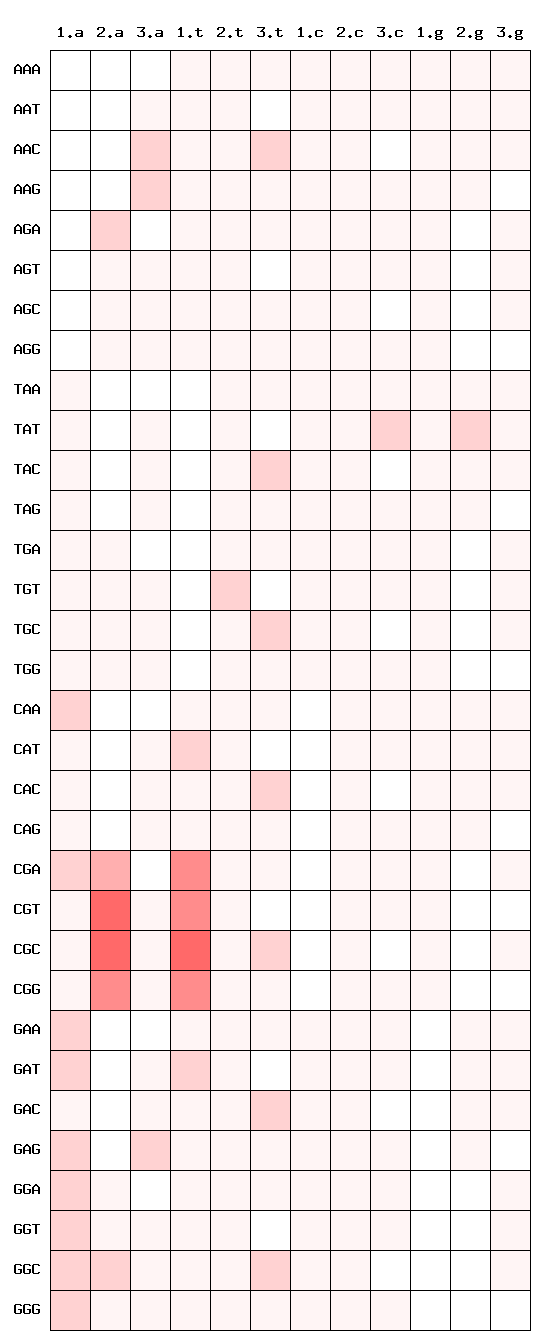 | 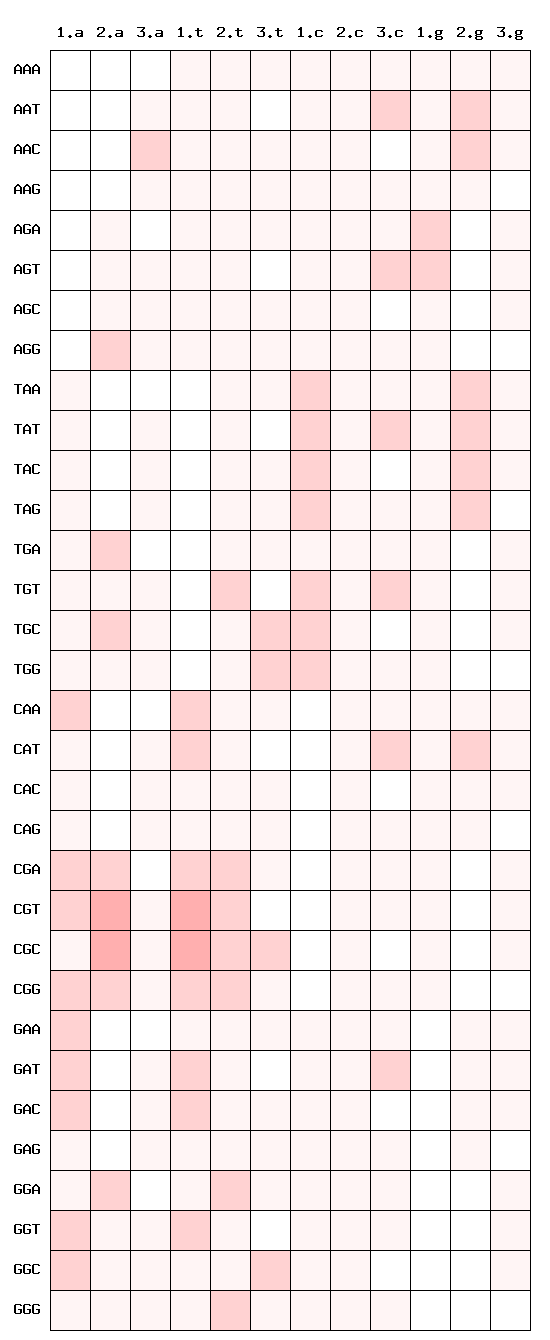 |
| \| Trinucleotide \| Mutation \| Fraction \| \| --- \| --- \| --- \| \| CGT \| 2.a \| 0.076030 \| \| CGA \| 1.t \| 0.061439 \| \| CGC \| 1.t \| 0.057059 \| \| CGC \| 2.a \| 0.056494 \| \| CGG \| 1.t \| 0.054597 \| | \| Trinucleotide \| Mutation \| Fraction \| \| --- \| --- \| --- \| \| CGT \| 2.a \| 0.044306 \| \| CGC \| 2.a \| 0.036936 \| \| CGC \| 1.t \| 0.035937 \| \| CGA \| 1.t \| 0.032027 \| \| CGG \| 1.t \| 0.030579 \| | \| Trinucleotide \| Mutation \| Fraction \| \| --- \| --- \| --- \| \| CGT \| 2.a \| 0.018826 \| \| CGC \| 1.t \| 0.017393 \| \| CGT \| 1.t \| 0.016088 \| \| CGC \| 2.a \| 0.015062 \| \| CGA \| 2.a \| 0.013456 \| |

**Supplemental Figure 14**

| LUAD-S01345 | LU-A08-43 |
| --- | --- |
| 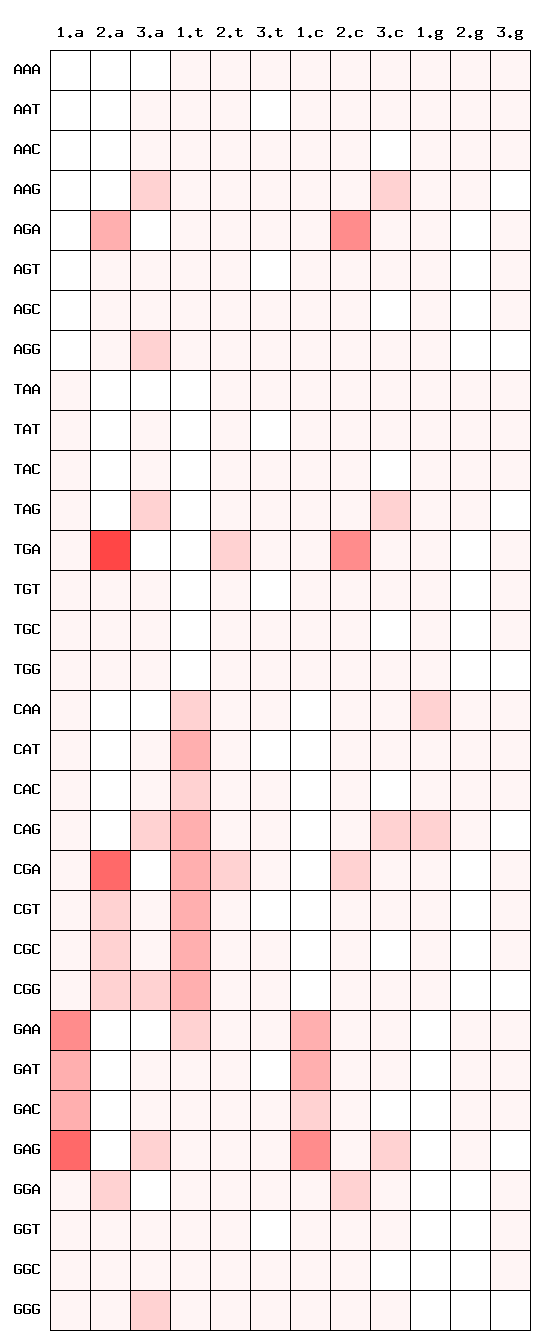 | 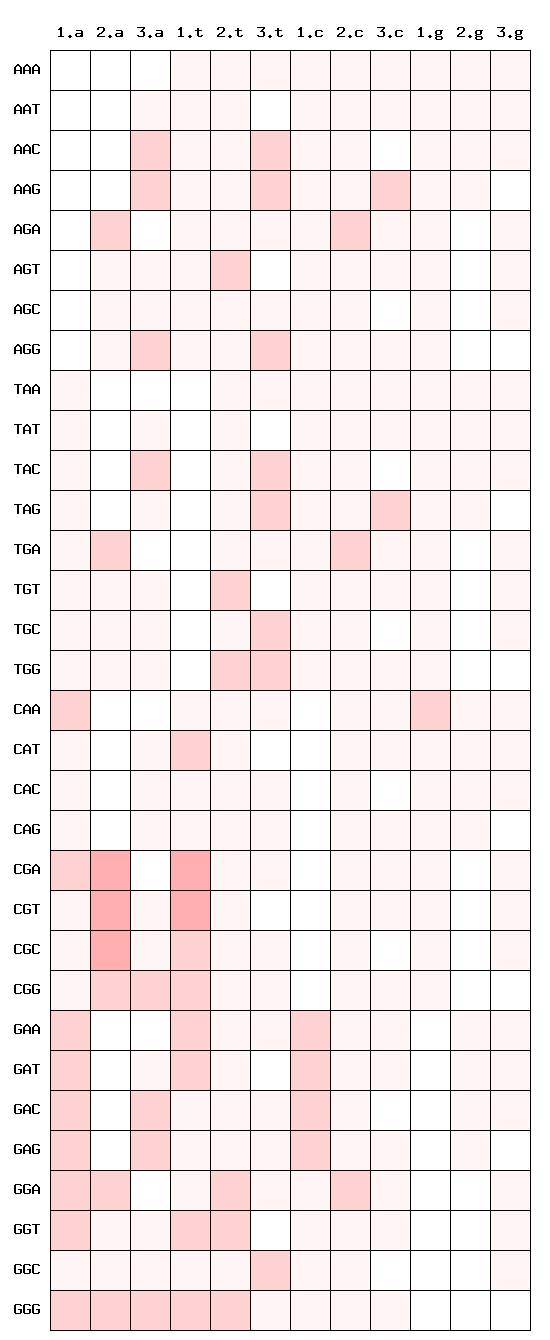 |
| \| Trinucleotide \| Mutation \| Fraction \| \| --- \| --- \| --- \| \| TGA \| 2.a \| 0.047110 \| \| CGA \| 2.a \| 0.043168 \| \| GAG \| 1.a \| 0.037350 \| \| GAA \| 1.a \| 0.033246 \| \| GAG \| 1.c \| 0.033116 \| | \| Trinucleotide \| Mutation \| Fraction \| \| --- \| --- \| --- \| \| CGA \| 1.t \| 0.019120 \| \| CGT \| 2.a \| 0.018276 \| \| CGC \| 2.a \| 0.016362 \| \| CGT \| 1.t \| 0.016282 \| \| CGA \| 2.a \| 0.015334 \| |

| LUAD-S01341 | LUAD-FH5PJ |
| --- | --- |
| 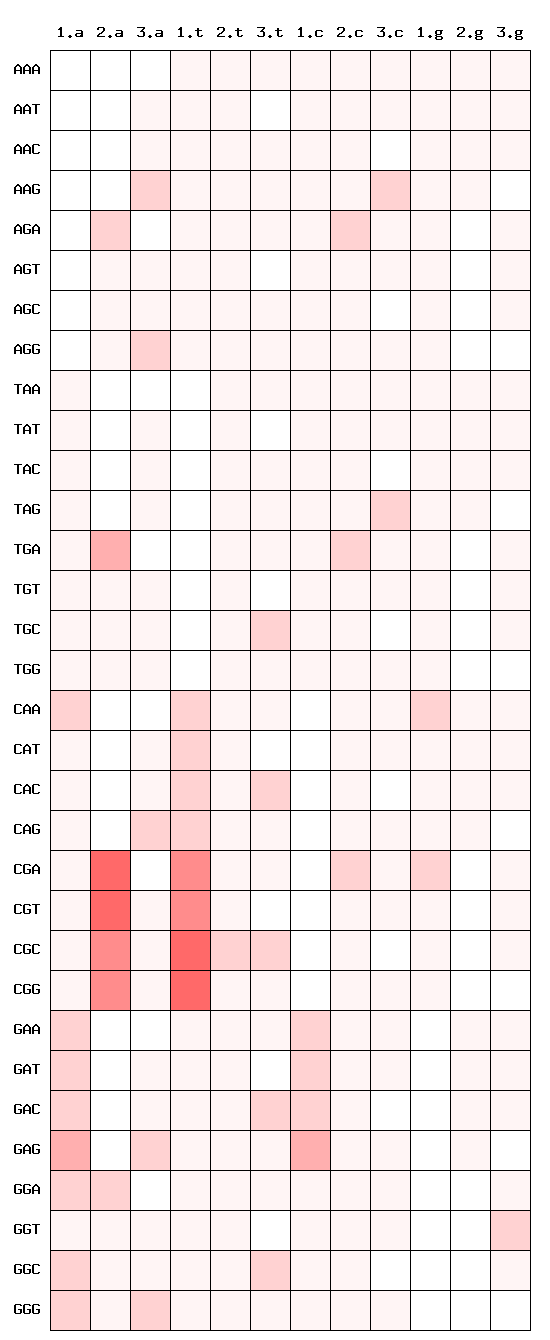 | 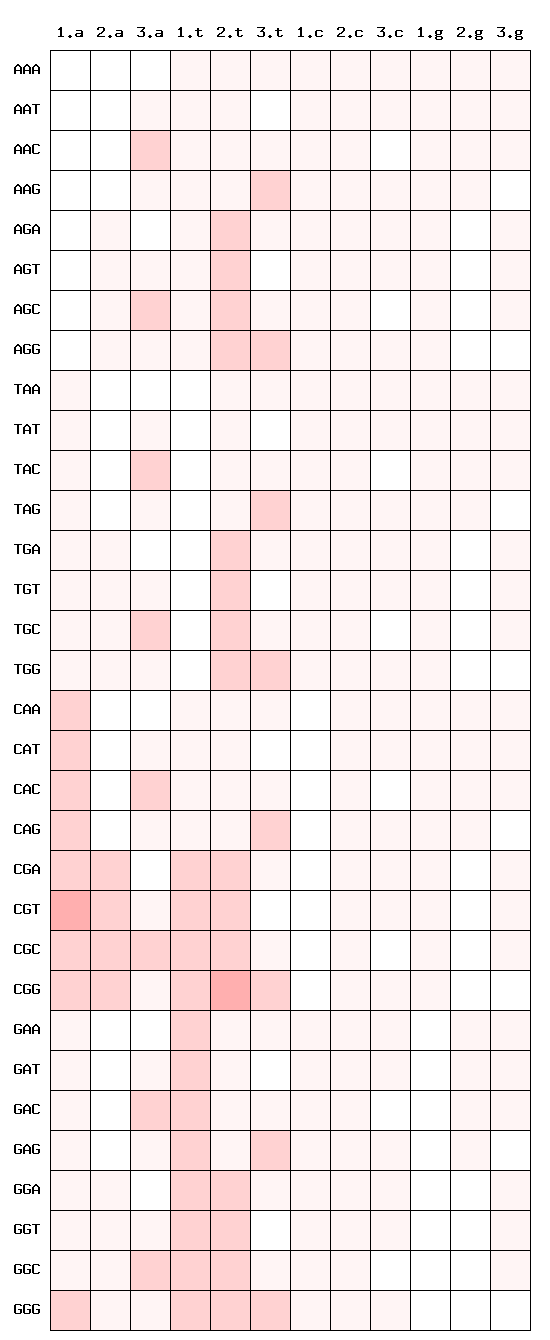 |
| \| Trinucleotide \| Mutation \| Fraction \| \| --- \| --- \| --- \| \| CGG \| 1.t \| 0.038775 \| \| CGA \| 2.a \| 0.036969 \| \| CGT \| 2.a \| 0.036113 \| \| CGC \| 1.t \| 0.035740 \| \| CGC \| 2.a \| 0.033295 \| | \| Trinucleotide \| Mutation \| Fraction \| \| --- \| --- \| --- \| \| CGG \| 2.t \| 0.016423 \| \| CGT \| 1.a \| 0.016142 \| \| CGC \| 2.t \| 0.014891 \| \| GGG \| 1.t \| 0.014751 \| \| CGT \| 2.t \| 0.014307 \| |

**Supplemental Figure 15**

| DO27771 | DO27777 | DO27779 |
| --- | --- | --- |
| 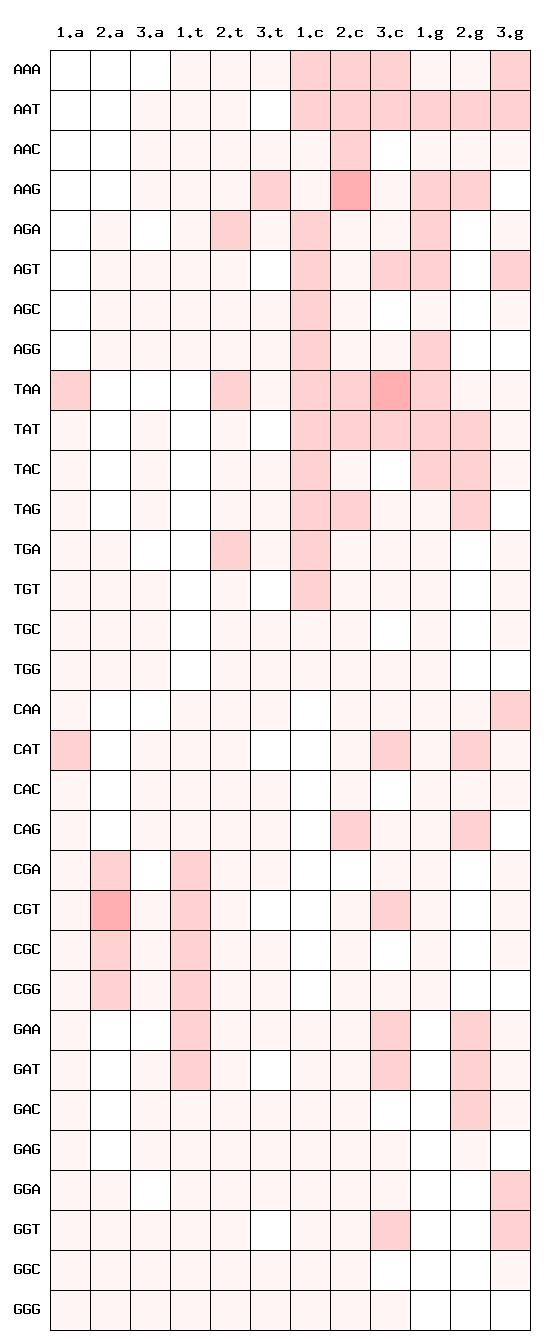 | 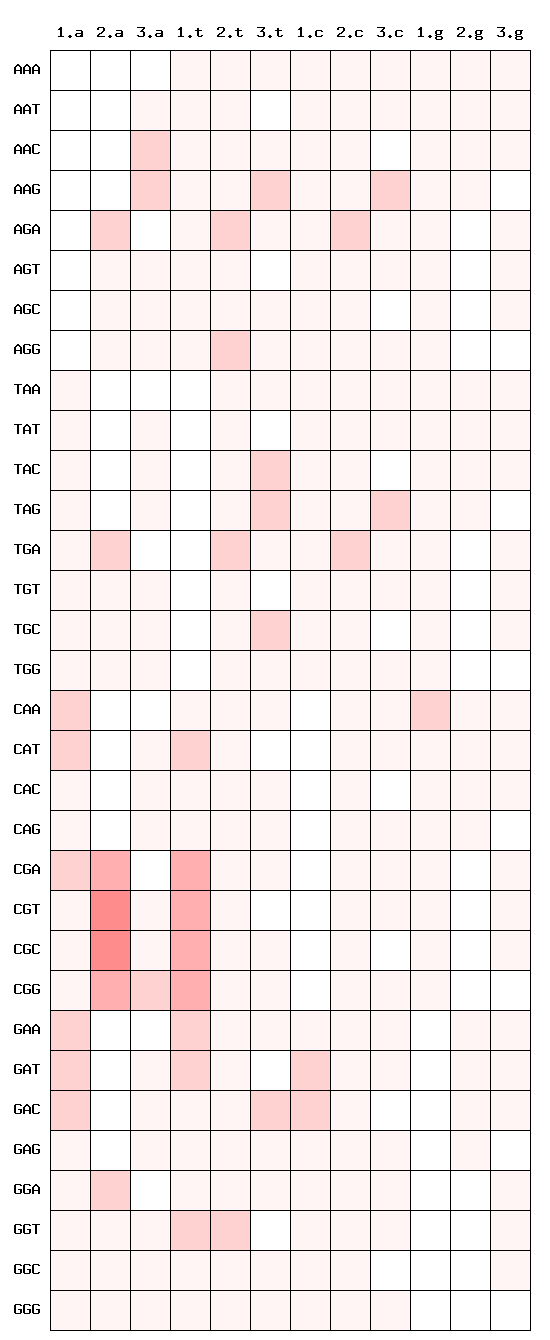 | 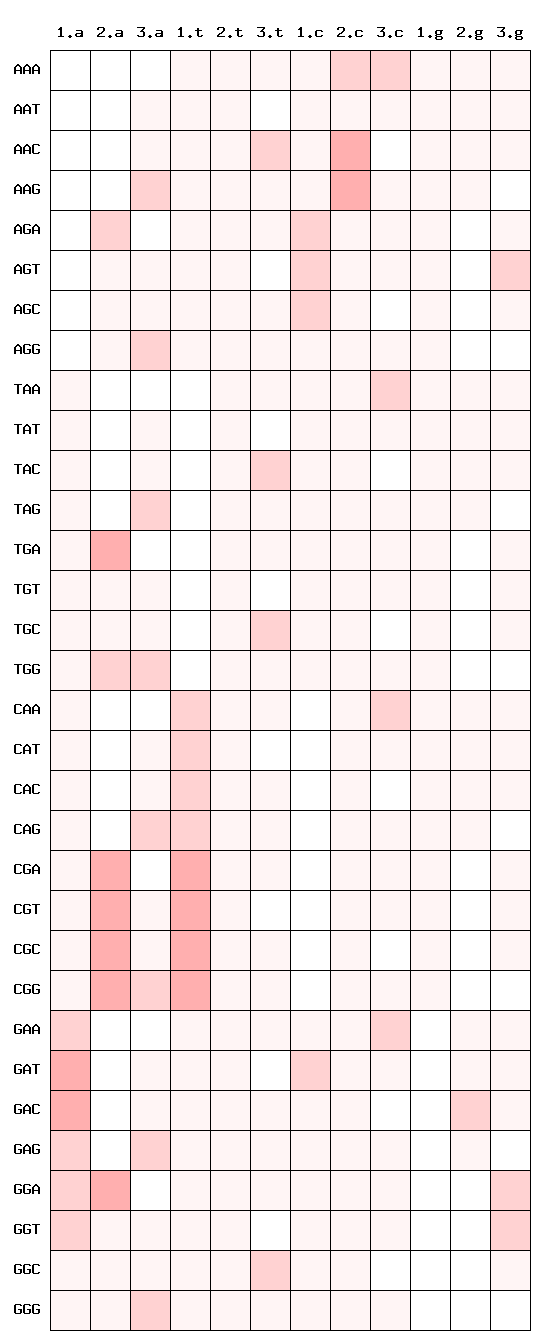 |
| \| Trinucleotide \| Mutation \| Fraction \| \| --- \| --- \| --- \| \| TAA \| 3.c \| 0.018418 \| \| AAG \| 2.c \| 0.018238 \| \| CGT \| 2.a \| 0.018199 \| \| AGT \| 1.c \| 0.014809 \| \| CGC \| 2.a \| 0.014527 \| | \| Trinucleotide \| Mutation \| Fraction \| \| --- \| --- \| --- \| \| CGT \| 2.a \| 0.027806 \| \| CGC \| 2.a \| 0.026929 \| \| CGT \| 1.t \| 0.024067 \| \| CGG \| 1.t \| 0.023870 \| \| CGA \| 1.t \| 0.022364 \| | \| Trinucleotide \| Mutation \| Fraction \| \| --- \| --- \| --- \| \| GAT \| 1.a \| 0.023642 \| \| CGT \| 2.a \| 0.022388 \| \| CGC \| 1.t \| 0.021614 \| \| CGC \| 2.a \| 0.021126 \| \| CGA \| 1.t \| 0.021032 \| |

| DO27813 | DO27805 |
| --- | --- |
| 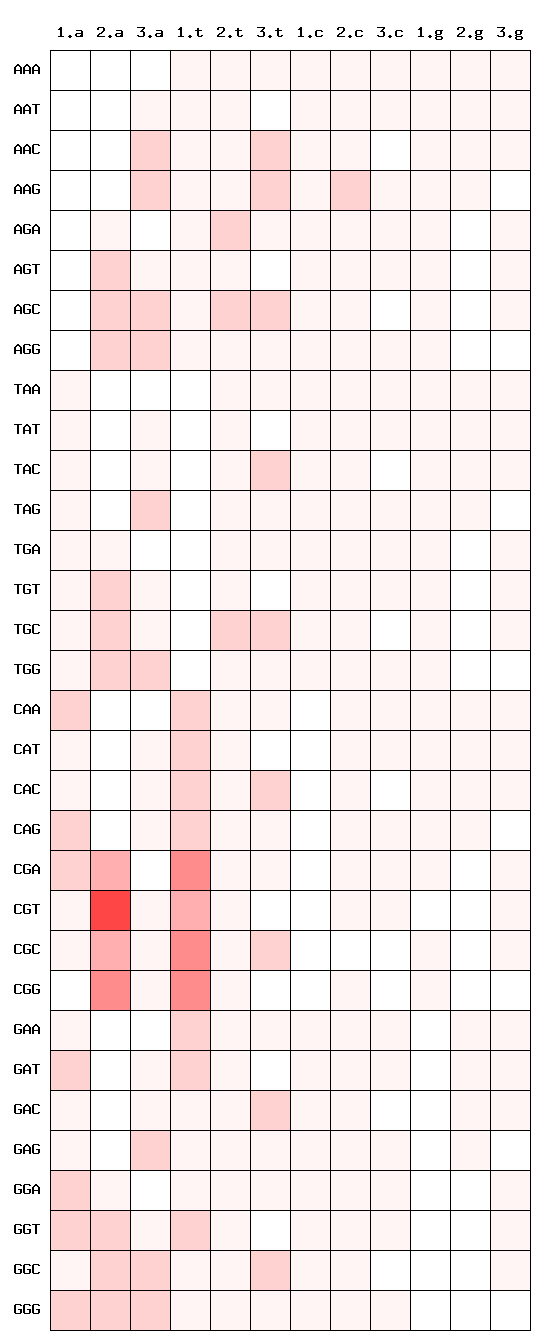 | 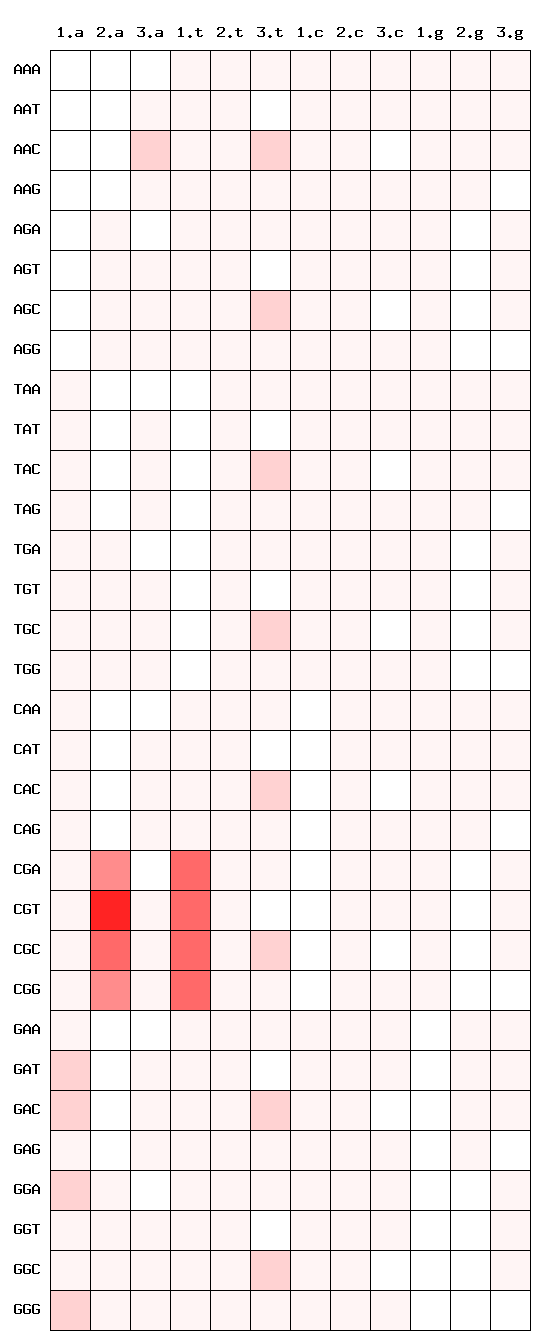 |
| \| Trinucleotide \| Mutation \| Fraction \| \| --- \| --- \| --- \| \| CGT \| 2.a \| 0.045096 \| \| CGC \| 1.t \| 0.030955 \| \| CGA \| 1.t \| 0.030121 \| \| CGG \| 1.t \| 0.026711 \| \| CGG \| 2.a \| 0.025305 \| | \| Trinucleotide \| Mutation \| Fraction \| \| --- \| --- \| --- \| \| CGT \| 2.a \| 0.061811 \| \| CGG \| 1.t \| 0.044754 \| \| CGC \| 2.a \| 0.042489 \| \| CGC \| 1.t \| 0.042196 \| \| CGA \| 1.t \| 0.038879 \| |

**Supplemental Figure 16**

| ME015 | ME009 |
| --- | --- |
| 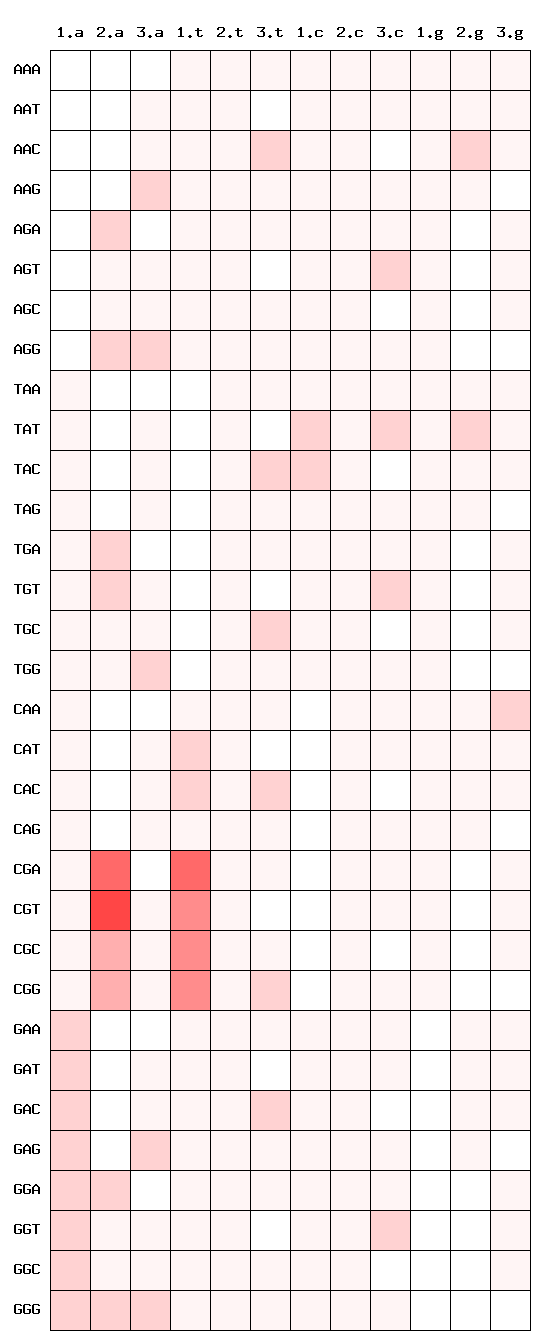 | 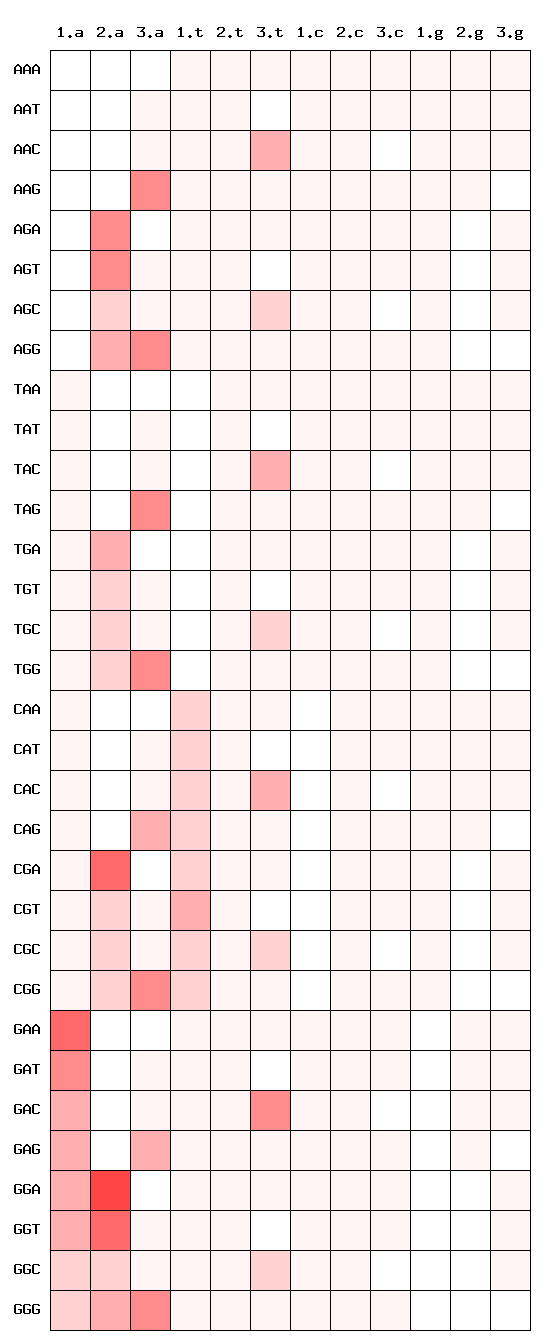 |
| \| Trinucleotide \| Mutation \| Fraction \| \| --- \| --- \| --- \| \| CGT \| 2.a \| 0.046930 \| \| CGA \| 2.a \| 0.042421 \| \| CGA \| 1.t \| 0.035648 \| \| CGT \| 1.t \| 0.034415 \| \| CGG \| 1.t \| 0.033375 \| | \| Trinucleotide \| Mutation \| Fraction \| \| --- \| --- \| --- \| \| GGA \| 2.a \| 0.047600 \| \| GAA \| 1.a \| 0.041191 \| \| GGT \| 2.a \| 0.040388 \| \| CGA \| 2.a \| 0.035755 \| \| GAT \| 1.a \| 0.034215 \| |

| ME021 | ME020 |
| --- | --- |
| 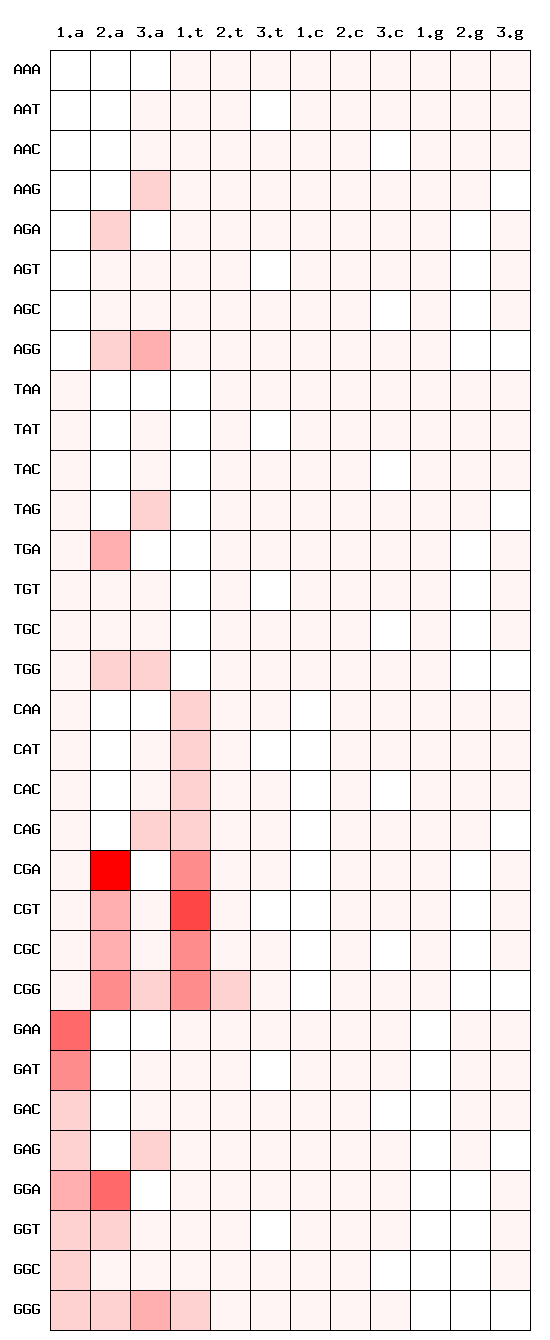 | 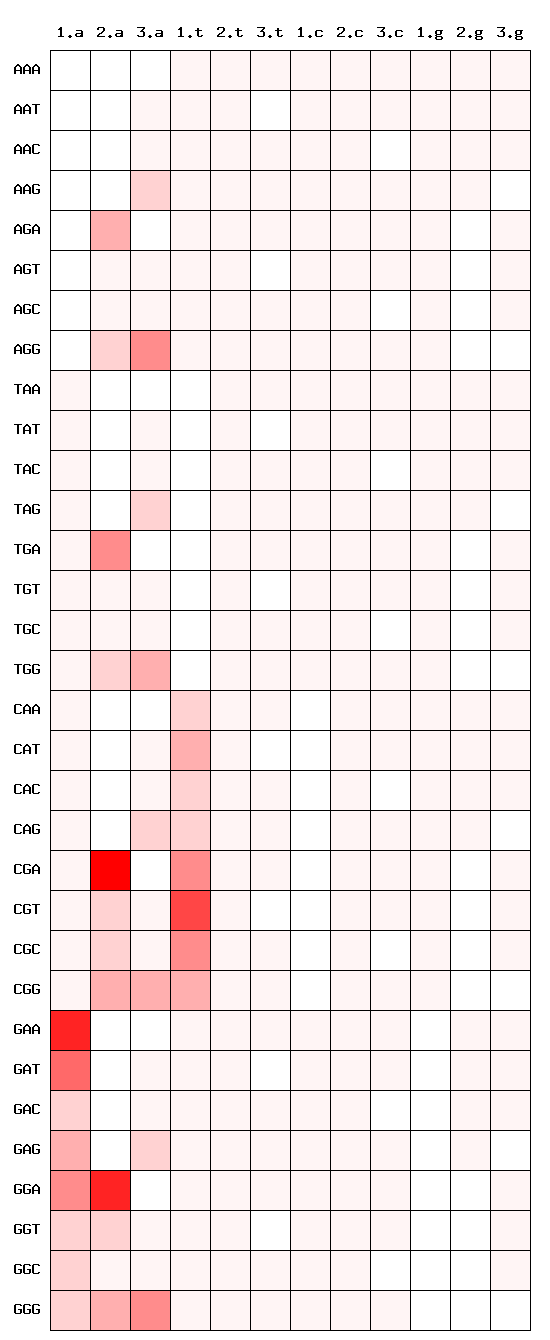 |
| \| Trinucleotide \| Mutation \| Fraction \| \| --- \| --- \| --- \| \| CGA \| 2.a \| 0.077938 \| \| CGT \| 1.t \| 0.045344 \| \| GAA \| 1.a \| 0.038812 \| \| GGA \| 2.a \| 0.037323 \| \| CGC \| 1.t \| 0.032644 \| | \| Trinucleotide \| Mutation \| Fraction \| \| --- \| --- \| --- \| \| CGA \| 2.a \| 0.108296 \| \| GGA \| 2.a \| 0.062014 \| \| GAA \| 1.a \| 0.059305 \| \| CGT \| 1.t \| 0.052573 \| \| GAT \| 1.a \| 0.040191 \| |

**Supplemental Figure 17**


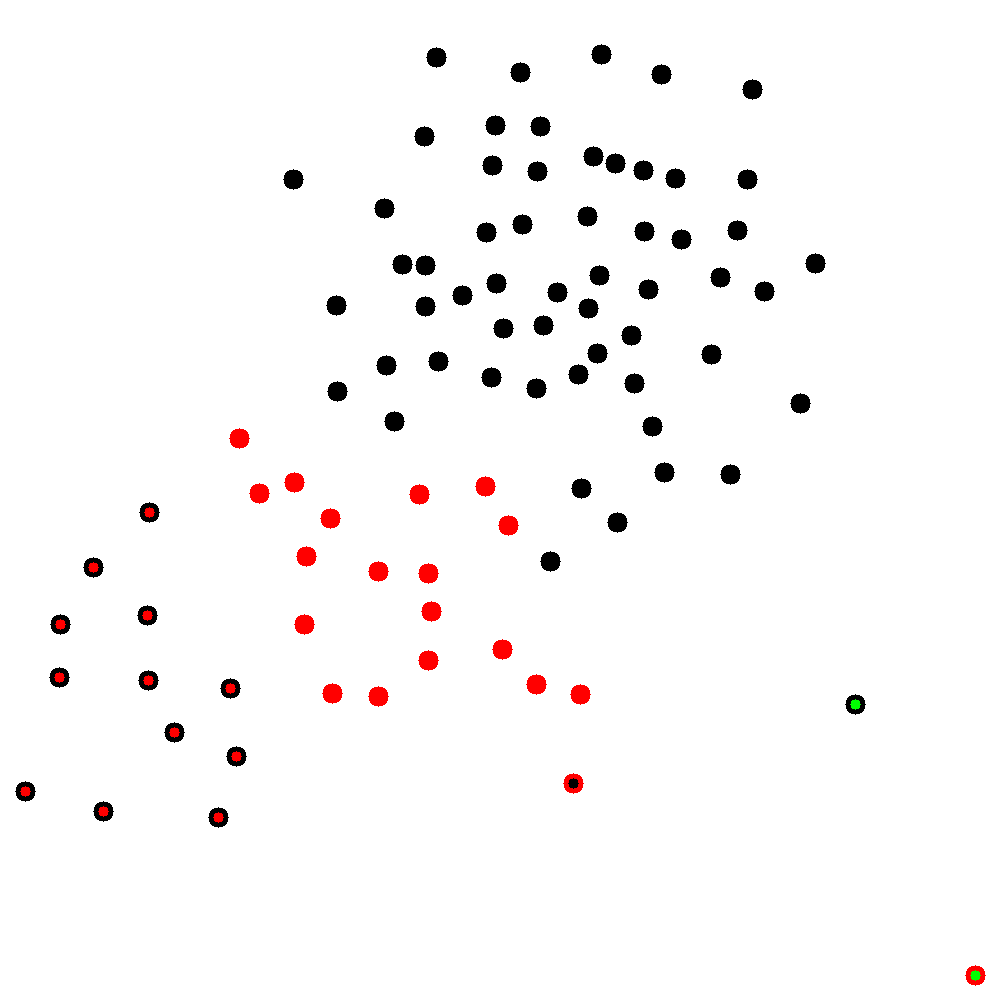


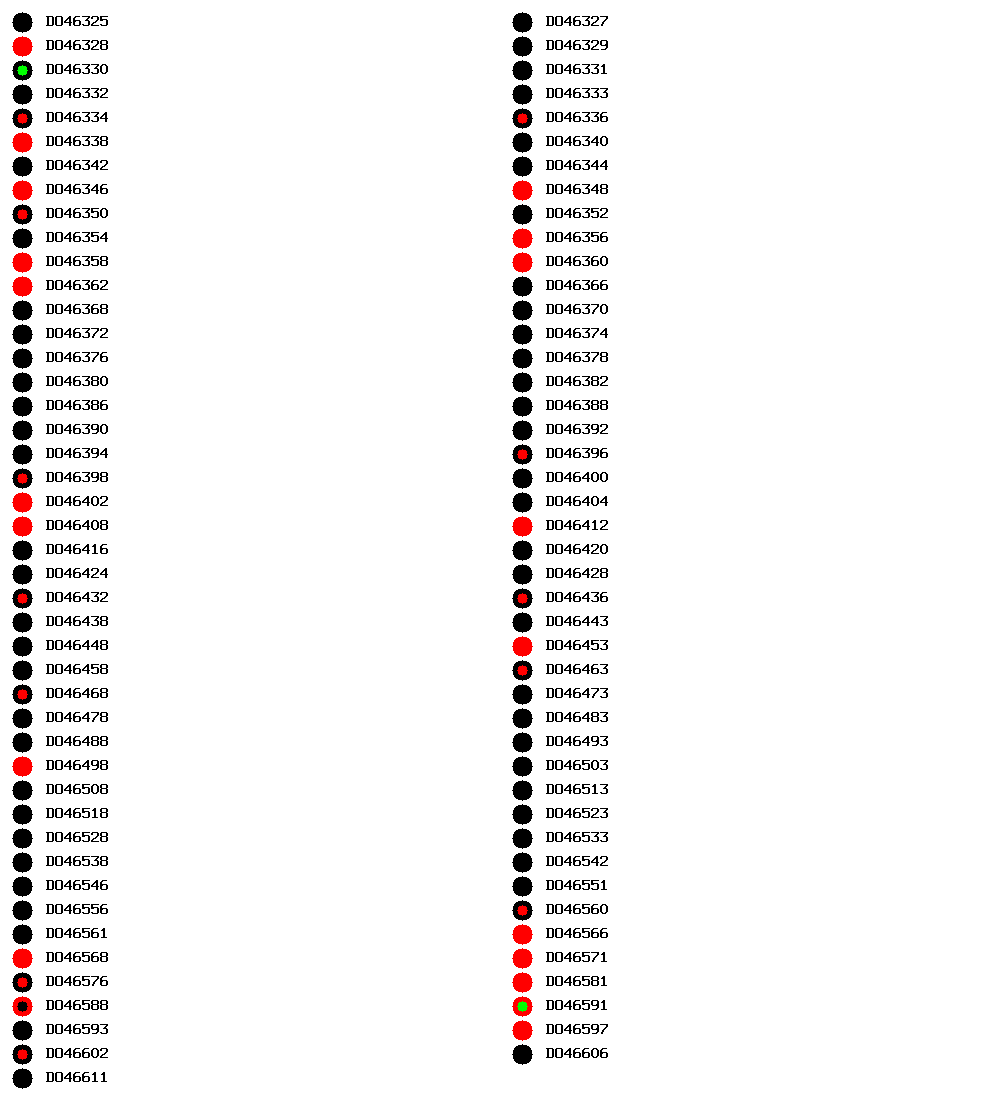


**Supplemental Figure 18**

| DO46330 | DO46588 | DO46591 |
| --- | --- | --- |
| 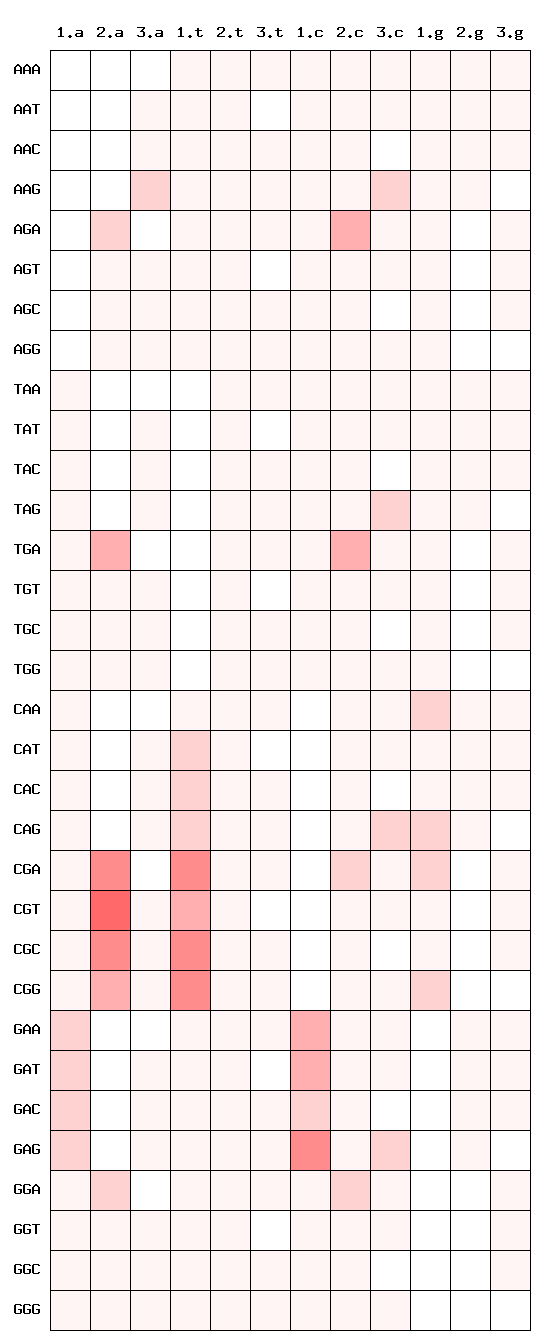 | 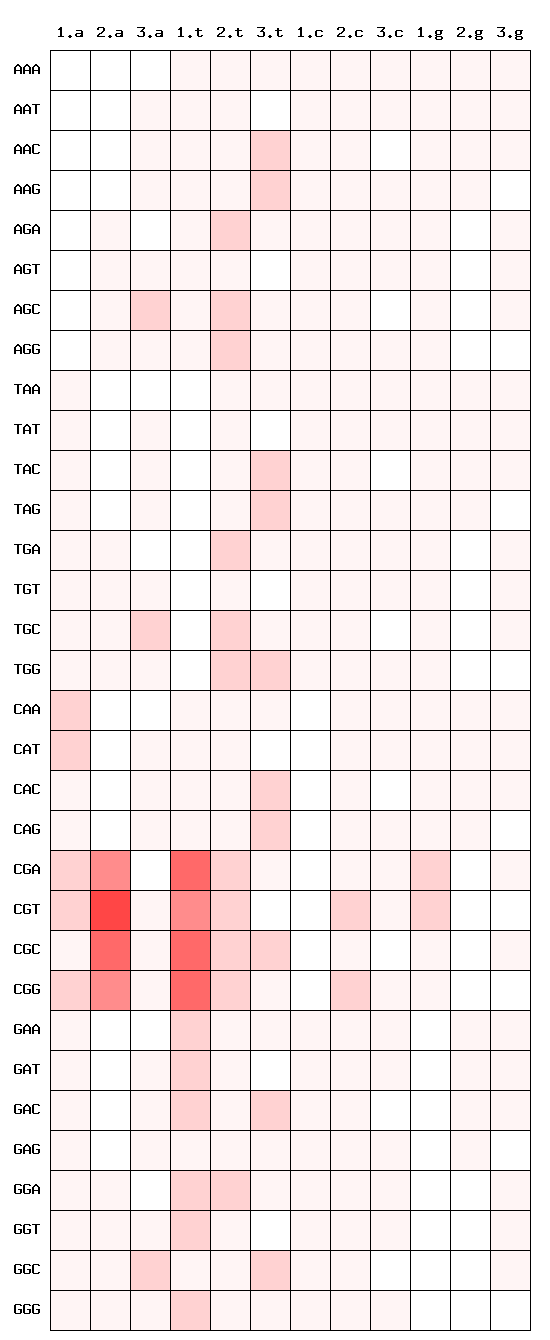 | 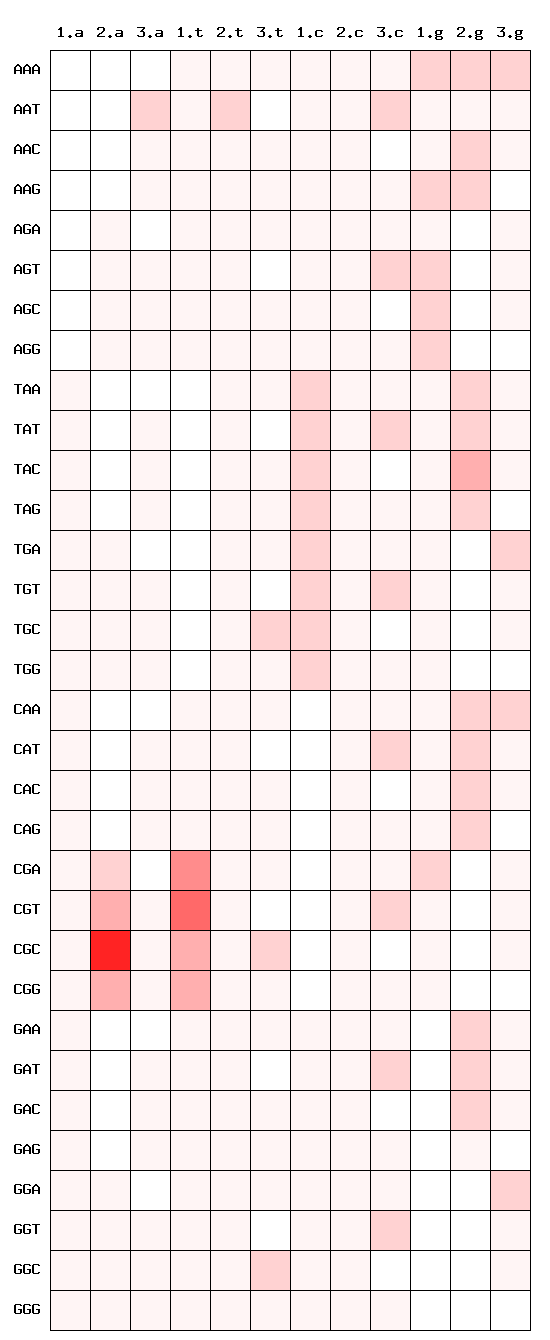 |
| \| Trinucleotide \| Mutation \| Fraction \| \| --- \| --- \| --- \| \| CGT \| 2.a \| 0.036124 \| \| CGG \| 1.t \| 0.032334 \| \| CGA \| 2.a \| 0.029070 \| \| CGA \| 1.t \| 0.026760 \| \| CGC \| 1.t \| 0.026480 \| | \| Trinucleotide \| Mutation \| Fraction \| \| --- \| --- \| --- \| \| CGT \| 2.a \| 0.045120 \| \| CGC \| 2.a \| 0.041019 \| \| CGG \| 1.t \| 0.037337 \| \| CGA \| 1.t \| 0.036450 \| \| CGC \| 1.t \| 0.035266 \| | \| Trinucleotide \| Mutation \| Fraction \| \| --- \| --- \| --- \| \| CGC \| 2.a \| 0.060153 \| \| CGT \| 1.t \| 0.038309 \| \| CGA \| 1.t \| 0.027863 \| \| CGC \| 1.t \| 0.024958 \| \| CGT \| 2.a \| 0.024162 \| |

| DO46396 | DO46356 | DO46473 |
| --- | --- | --- |
| 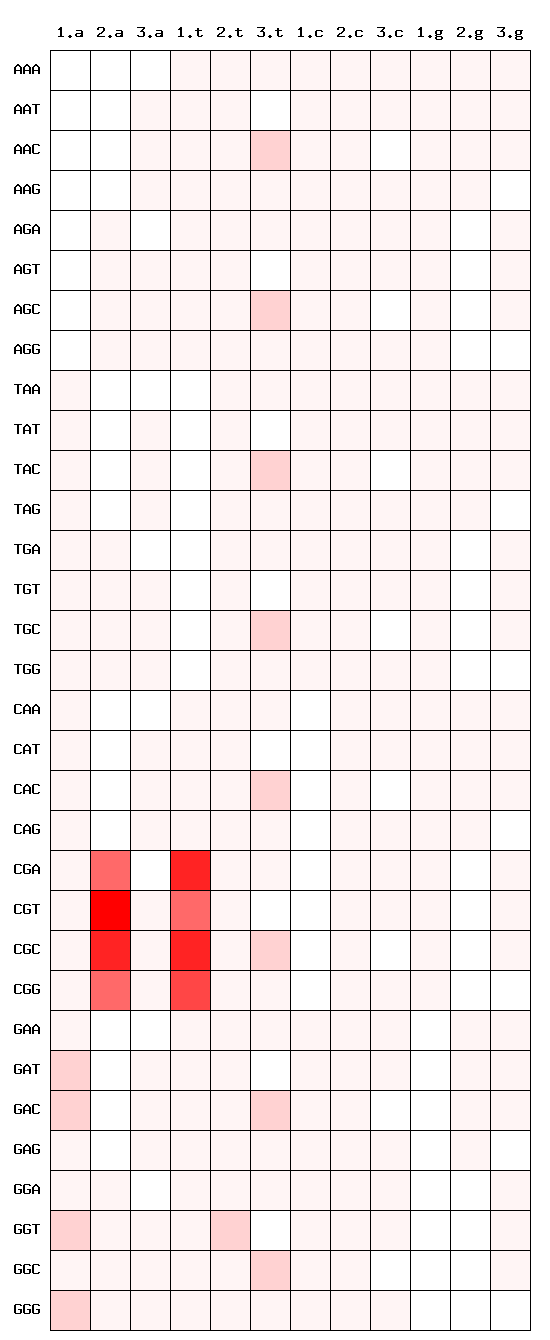 | 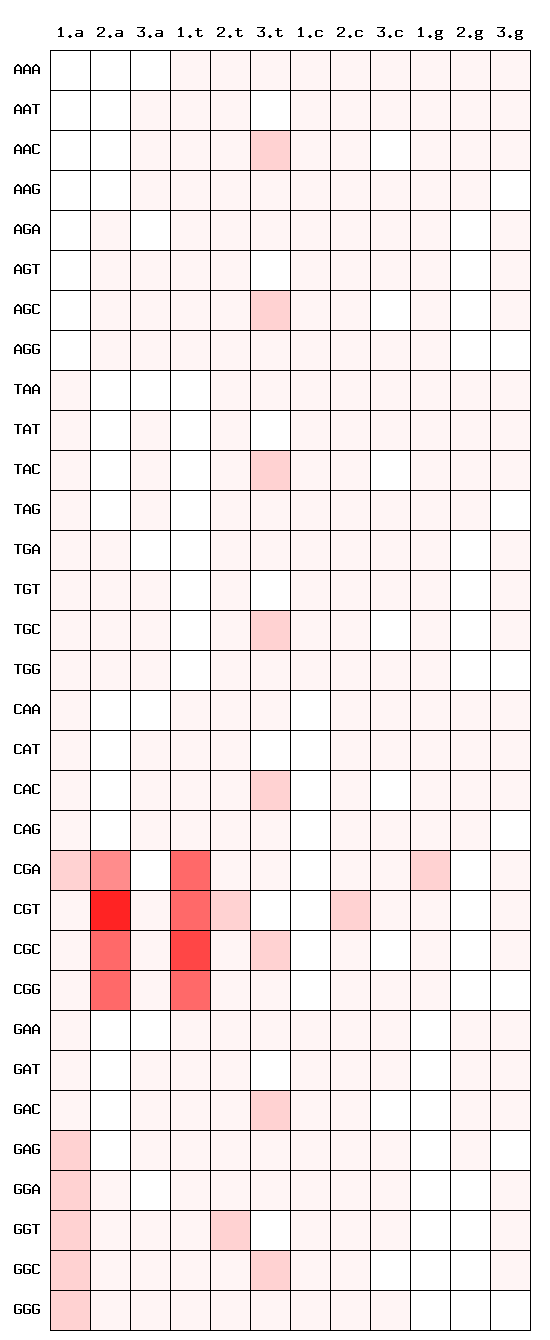 | 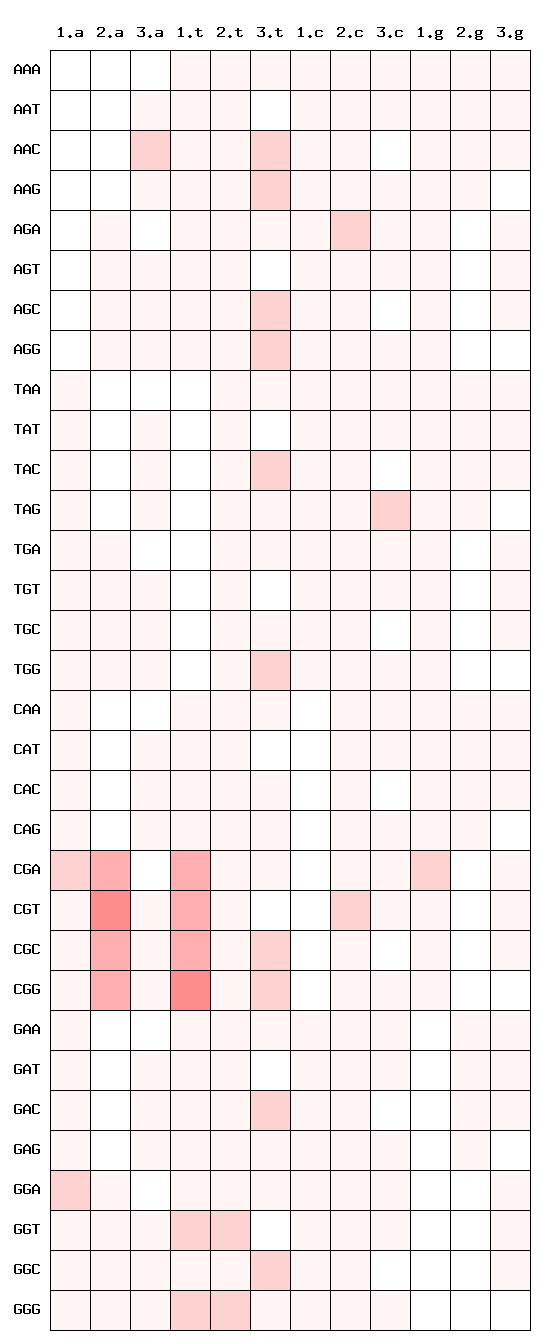 |
| \| Trinucleotide \| Mutation \| Fraction \| \| --- \| --- \| --- \| \| CGT \| 2.a \| 0.073276 \| \| CGC \| 1.t \| 0.060178 \| \| CGC \| 2.a \| 0.059683 \| \| CGA \| 1.t \| 0.055481 \| \| CGG \| 1.t \| 0.054705 \| | \| Trinucleotide \| Mutation \| Fraction \| \| --- \| --- \| --- \| \| CGT \| 2.a \| 0.057518 \| \| CGC \| 1.t \| 0.045251 \| \| CGG \| 1.t \| 0.041903 \| \| CGA \| 1.t \| 0.041530 \| \| CGC \| 2.a \| 0.039291 \| | \| Trinucleotide \| Mutation \| Fraction \| \| --- \| --- \| --- \| \| CGT \| 2.a \| 0.034336 \| \| CGG \| 1.t \| 0.025985 \| \| CGC \| 2.a \| 0.024416 \| \| CGA \| 1.t \| 0.024253 \| \| CGT \| 1.t \| 0.021907 \| |

**Supplemental Figure 19**


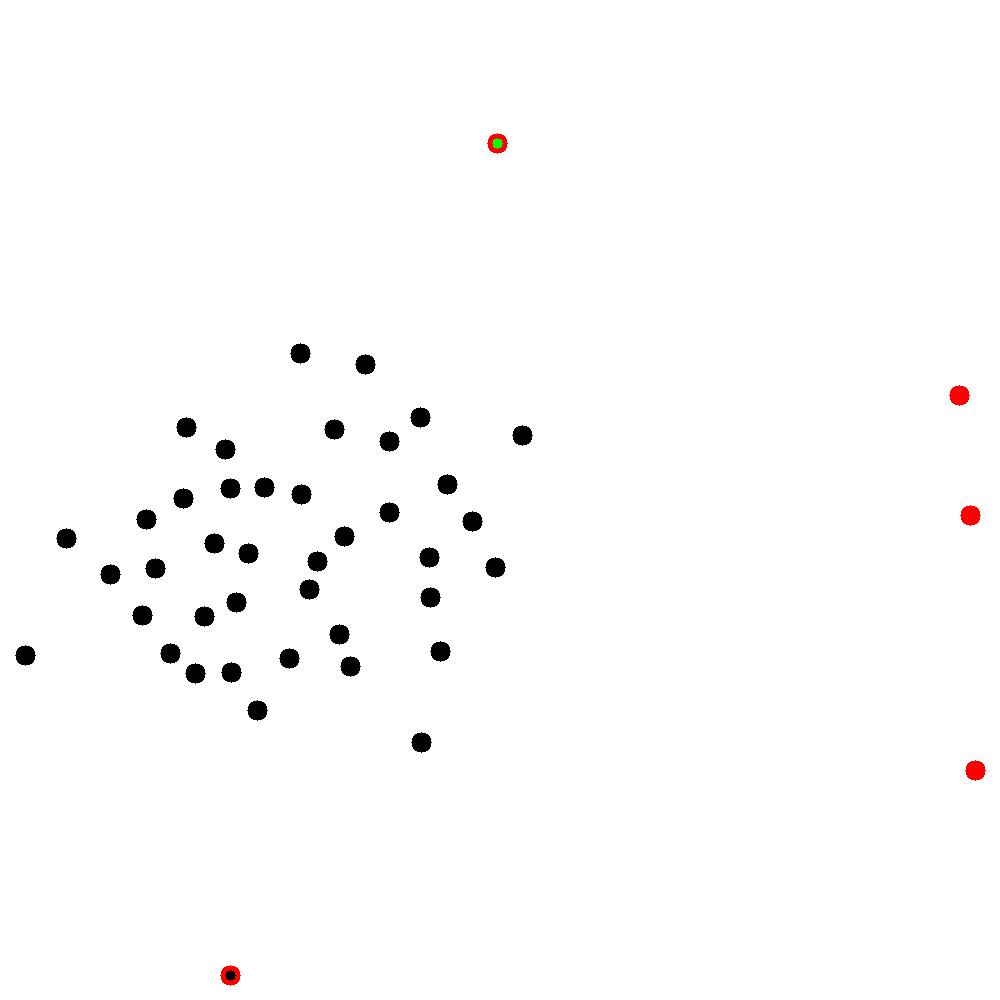


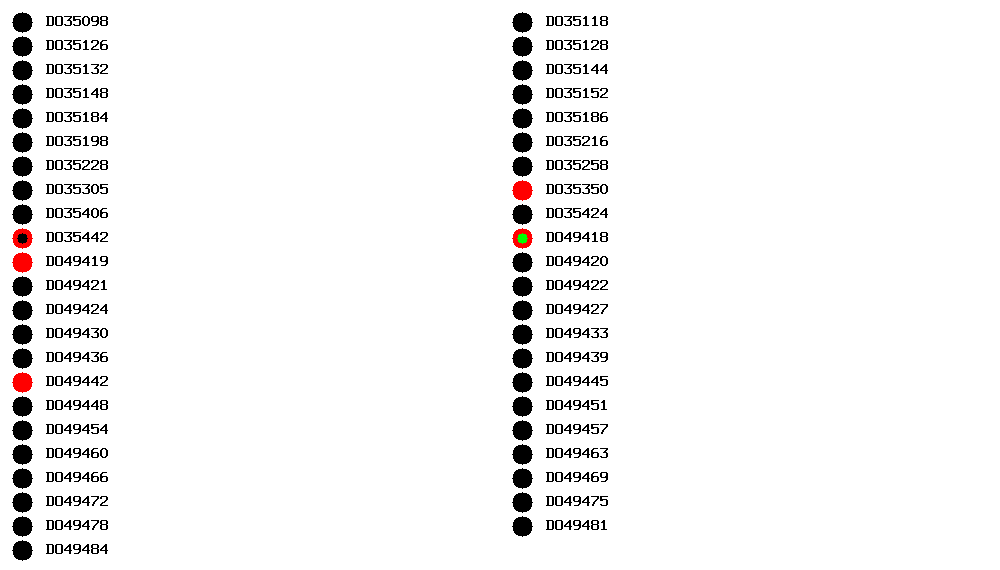


**Supplemental Figure 20**

| DO35442 | DO49418 |
| --- | --- |
| 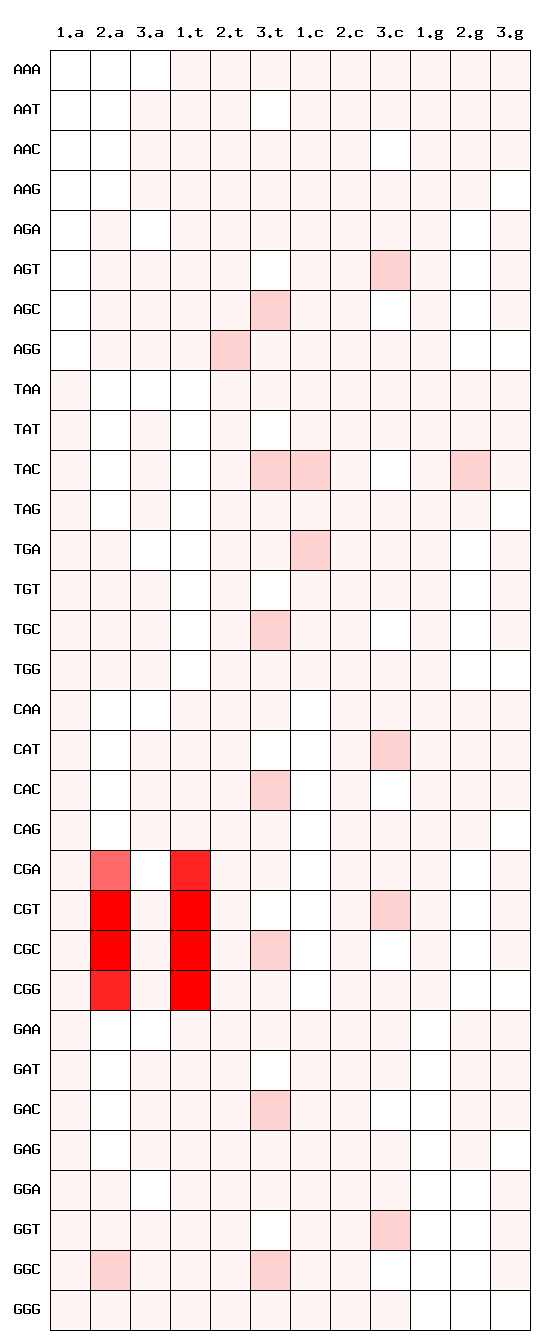 | 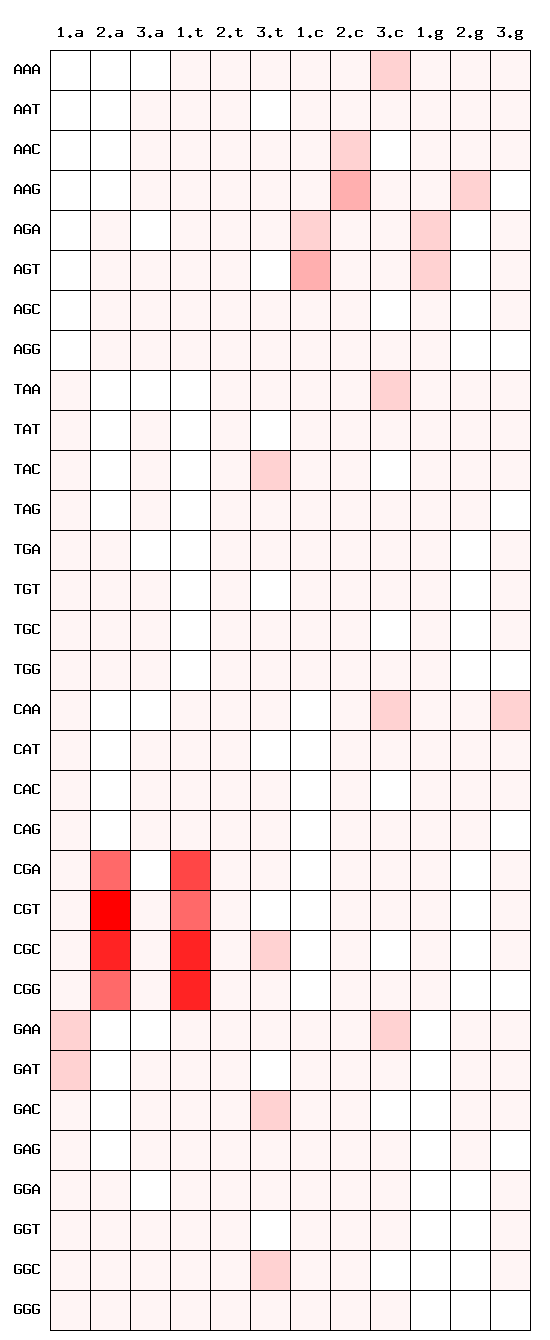 |
| \| Trinucleotide \| Mutation \| Fraction \| \| --- \| --- \| --- \| \| CGT \| 2.a \| 0.098039 \| \| CGC \| 2.a \| 0.090732 \| \| CGC \| 1.t \| 0.084599 \| \| CGG \| 1.t \| 0.072644 \| \| CGT \| 1.t \| 0.068120 \| | \| Trinucleotide \| Mutation \| Fraction \| \| --- \| --- \| --- \| \| CGT \| 2.a \| 0.076049 \| \| CGC \| 1.t \| 0.062796 \| \| CGC \| 2.a \| 0.058692 \| \| CGG \| 1.t \| 0.056858 \| \| CGA \| 1.t \| 0.051115 \| |

| DO49419 | DO49430 |
| --- | --- |
| 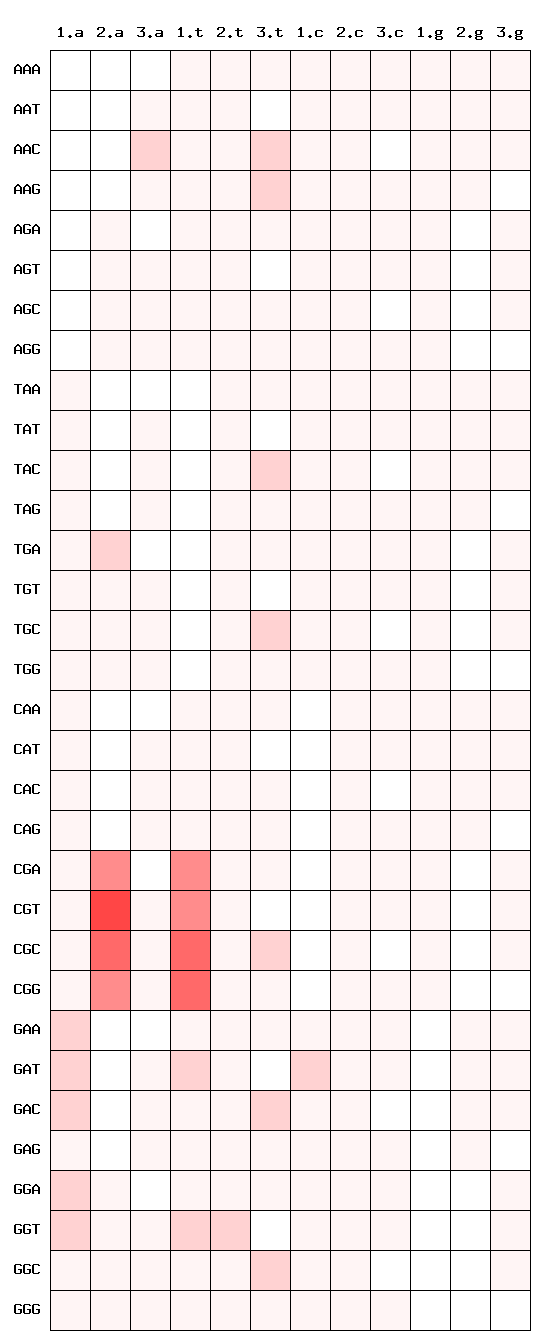 | 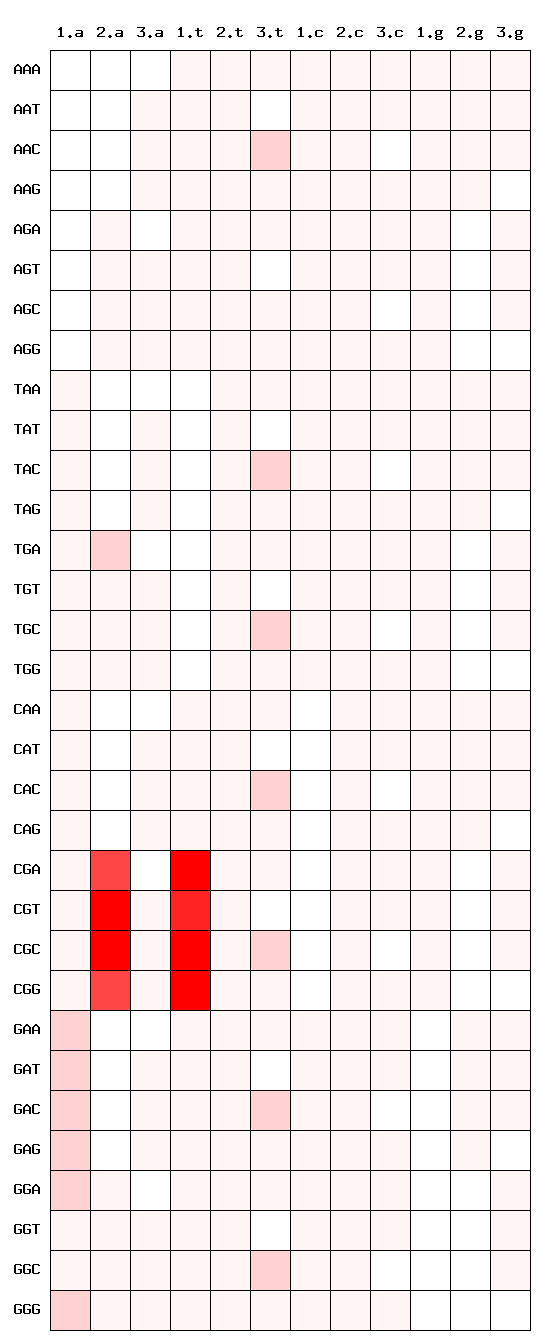 |
| \| Trinucleotide \| Mutation \| Fraction \| \| --- \| --- \| --- \| \| CGT \| 2.a \| 0.048247 \| \| CGC \| 1.t \| 0.040818 \| \| CGC \| 2.a \| 0.035953 \| \| CGG \| 1.t \| 0.035426 \| \| CGA \| 1.t \| 0.034197 \| | \| Trinucleotide \| Mutation \| Fraction \| \| --- \| --- \| --- \| \| CGT \| 2.a \| 0.099790 \| \| CGC \| 1.t \| 0.074811 \| \| CGC \| 2.a \| 0.072178 \| \| CGA \| 1.t \| 0.069737 \| \| CGG \| 1.t \| 0.068296 \| |

**Supplemental Figure 21**


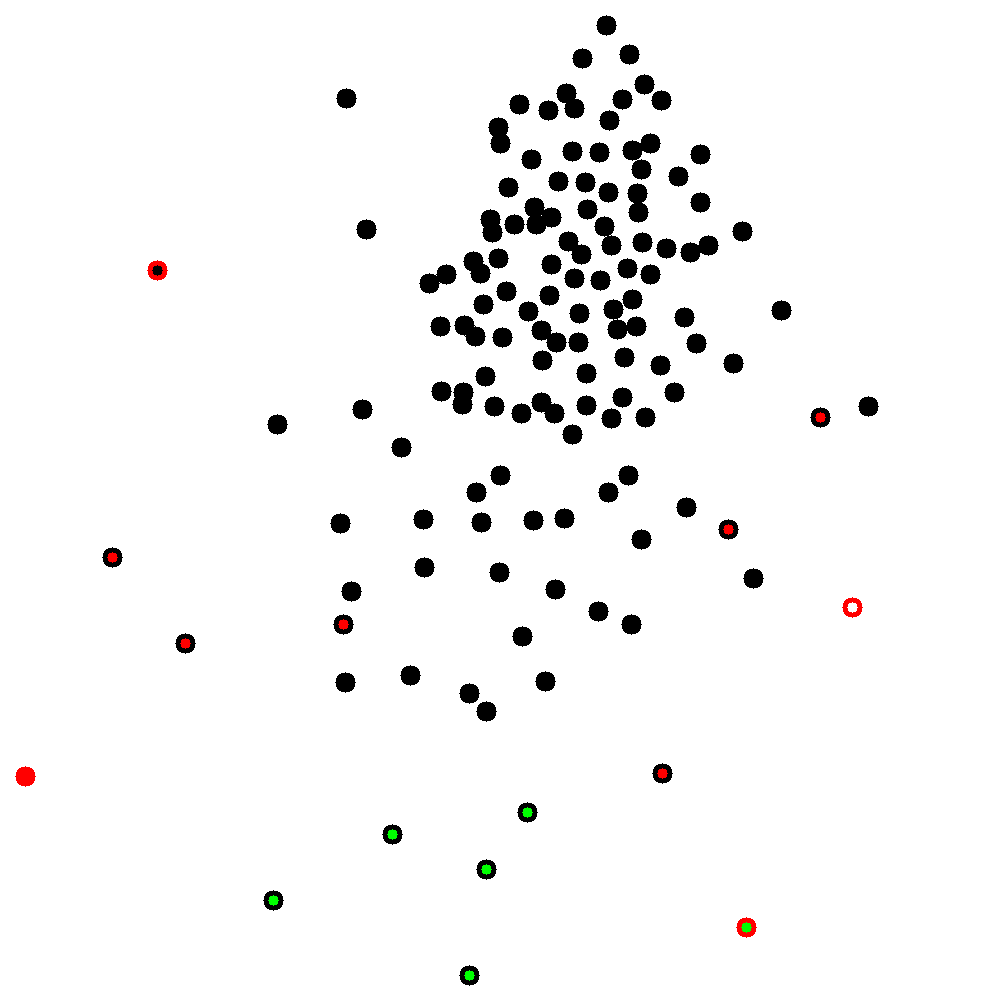


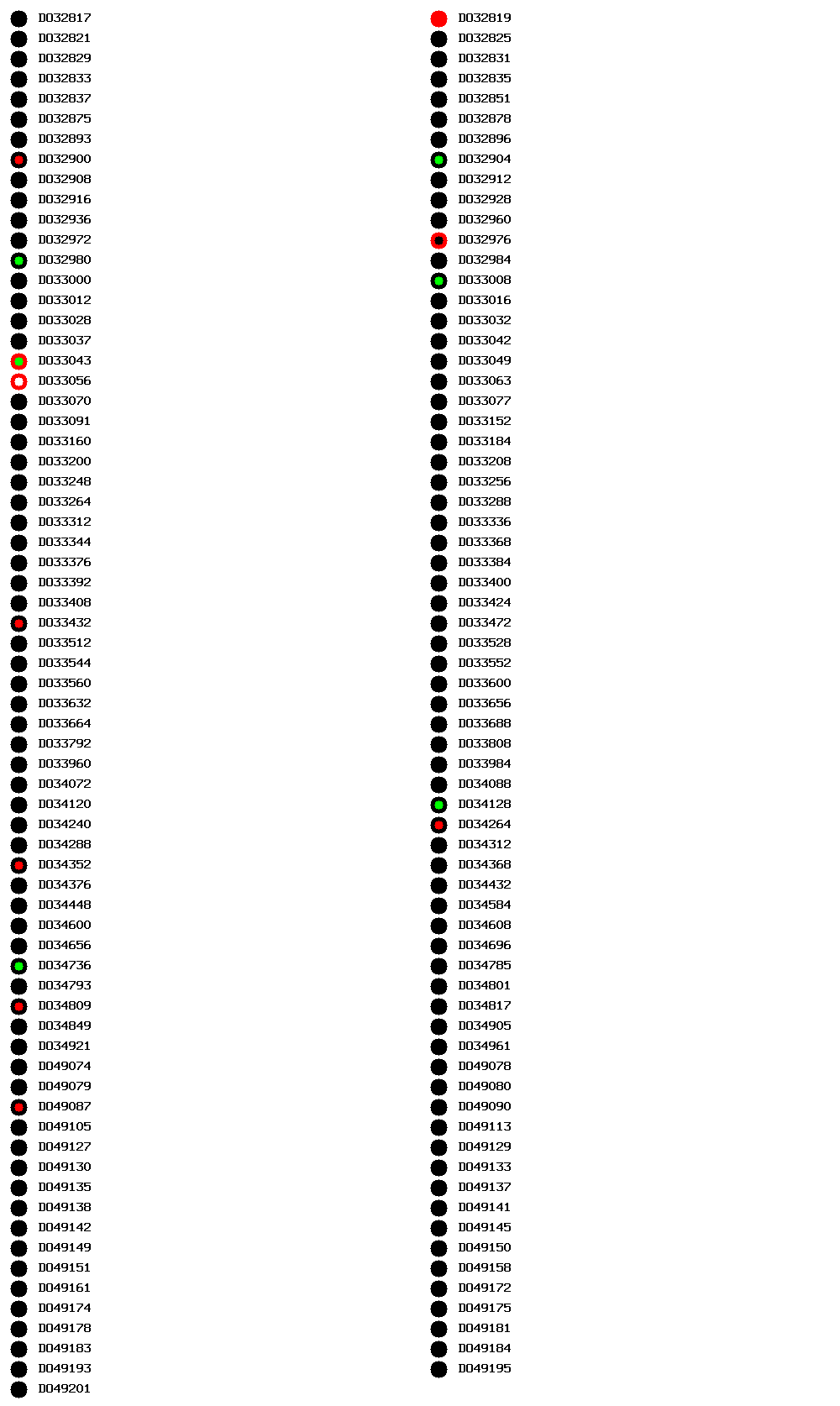


**Supplemental Figure 22**

| DO32819 | DO32976 | DO33043 |
| --- | --- | --- |
| 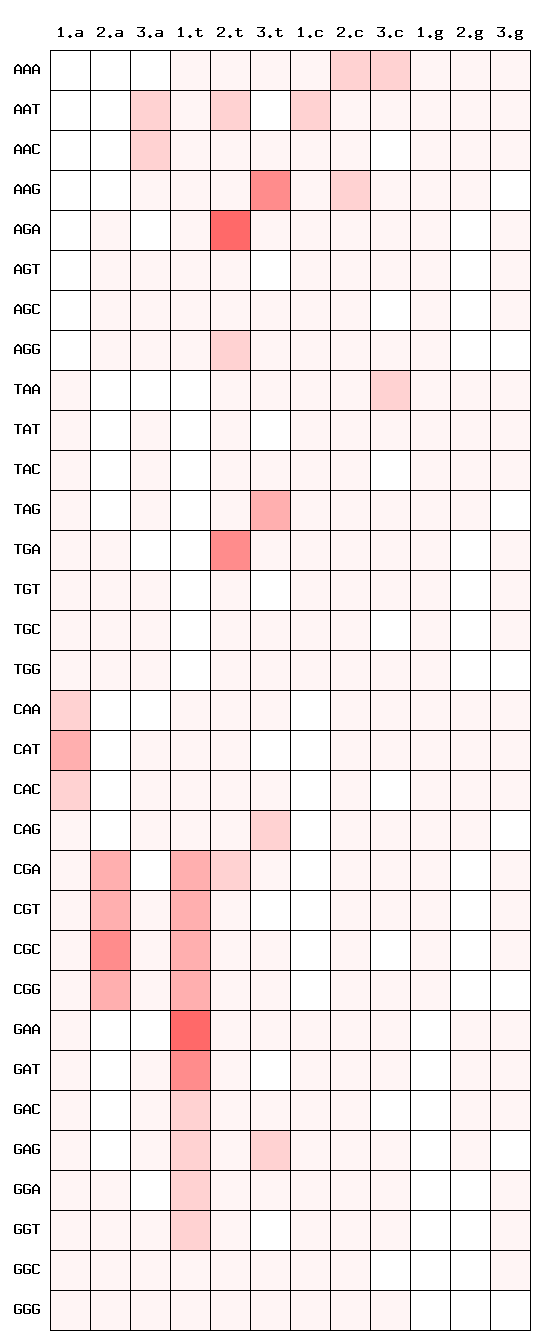 | 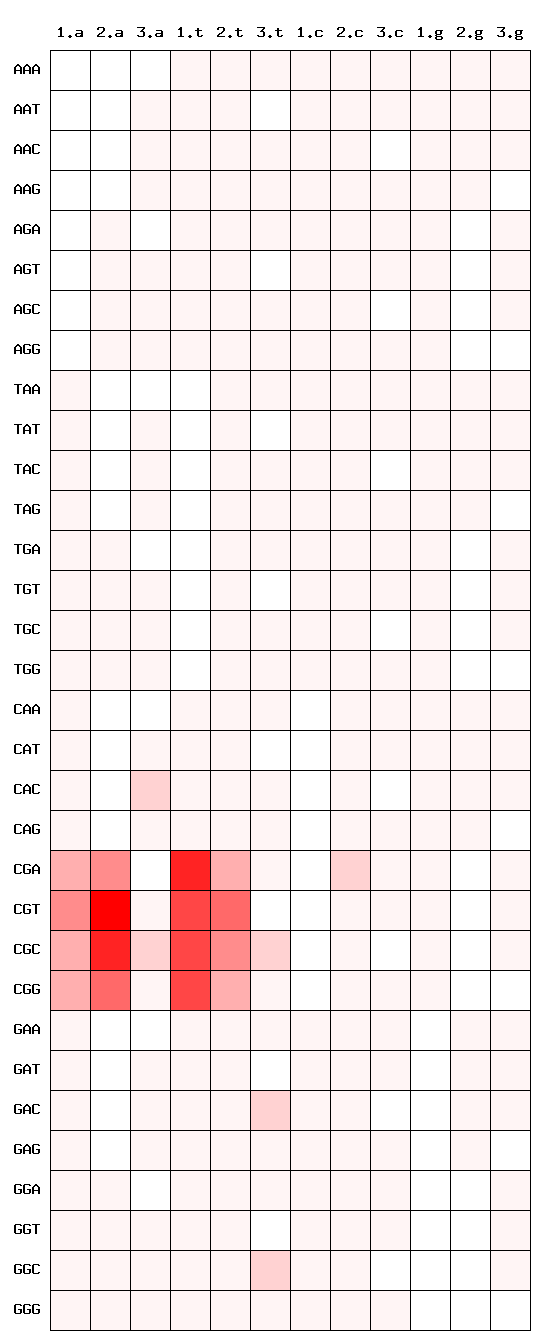 | 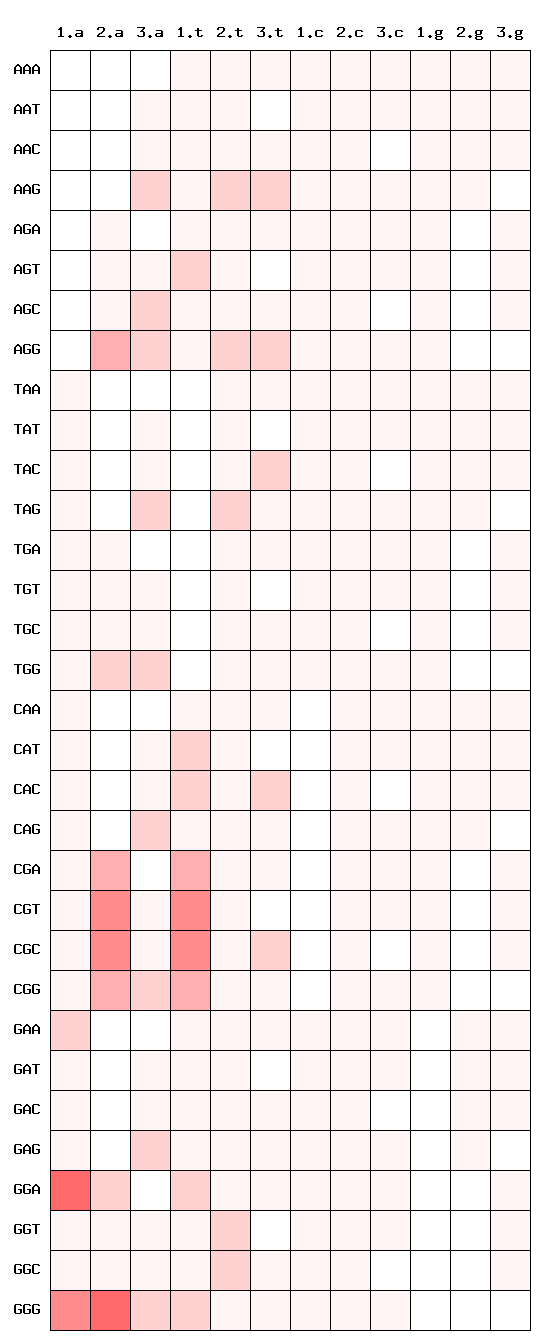 |
| \| Trinucleotide \| Mutation \| Fraction \| \| --- \| --- \| --- \| \| GAA \| 1.t \| 0.044409 \| \| AGA \| 2.t \| 0.042756 \| \| AAG \| 3.t \| 0.034254 \| \| CGC \| 2.a \| 0.027877 \| \| TGA \| 2.t \| 0.026912 \| | \| Trinucleotide \| Mutation \| Fraction \| \| --- \| --- \| --- \| \| CGT \| 2.a \| 0.073752 \| \| CGC \| 2.a \| 0.056480 \| \| CGA \| 1.t \| 0.055568 \| \| CGC \| 1.t \| 0.052848 \| \| CGT \| 1.t \| 0.047335 \| | \| Trinucleotide \| Mutation \| Fraction \| \| --- \| --- \| --- \| \| GGA \| 1.a \| 0.044816 \| \| GGG \| 2.a \| 0.036694 \| \| CGT \| 2.a \| 0.033291 \| \| CGC \| 2.a \| 0.026159 \| \| CGC \| 1.t \| 0.026159 \| |

| DO33056 | DO34128 | DO49087 |
| --- | --- | --- |
|  |  |  |
| \| Trinucleotide \| Mutation \| Fraction \| \| --- \| --- \| --- \| \| CGT \| 2.a \| 0.065783 \| \| GGT \| 3.g \| 0.062990 \| \| CGC \| 2.a \| 0.051931 \| \| CGA \| 1.t \| 0.048919 \| \| CGC \| 1.t \| 0.048070 \| | \| Trinucleotide \| Mutation \| Fraction \| \| --- \| --- \| --- \| \| CGT \| 2.a \| 0.036107 \| \| CGA \| 1.t \| 0.032509 \| \| CGC \| 2.a \| 0.029892 \| \| CGG \| 1.t \| 0.029885 \| \| CGC \| 1.t \| 0.025253 \| | \| Trinucleotide \| Mutation \| Fraction \| \| --- \| --- \| --- \| \| CGT \| 2.a \| 0.073644 \| \| CGC \| 2.a \| 0.061129 \| \| CGC \| 1.t \| 0.061129 \| \| CGA \| 1.t \| 0.055774 \| \| CGG \| 1.t \| 0.053735 \| |

| DO49130 |
| --- |
|  |
| \| Trinucleotide \| Mutation \| Fraction \| \| --- \| --- \| --- \| \| CGT \| 2.a \| 0.088049 \| \| CGC \| 2.a \| 0.077393 \| \| CGC \| 1.t \| 0.074039 \| \| CGA \| 1.t \| 0.070357 \| \| CGG \| 1.t \| 0.068435 \| |

**Supplemental Figure 23**

| DO46779 | DO46749 |
| --- | --- |
|  |  |
| \| Trinucleotide \| Mutation \| Fraction \| \| --- \| --- \| --- \| \| GAA \| 1.t \| 0.050968 \| \| AGA \| 2.t \| 0.049863 \| \| GAT \| 1.t \| 0.049634 \| \| AAG \| 3.t \| 0.036316 \| \| TGA \| 2.t \| 0.033418 \| | \| Trinucleotide \| Mutation \| Fraction \| \| --- \| --- \| --- \| \| CGT \| 2.a \| 0.101128 \| \| CGA \| 1.t \| 0.075549 \| \| CGC \| 1.t \| 0.071467 \| \| CGC \| 2.a \| 0.069968 \| \| CGG \| 1.t \| 0.069430 \| |

| DO46785 | DO48581 |
| --- | --- |
|  |  |
| \| Trinucleotide \| Mutation \| Fraction \| \| --- \| --- \| --- \| \| CGT \| 2.a \| 0.032614 \| \| CGC \| 1.t \| 0.026129 \| \| CGC \| 2.a \| 0.022109 \| \| CGA \| 1.t \| 0.021130 \| \| CGT \| 1.t \| 0.020464 \| | \| Trinucleotide \| Mutation \| Fraction \| \| --- \| --- \| --- \| \| CGT \| 2.a \| 0.040898 \| \| CGA \| 1.t \| 0.033595 \| \| CGC \| 1.t \| 0.032884 \| \| CGC \| 2.a \| 0.029894 \| \| CGG \| 2.a \| 0.027515 \| |

**Supplemental Figure 24**

| APGI_2137 | APGI_2157 |
| --- | --- |
|  |  |
| \| Trinucleotide \| Mutation \| Fraction \| \| --- \| --- \| --- \| \| CGC \| 2.a \| 0.046230 \| \| CGT \| 2.a \| 0.045187 \| \| CGC \| 1.t \| 0.042258 \| \| CGA \| 1.t \| 0.040256 \| \| CGG \| 1.t \| 0.036601 \| | \| Trinucleotide \| Mutation \| Fraction \| \| --- \| --- \| --- \| \| CGT \| 2.a \| 0.074299 \| \| CGA \| 1.t \| 0.061589 \| \| CGG \| 1.t \| 0.059665 \| \| CGC \| 1.t \| 0.058753 \| \| CGA \| 2.a \| 0.055361 \| |

| APGI_2057 | APGI_2353 |
| --- | --- |
|  |  |
| \| Trinucleotide \| Mutation \| Fraction \| \| --- \| --- \| --- \| \| CGT \| 2.a \| 0.030224 \| \| CGG \| 1.t \| 0.025283 \| \| CGC \| 2.a \| 0.024570 \| \| CGA \| 1.t \| 0.022863 \| \| CGC \| 1.t \| 0.022074 \| | \| Trinucleotide \| Mutation \| Fraction \| \| --- \| --- \| --- \| \| CGT \| 2.a \| 0.080847 \| \| CGC \| 2.a \| 0.067840 \| \| CGC \| 1.t \| 0.063963 \| \| CGG \| 1.t \| 0.062385 \| \| CGA \| 1.t \| 0.057126 \| |

**Supplemental Figure 25**

| MB110 | MB34 | MB101 |
| --- | --- | --- |
|  |  |  |
| \| Trinucleotide \| Mutation \| Fraction \| \| --- \| --- \| --- \| \| CGT \| 2.a \| 0.037407 \| \| CGC \| 2.a \| 0.031675 \| \| CGA \| 1.t \| 0.030945 \| \| CGC \| 1.t \| 0.026307 \| \| CGG \| 1.t \| 0.025016 \| | \| Trinucleotide \| Mutation \| Fraction \| \| --- \| --- \| --- \| \| CGT \| 2.a \| 0.090897 \| \| CGA \| 1.t \| 0.061200 \| \| CGT \| 1.t \| 0.058762 \| \| CGC \| 1.t \| 0.055792 \| \| CGC \| 2.a \| 0.054831 \| | \| Trinucleotide \| Mutation \| Fraction \| \| --- \| --- \| --- \| \| CGT \| 2.a \| 0.121138 \| \| CGC \| 1.t \| 0.078632 \| \| CGA \| 1.t \| 0.071638 \| \| CGG \| 1.t \| 0.064875 \| \| CGC \| 2.a \| 0.064492 \| |

**Supplemental Figure 26**

| DO35739 | DO48901 |
| --- | --- |
|  |  |
| \| Trinucleotide \| Mutation \| Fraction \| \| --- \| --- \| --- \| \| CGT \| 2.a \| 0.042762 \| \| CGA \| 1.t \| 0.037198 \| \| CGC \| 2.a \| 0.035649 \| \| CGG \| 1.t \| 0.029514 \| \| CGC \| 1.t \| 0.027459 \| | \| Trinucleotide \| Mutation \| Fraction \| \| --- \| --- \| --- \| \| CGT \| 2.a \| 0.070961 \| \| CGA \| 1.t \| 0.054939 \| \| CGG \| 1.t \| 0.047428 \| \| CGC \| 1.t \| 0.043844 \| \| CGT \| 1.t \| 0.043062 \| |

| DO35589 | DO48909 |
| --- | --- |
|  |  |
| \| Trinucleotide \| Mutation \| Fraction \| \| --- \| --- \| --- \| \| CGT \| 2.a \| 0.093200 \| \| CGG \| 1.t \| 0.071509 \| \| CGC \| 2.a \| 0.068099 \| \| CGC \| 1.t \| 0.056569 \| \| CGT \| 1.t \| 0.055714 \| | \| Trinucleotide \| Mutation \| Fraction \| \| --- \| --- \| --- \| \| CGT \| 2.a \| 0.132309 \| \| CGC \| 1.t \| 0.086999 \| \| CGA \| 1.t \| 0.076323 \| \| CGG \| 1.t \| 0.069816 \| \| CGC \| 2.a \| 0.067860 \| |

**Supplemental Figure 27**

| DO36221 | DO36223 | DO48577 |
| --- | --- | --- |
|  |  |  |
| \| Trinucleotide \| Mutation \| Fraction \| \| --- \| --- \| --- \| \| CGT \| 2.a \| 0.102700 \| \| CGC \| 2.a \| 0.073517 \| \| CGC \| 1.t \| 0.073517 \| \| CGA \| 1.t \| 0.068304 \| \| CGG \| 1.t \| 0.065538 \| | \| Trinucleotide \| Mutation \| Fraction \| \| --- \| --- \| --- \| \| CGT \| 2.a \| 0.095952 \| \| CGC \| 1.t \| 0.070481 \| \| CGA \| 1.t \| 0.070196 \| \| CGG \| 1.t \| 0.069139 \| \| CGC \| 2.a \| 0.063433 \| | \| Trinucleotide \| Mutation \| Fraction \| \| --- \| --- \| --- \| \| CGT \| 2.a \| 0.094090 \| \| CGC \| 2.a \| 0.074670 \| \| CGC \| 1.t \| 0.074144 \| \| CGA \| 1.t \| 0.068150 \| \| CGG \| 1.t \| 0.066340 \| |

**Supplemental Figure 28**

| PR-1701 | PR-3043 |
| --- | --- |
|  |  |
| \| Trinucleotide \| Mutation \| Fraction \| \| --- \| --- \| --- \| \| CGT \| 2.a \| 0.063018 \| \| CGA \| 1.t \| 0.055315 \| \| CGC \| 2.a \| 0.050863 \| \| CGC \| 1.t \| 0.049395 \| \| CGG \| 1.t \| 0.042201 \| | \| Trinucleotide \| Mutation \| Fraction \| \| --- \| --- \| --- \| \| CGT \| 2.a \| 0.042844 \| \| CGC \| 2.a \| 0.037982 \| \| CGA \| 1.t \| 0.037552 \| \| CGC \| 1.t \| 0.037291 \| \| CGT \| 1.t \| 0.030980 \| |

**Supplemental Figure 29**

**Supplemental Figure 30**

| DO46877 | DO46897 | DO46941 |
| --- | --- | --- |
|  |  |  |
| \| Trinucleotide \| Mutation \| Fraction \| \| --- \| --- \| --- \| \| CGT \| 2.a \| 0.102303 \| \| CGC \| 1.t \| 0.070689 \| \| CGT \| 1.t \| 0.066451 \| \| CGC \| 2.a \| 0.066040 \| \| CGA \| 1.t \| 0.060025 \| | \| Trinucleotide \| Mutation \| Fraction \| \| --- \| --- \| --- \| \| TAG \| 2.t \| 0.046198 \| \| CAG \| 2.t \| 0.038562 \| \| AGG \| 1.t \| 0.033684 \| \| TAG \| 1.a \| 0.030037 \| \| TGT \| 1.a \| 0.029956 \| | \| Trinucleotide \| Mutation \| Fraction \| \| --- \| --- \| --- \| \| CGC \| 1.t \| 0.021859 \| \| CGA \| 1.t \| 0.020276 \| \| CGT \| 2.a \| 0.019636 \| \| CGA \| 2.a \| 0.018179 \| \| TAG \| 2.t \| 0.017267 \| |

| DO46905 | DO47174 |
| --- | --- |
|  |  |
| \| Trinucleotide \| Mutation \| Fraction \| \| --- \| --- \| --- \| \| TAG \| 2.t \| 0.036604 \| \| TAG \| 1.a \| 0.026015 \| \| TAT \| 1.a \| 0.025166 \| \| AGG \| 1.t \| 0.021680 \| \| CAG \| 2.t \| 0.020756 \| | \| Trinucleotide \| Mutation \| Fraction \| \| --- \| --- \| --- \| \| CGT \| 2.a \| 0.035564 \| \| CGA \| 1.t \| 0.030220 \| \| CGC \| 2.a \| 0.023998 \| \| CGC \| 1.t \| 0.023998 \| \| CGG \| 1.t \| 0.022615 \| |
